# Synthesis of Chalcone Derivatives: Inducing Apoptosis of HepG2 Cells *via* Regulating Reactive Oxygen Species and Mitochondrial Pathway

**DOI:** 10.3389/fphar.2019.01341

**Published:** 2019-11-15

**Authors:** Hongtian Zhu, Lei Tang, Chenghong Zhang, Baochu Wei, Pingrong Yang, Dian He, Lifang Zheng, Yang Zhang

**Affiliations:** ^1^Materia Medica Development Group, Institute of Medicinal Chemistry, Lanzhou University School of Pharmacy, Lanzhou, China; ^2^Innovative Drug Research Department, Lanzhou Weihuan Biological Science and Technology Development Co, Ltd., Lanzhou, China; ^3^Pharmacy Department, Lanzhou Pulmonary Hospital Pharmacy, Lanzhou, China; ^4^NMPA Key Laboratory for Quality Control of Traditional Chinese Medicine (Chinese Materia Medica and Prepared Slices), Gansu Institute for Drug Control, Lanzhou, China; ^5^School of Pharmaceutical Sciences, Chongqing University, Chongqing, China

**Keywords:** chalcone, HepG2 cell, reactive oxygen species, mitochondrial membrane potential, B-cell lymphoma-2, Caspase 3

## Abstract

Chalcone derivatives, as a hot research field, exhibit a variety of physiological bioactivities and target multiple biological receptors. Based on the skeleton of (*E*)-1,3-diphenyl-2-propene-1-one, 14 chalcone derivatives were designed and synthesized, and evaluated as the antitumor candidates agents against four human cancer cell lines (A549, Hela, HepG2, and HL-60) as well as one normal cell line (WI-38). Among the title compounds, compound **a14** showed better inhibitory activity against HepG2 cells (IC_50_ = 38.33 µM) and had relatively weak cytotoxicity towards normal cells WI-38 (IC_50_ = 121.29 µM). In this study, apoptosis, cycle arrest, assessment of reactive oxygen species (ROS) level, and measurement of mitochondrial membrane potential were adopted to explore the inhibitory mechanism of **a14** towards HepG2. Compound **a14** could effectively block the division of HepG2 cell lines in the G2/M phase and robustly induced generation of ROS, demonstrating that the generation of ROS induced by **a14** was the main reason for resulting in the apoptosis of HepG2 cells. Moreover, the mitochondrial membrane potential (MMP) of HepG2 cells treated with **a14** was significantly decreased, which was closely related to the enhanced ROS level. Furthermore, based on Western blot experiment, cell apoptosis induced by **a14** also involved the expression of B-cell lymphoma-2 (Bcl-2) family and Caspase 3 protein. In summary, compound **a14** could contribute to the apoptosis of HepG2 cells through regulating ROS-mitochondrial pathway, which provides valuable hints for the discovery of novel anti-tumor drug candidates.

## Introduction

Recently, more and more researchers have realized that nature is a potential resource of new therapeutic candidate compounds with diverse molecular skeletons, and an increasing number of studies have shown that natural products or their derivatives play a vital role in the treatment of various diseases (da Rocha et al., 2001; Cao et al., 2018; Jin et al., 2018), making the application of raw materials to synthesize the natural product analogs become much more indispensable (Koehn and Carter, 2005; Cai et al., 2012; Sahu et al., 2012; Singh et al., 2014; Yang et al., 2014; Zhang et al., 2015; Zhang et al., 2019). Among these raw materials, chalcone derivatives are the precursors used for the synthesis of flavonoids, which have been applied as anti-diabetic (Zhang et al., 2015), antiplatelet, anti-inflammatory (Lin et al., 2019), anti-allergic, antimicrobial (Yibcharoenporn et al., 2019), antioxidant, and anti-cancer agents (Cai et al., 2012) due to their simple chemical structures, simplicity of synthesis, and multiple modification sites in the skeletons (replaceable hydrogens) (Sahu et al., 2012; Coskun et al., 2017).

With the deepening of research, chalcone derivatives have shown excellent therapeutic effects in the treatment of many diseases, especially cancers (Padhye et al., 2009). In addition, due to the broad anti-tumor spectrum of chalcone derivatives, low toxicity to the normal cells, and immune enhancement, research on anti-tumor of such compounds has attracted widespread attention from more and more experts and scholars (Reddy et al., 2008; Loa et al., 2009; Zhou et al., 2009). Until now, several pure chalcone derivatives isolated from different plants have been applied in the clinical trials for treating viral, cardiovascular disorders, and especially the conservative treatment of malignant tumors, which demonstrate the research potential of such compounds (Batovska and Todorova, 2010; Sahu et al., 2012; Zhuang et al., 2017).

Moreover, many studies have explored the molecular mechanism of anti-tumor effects of chalcone derivatives, and it is necessary to note that chalcone derivatives can inhibit angiogenesis, induce caspase-dependent apoptotic cell death, and regulate the expression of pro-apoptotic proteins and anti-apoptotic cells related to the Bcl family (Zi and Simoneau, 2005; George et al., 2007; Wani et al., 2016). In addition, the cellular physicochemical properties of tumor cells make the mitochondria more susceptible to the influence and interference of chalcone derivatives (El-Meligie et al., 2017; Hawash et al., 2017; Makhdoumi et al., 2017; Karimi-Sales et al., 2018; Yan et al., 2019). Besides, the reactive oxygen species (ROS) is closely related to mitochondria of tumor cells, and the high levels of ROS will oxidize and nitrate macromolecules (Chen et al., 2011; Radhakrishnan et al., 2016; Li et al., 2018), further impairing the biological function of mitochondria (Sahu et al., 2012). In addition, chalcone derivatives have the ability to inhibit angiogenesis and induce caspase-dependent apoptotic cell death (Casaschi et al., 2004; Park et al., 2015; Ramirez-Tagle et al., 2016; Gomes et al., 2017). Therefore, it is very necessary to design and synthesize a series of chalcone derivatives and study their mechanisms of promoting apoptosis of tumor cell.

In this study, on the basis of the skeleton of chalcone ([2E]-1, 3-Diphenylprop-2-en-1-one), classical Claisen Schmidt condensation was applied to synthesize the target compounds using benzaldehyde and acetophenone as the raw materials under acidic and alkaline environments. Generally, the aryl rings of a large number of natural chalcone compounds (*e.g.* Isobavachalcone, Xanthoangelol) are polyhydroxylated (Yu et al., 2019), and ring A substituted by hydroxyl and amide substituents in the molecular structure would increase solubility and enhance bioactivity. Thus, on the basis of retaining the ring A structure, 14 chalcone derivatives were designed and synthesized for the first time. In addition, the cytotoxic activities of the 14 synthesized chalcone derivatives were assessed by thiazolyl blue tetrazolium bromid (MTT) assay, and compound **a14** with three methoxyl substituents showed the strongest cytotoxic activity on hepatocellular carcinoma cells (HepG2). Furthermore, the mechanism of compound **a14** contributing to the apoptosis of HepG2 cells was studied, which could provide valuable information for the research on chalcone derivatives applied in the treatment of cancers.

## Materials and Methods

### Materials and Instruments

Dulbecco’s modified Eagle medium (DMEM) and fetal bovine serum (FBS) were purchased from Hyclone (Shanghai, China). DCFH-DA (2′, 7′-dichlorodi-hydrofluoresceindiacetate) and MTT (3-[4, 5-dimethylthiazol-2-yl]-2, 5-diphenyltetrazolium bromide) were obtained from Sigma (Beijing, China).

All antibodies were obtained from Affinity Biosciences (Changzhou, China). DAPI (4DCFH-DA [2′, 7′-dichlorodi-hydrofluoresceindiacetate],6-diamidino-2-phenylindole), N-acetyl-L-cysteine (NAC) and carbonyl cyanide m-chlorophenyl hydrazine (CCCP) were obtained from Beijing Solarbio Science & Technology Co., Ltd. Varian Mercury spectrometer operating at 400 MHz for ^1^H NMR and 100 MHz for ^13^C NMR was used to record the ^1^H NMR and ^13^C NMR spectra, and Orbitrap Elite (Thermo Scientific) mass spectrometer Bruker APEX II 47e mass spectrometer was applied to determine the ESI/HRMS spectra.

### Chemistry

5 mmol aldehyde and 5 mmol ketone were dissolved in absolute ethanol (10 ml) before the addition of 1.0 ml SOCl_2_, and the mixed solution was stirred at room temperature (25°C) for 24 h. 10 ml water was added into the reaction solution and then boiled, which removed the impurities soluble in water. The reaction was then naturally cooled to room temperature and the solvent was discarded through filtration. The filtered cake rinsed with iced ethanol was dissolved in acetone and filtered in vacuum. After that, the filtrate was added to a silica gel and dried, and separated by column chromatography with hexane:acetone = 1:1 as the eluent to obtain the target compounds.

### Compound Purity Determination

Chromatographic conditions: Eclipse XDB-C18 (250 mm × 4.6 mm, 5 µm) was used as the column model; Methanol:H_2_O (60:40, v/v) was applied as the mobile phase, and the flow rate was 1.0 ml/min with detection at 282 nm. The samples were weighed in volumetric flasks and dissolved in methanol to prepare the solution of 0.5 mg/ml. 10 µl solution of the samples was accurately taken and injected into the liquid chromatograph to record the chromatogram.

### Cell Viability Assay

HeLa, A549, HepG2, HL-60, and WI-38 cell lines were incubated in DMEM solution containing 10% FBS for 48 h. The incubated tumor cells were seeded into 96-well plates (5 × 10^3^ cells/well) to make them attach for 12 h. Afterwards, the cells were processed with the various concentrations of the synthetic compounds and 5-FU for 48 h. After removing the cell culture supernatant, 10 µl MTT (5 mg/ml) solution was dripped into the cells to generate a formazan product, which were dissolved by dimethyl sulfoxide (DMSO) 4 h later. Multifunction microplate reader (Bio-Rad Laboratories, Shanghai, China) was adopted to measure the absorbance at the wave length of 490 nm. The IC_50_ (the lowest drug concentration causing 50% of the tumor cells inhibition) was then calculated using GraphPad Prism Software (version 5.02) and used to indicate the cytotoxic effect of the target compounds on the selected tumor cells.

### Inhibition Curve of A14 on Hepg2 Cells Under Different Conditions

HepG2 cells (5 × 10^3^ cells/well) were seeded into the 96-well plates and kept to attach overnight. Then the cells were dealt with **a14** at different concentrations for 24, 48, and 72 h, respectively. After the incubation time, 10 µl MTT was added to each well, and the culture was continued in a sterile incubator at 37°C for 4 h under the environment of 5% CO_2_. Then, the supernatant medium was discarded, and the solid was dissolved in 200 µl DMSO. Then, a multifunction microplate reader (Bio-Rad Laboratories, Shanghai, China) was applied to record the absorbance at 490 nm. Cell growth inhibition rates at different concentrations were calculated and plotted using Origin Pro8 software. Each set of data represented the average value of three independent experiments.

### Apoptosis and Cycle Arrest of Hepg2 Cells Caused by A14

#### Morphology Analysis of Apoptotic Cells With Fluorescence Microscopy

Firstly, HepG2 cells (3 × 10^5^ cells/well) were seeded into six-well plates and kept to attach overnight. Then, the cells were incubated with **a14** at different concentrations (0, 30, 50, and 70 µM) for 48 h, washed by ice-cold PBS solution and fixed with 4% paraformaldehyde for 10 min at room temperature. The cells was processed by DAPI (10 µg/ml) for 15 min in the dark, and the residual DAPI was rinsed two more times by ice-cold PBS. Then, the cells were transferred on a glass slide, fixed with a coverslip and placed under a fluorescence microscope (Motic China Group Co., Ltd., Shenzhen, China) to be observed and photographed at an excitation wavelength of 480/30× nm and the emission wavelength of 515 nm. Condensation of chromatin and fragmentation of nuclei could be applied to determine the apoptotic cells.

#### Quantitative Detection of the Apoptotic Cell by Annexin V-Alexa Fluor 647/propidium iodide (PI) Staining

HepG2 cells were seeded into six-well plates (3 × 10^5^/well) overnight to allow the cells to grow adherently and then incubated with **a14** at the concentrations of 0, 30, 40, 50, 60 or 70 µM for 48 h. In order to assess apoptosis, the double Annexin V-Alexa Fluor 647/PI (Beijing Solarbio Science & Technology Co., Ltd) immunofluorescence labeling method was used. After the cells were collected by centrifugation, 5 µl of Annexin V-Alexa Fluor 647 and 10 µl of PI solution (20 ug/ml) were added to each well. The cells were mixed and incubated at room temperature for 15 min in the dark and then mixed with 400 µl of PBS. Then, a Beckman Coulter flow cytometer (Beckman Coulter, Inc. California) was used to monitor the fluorescence. Ten thousand events were collected for each sample, and the data were analyzed *via* Flow Jo-V10 software.

#### Analysis of Cell Cycle Arrest Using PI Staining

HepG2 cells were seeded into six-well plates (3 × 10^5^/well) overnight to allow the cells to grow adherently and then incubated with **a14** at the concentrations of 0, 30, 40, 50, 60 or 70 µM for 24 and 48 h. After the incubation time, the cells were collected by centrifugation (1000 r/min) and fixed overnight with 79% ethanol at 4°C, and then the cells were removed and washed three times with ice-cold PBS. Afterwards, the cells were added to RNase A (Beijing Solarbio Science & Technology Co., Ltd.), and incubated for 30 min at 37°C, after which PI (Beijing Solarbio Science & Technology Co., Ltd.) was added. The fluorescence was monitored by a Beckman Coulter flow cytometer (Beckman Coulter, Inc. California). Ten thousand events were collected per sample, and the data were analyzed using Modfit LT 5.0 software.

#### Measurement of Reactive Oxygen Species

HepG2 cells were seeded into six-well plates (3 × 10^5^ cells/well) overnight to make the cells grow adherently and then incubated with **a14** (50 µM) for 0, 3, 6, 8, 9, and 12 h. After that, the cells were added to 1 ml DCFH-DA (10 µM) and incubated for 30 min in the dark. After the cells were washed twice by the pre-cooled PBS, the cells were trypsinized and collected by centrifugation. The cells of the blank group were implemented the same experimental procedures, except for the process with **a14**. Besides, 5 mM N-acetyl-L-cysteine (NAC) was applied as anti-oxidative substance to prevent the oxidation of the cells, which could entirely block the production of ROS induced by **a14**. The generation levels of ROS were evaluated in fluorescence intensity (FL-1, 530 nm) by Beckman Coulter flow cytometer (Beckman Coulter, Inc. California). The data collected was analyzed using the CytExpert 2.0 software embedded in Beckman flow cytometry, and the histogram was plotted by Origin Pro8.

#### Detection of Changes in Mitochondrial Membrane Potential by JC-1 Probe.

JC-1 probe was used to detect changes in MMP. Firstly, HepG2 cells (3 × 10^5^ cells/well) were seeded into a six-well plate overnight for adherence, and then treated with various concentrations of **a14** for 48 h. Next, the cells were treated with JC-1 (2.5 µg/ml) at 37°C for 10 min in the dark and rinsed three times with PBS. Then, the six-well plate was placed under the fluorescence microscope (excitation 480/30× nm, emission 515 nm) to observe the fluorescence color and intensity of JC-1 in the cells. In addition, HepG2 cells (3 × 10^5^ cells/well) were inoculated on another six-well plate overnight to allow adherence, and then treated with **a14** (50 µM) for 0, 12, 24, 36, and 48 h, adding CCCP (without **a14**) for 20 min to completely lose the mitochondrial membrane potential. Cells were then incubated with JC-1 (2.5 µg/ml) at 37°C in the dark for 10 min and rinsed with PBS for three times. Fluorescence intensity of residual JC-1 in cells was measured by Beckman Coulter flow cytometer (FITC, 525/40BP). The data obtained was analyzed by CytExpert 2.0 software imbedded in the flow cytometry.

#### The Expression of Apoptotic Protein in Hepg2 Cells

HepG2 cells (3 × 10^5^ cells/well) were seeded into a six-well plate overnight for adherence, and then treated with **a14** (0, 30, 40, 50, 60, and 70 µM) for 48 h. After the cells were collected by centrifugation, they were lysed with the radio immunoprecipitation assay (RIPA) buffer (high) containing 50 mM Tris (pH = 7.4), 150 mM NaCl, 1% TritonX-100, 1% sodium deoxycholate, 0.1% sodium dodecyl sulfate (SDS), 2 mM sodium pyrophosphate, 25 mM β-glycerophosphate, 1 mM ethylenediaminetetraacetic acid (EDTA), 1 mM Na_3_VO_4_, and 0.5 ug/ml leupeptin (Beijing Solarbio Science & Technology Co., Ltd.).The extract was then centrifuged at 12,000 rpm for 30 min and the total protein concentration was quantified using the BCA kit (Beijing Solarbio Science & Technology Co., Ltd.). The protein extracts were mixed with the SDS-PAGE protein loading buffer (Beijing Solarbio Science & Technology Co., Ltd.) and then boiled in 100°C water bath for 10 min. The gel was gelatinized using an SDS Gel Kit (Beijing Solarbio Science & Technology Co., Ltd.) and protein extracts were separated by protein SDS-polyacrylamide gel electrophoresis and transferred to Immobilon-NC Membrane (Beijing Solarbio Science & Technology Co., Ltd.). After blocking with 5% nonfat dried milk in Tris-buffered saline (TBS) containing 1% Tween-20 for 90 min at room temperature, the membranes were incubated overnight with specific primary antibodies (Affinity Biosciences, Changzhou, China) at 4°C. After washing three times with TBST, they were incubated in secondary antibodies for 2 h and washed three times with TBST. The chemiluminescence analysis system was applied to label the target protein with a specific primary antibody and detect the target protein with a specific secondary antibody.

## Data Analysis and Statistics

The data were expressed as the means ± SE of at least three independent experiments. Statistical differences between the two groups of data were analyzed by one-way ANOVA. Unless otherwise indicated, there was a statistical difference between the two sets of data at *P* < 0.05.

## Results

### Chemical Synthesis of Chalcone Derivatives

**Scheme 1:**
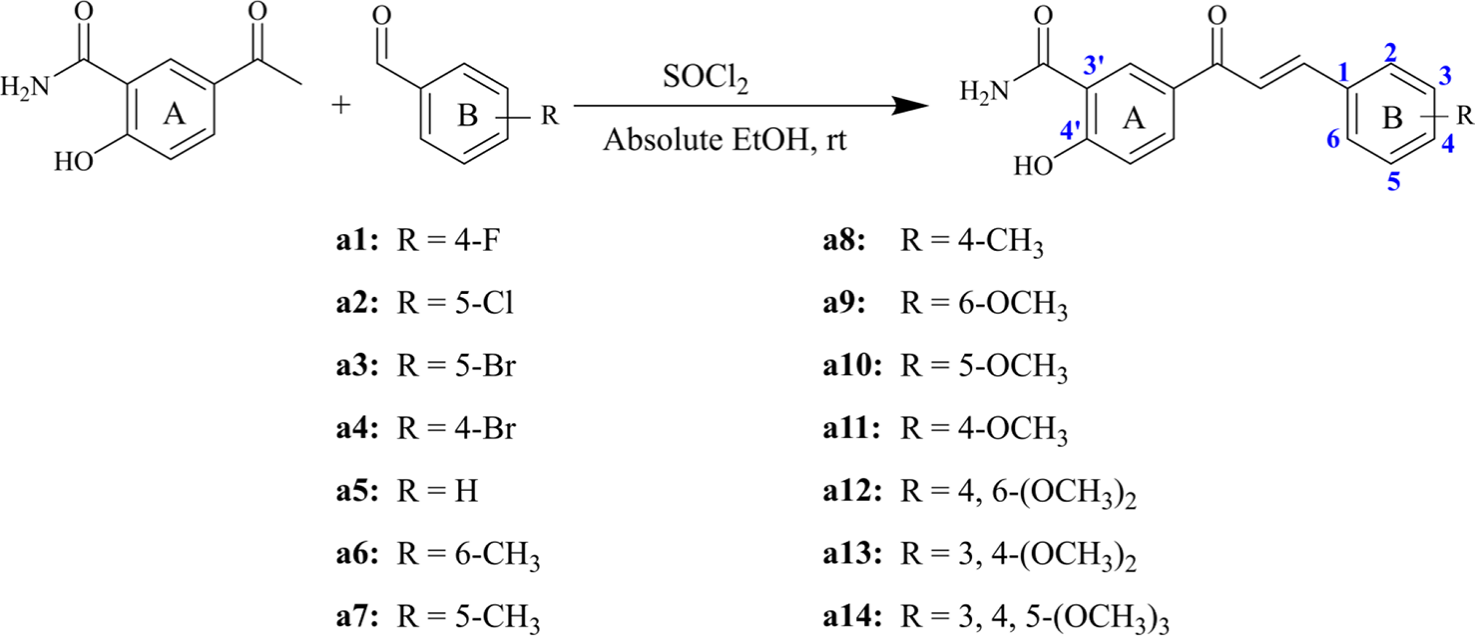
Synthesized compounds with different substituents.

Structurally, chalcone derivatives generally consist of two aryl groups (rings A and B) connected by an *α, β-*unsaturated ketone moiety, which forms the more thermodynamically stable trans-conformation (Zhang et al., 2017). Based on the skeleton, 14 chalcone derivatives were designed and synthesized, and **Scheme 1** showed the synthetic pathway. Due to the 2′-OH in the target compounds, sodium hydroxide cannot be applied as the catalyst for Claisen-Schmidt reaction. In this study, thionyl chloride (SOCl_2_) can react with ethanol (EtOH) to give off hydrogen chloride (Petrov et al., 2008; Hou et al., 2019), which would be used as the acid catalyst.

Briefly, different kinds of aldehydes were added to 5-acetyl-2-hydroxybenzamide in the condition of the mixed solutions of SOCl_2_ and EtOH, which will undergo Claisen-Schmidt condensation and produce the corresponding chalcone derivatives. The compounds (**a1-a14**) were reported herein for the first time, of which the structures were determined by HRMS, ^1^H-NMR, and ^13^C-NMR. Since all compounds were synthesized for the first time, no standard controls could be used as a reference. Based on this, the purities of the 14 compounds were analyzed by area normalization through HPLC, of which 12 compounds’ liquid phase purities were >94% and the remaining compounds were >91%. The information of HPLC, HRMS, ^1^H-NMR, and ^13^C-NMR of all compounds was attached to the **Supplementary Material**.

### Analysis of Cytotoxic Activity by Thiazolyl Blue Tetrazolium Bromid

**Figure 1:**
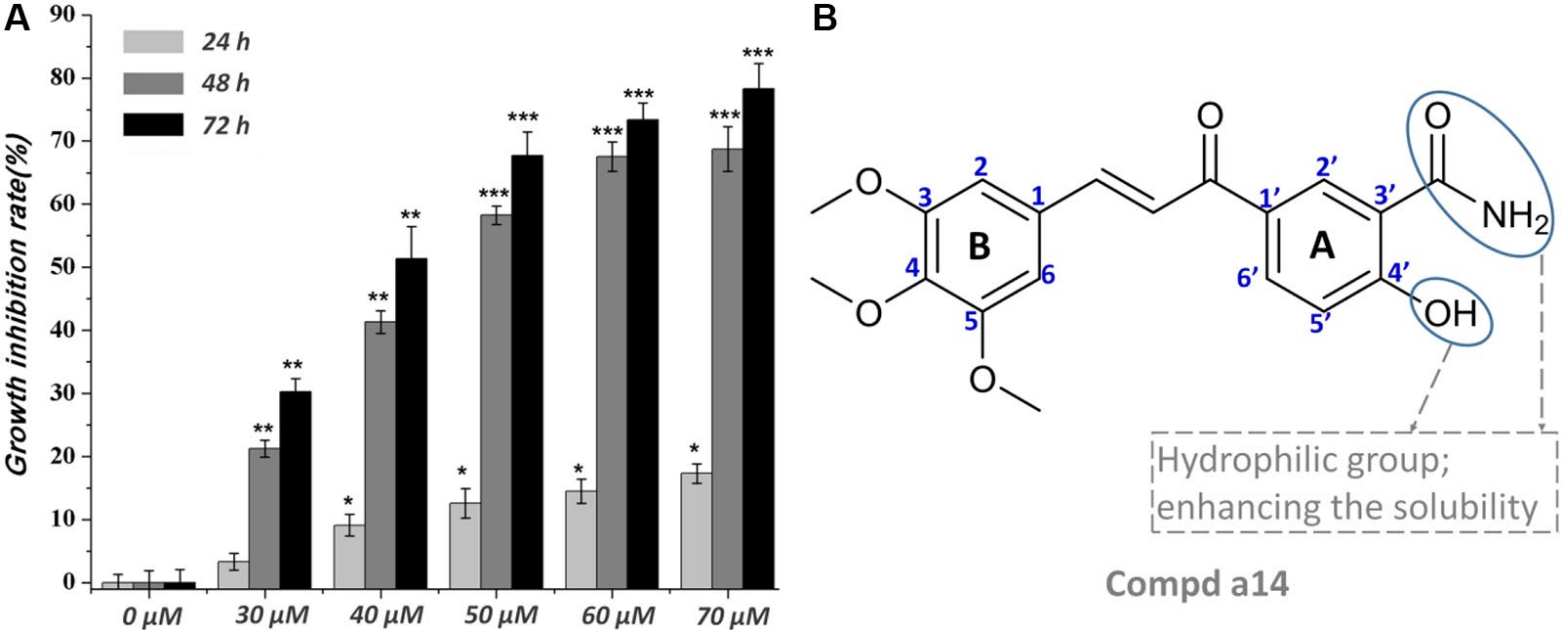
**(A)** Time and concentration-dependent effects of **a14** on HepG2 cells’ growth and viability. Values are expressed as the means ± SE, by *t* test, n = 3, *P< 0.05, **P< 0.01, ***P< 0.001 compared with the group (cell growth inhibition rate in 0 µM); **(B)** Chemical structure of **a14**.

The synthesized compounds were tested for the inhibitory activities towards A549, Hela, HepG2, and HL-60 and a normal cell line (WI-38) by &&MTT assay with 5-FU as the positive control. The results were summarized in **Table 1**, and showed that the 14 synthesized compounds exhibited significant cytotoxic effect on the adherent tumor cell lines (HepG2, A549 and Hela) as well as lower cytotoxic effect on the suspension cell (HL-60). In addition, according to the experimental results, it could be found that these compounds showed weaker cytotoxic effects on the normal cells (WI-38) than that of adherent tumor cells (HepG2, A549 and Hela). Moreover, **a14** showed better cytotoxic activities against HepG2 cells than 5-FU with relatively low toxicity towards human normal cells (WI-38), which was selected as a molecular probe to investigate the inhibitory mechanism of HepG2 cells through a series of biological activity experiments. According to **Figure 1**, **a14** could exhibit the inhibitory activities on HepG2 cells by a dose- and time-dependent manner.

**Table 1:** Biological activities of target compounds towards different cell lines.

Compds.	IC_50_ (µM)^a^				
	A549	Hela	HepG2	HL-60	WI-38
**a1**	107.35 ± 2.14	125.34 ± 6.92	134.64 ± 9.79	130.71 ± 5.38	229.04 ± 7.38
**a2**	82.46 ± 3.61	98.90 ± 2.90	76.96 ± 1.34	134.66 ± 2.45	122.2 ± 2.33
**a3**	59.59 ± 5.23	77.61 ± 4.74	71.21 ± 4.63	141.38 ± 5.19	126.24 ± 0.46
**a4**	48.17 ± 2.25	65.93 ± 7.80	46.41 ± 0.05	110.28 ± 2.04	100.24 ± 11.51
**a5**	57.25 ± 1.53	82.19 ± 4.80	68.13 ± 5.51	107.83 ± 3.50	127.04 ± 6.60
**a6**	51.34 ± 2.11	74.84 ± 2.33	75.41 ± 3.27	213.08 ± 2.98	109.48 ± 1.49
**a7**	40.72 ± 3.68	40.72 ± 1.33	55.62 ± 3.23	111.35 ± 1.57	81.70 ± 1.13
**a8**	64.95 ± 2.35	53.55 ± 0.67	57.35 ± 3.07	301.19 ± 3.53	147.31 ± 13.24
**a9**	57.63 ± 1.42	65.07 ± 1.34	80.31 ± 1.87	210.14 ± 3.03	98.46 ± 4.91
**a10**	45.55 ± 1.05	54.39 ± 1.11	70.80 ± 4.03	131.15 ± 4.64	93.28 ± 3.13
**a11**	35.84 ± 1.02	48.23 ± 0.56	44.71 ± 1.56	245.00 ± 5.54	89.48 ± 6.61
**a12**	41.22 ± 2.15	35.33 ± 2.13	54.37 ± 0.52	160.46 ± 3.59	122.98 ± 2.45
**a13**	37.62 ± 1.11	31.82 ± 1.62	50.33 ± 4.03	134.43 ± 4.44	117.69 ± 3.53
**a14**	36.70 ± 1.08	23.30 ± 2.35	38.33 ± 0.59	120.68 ± 3.58	121.29 ± 0.87
**5-FU^b^**	53.70 ± 1.16	35.21 ± 1.39	42.89 ± 2.88	165.13 ± 4.74	62.77 ± 2.03

a IC_50_ values represent mean ± SE from at least three independent experiments.

b Used as a reference.

### Apoptosis and Cycle Arrest of Hepg2 Cells Caused by A14

**Figure 2:**
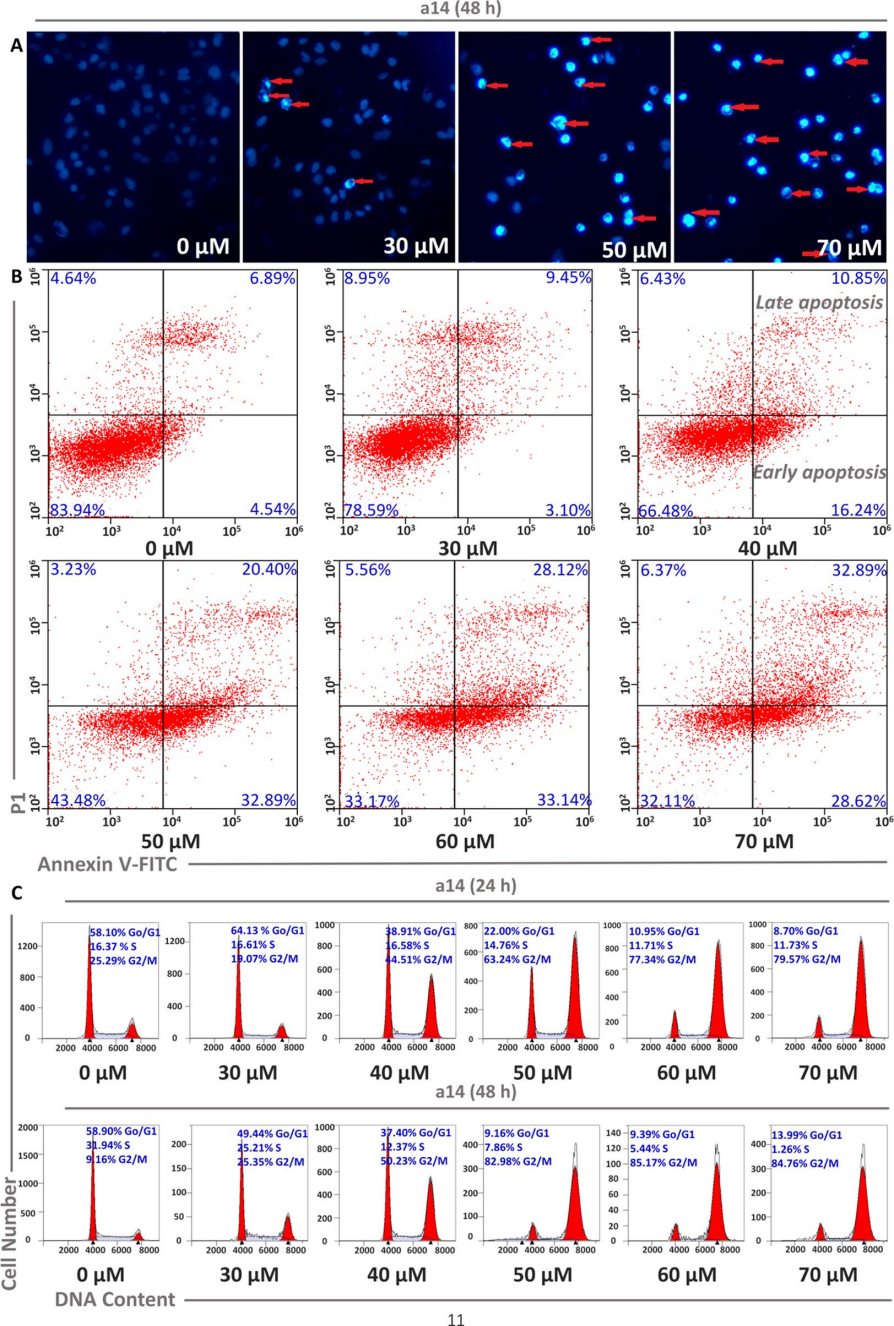
Concentration dependent effects of **a14** on HepG2 cell apoptosis. **(A)** HepG2 cells were stained with DAPI solution, and fluorescence microscope using a blue filter (magnification, 400×) was applied to observe the stained nuclei (red arrow indicating chromatin condensation); **(B)** Analysis of HepG2 cell apoptosis induced by **a14** using Annexin V-Alexa Fluor 647/PI immunofluorescence labeling method; **(C)** Analysis of cell cycle arrest by PI staining assay after 24 and 48 h incubation with **a14**.

Cell apoptosis and cycle arrest are the main approaches to investigate the mechanism of tumor cells growth inhibition and cell death induced by the compounds interference. In this study, corresponding experiments were performed to determine whether the cytotoxicity of **a14** on HepG2 cells was the result of apoptosis. HepG2 cells were dealt with various concentrations of **a14** for 48 h and the morphological changes of the nucleus could be detected by DAPI staining. In **Figure 2**, it could be seen that HepG2 cells had a large number of nuclear condensation and nuclear fragmentation (shown by red arrows) and emitted strong blue fluorescence under the intervention of **a14**, which indicated that HepG2 cells had undergone apoptosis. In addition, in order to further evaluate the apoptotic effects and **a14**, flow cytometry was conducted using double Annexin V-Alexa Fluor 647/PI immunofluorescence labeling method. After 48 h treatment of HepG2 cells with different concentrations of **a14** (30, 40, 50, 60, and 70 µM), the number of early-apoptotic cells increased from 3.10 to 28.62% and the number of late-apoptotic cells increased from 9.45 to 32.89%, which was positively correlated with the concentration of **a14** (**Figure 2**). In addition, it was worth pointing out that when the concentration of **a14** changed from 40 µM to 50 µM, the percentage of both early apoptosis and late apoptosis increase rapidly, indicating that most tumor cells underwent apoptosis at this concentration.

Moreover, PI staining was applied to assess the cycle block of **a14** on HepG2 cells, and in this study, HepG2 cells were treated with **a14** with the same concentration gradient mentioned above for 24 and 48 h, respectively. The number of HepG2 cells stagnated in G2/M phase ranged from 19.07 to 79.57% after the treatment with different concentrations of **a14**, and the blocking effects of **a14** on HepG2 cells were more obvious when the action time was prolonged to 48 h. From the above results, it could be concluded that **a14** mainly prevented HepG2 cells from dividing in the G2/M phase (**Figure 2**).

### Assessment of ROS Level in Hepg2 Cells Caused by A14

**Figure 3:**
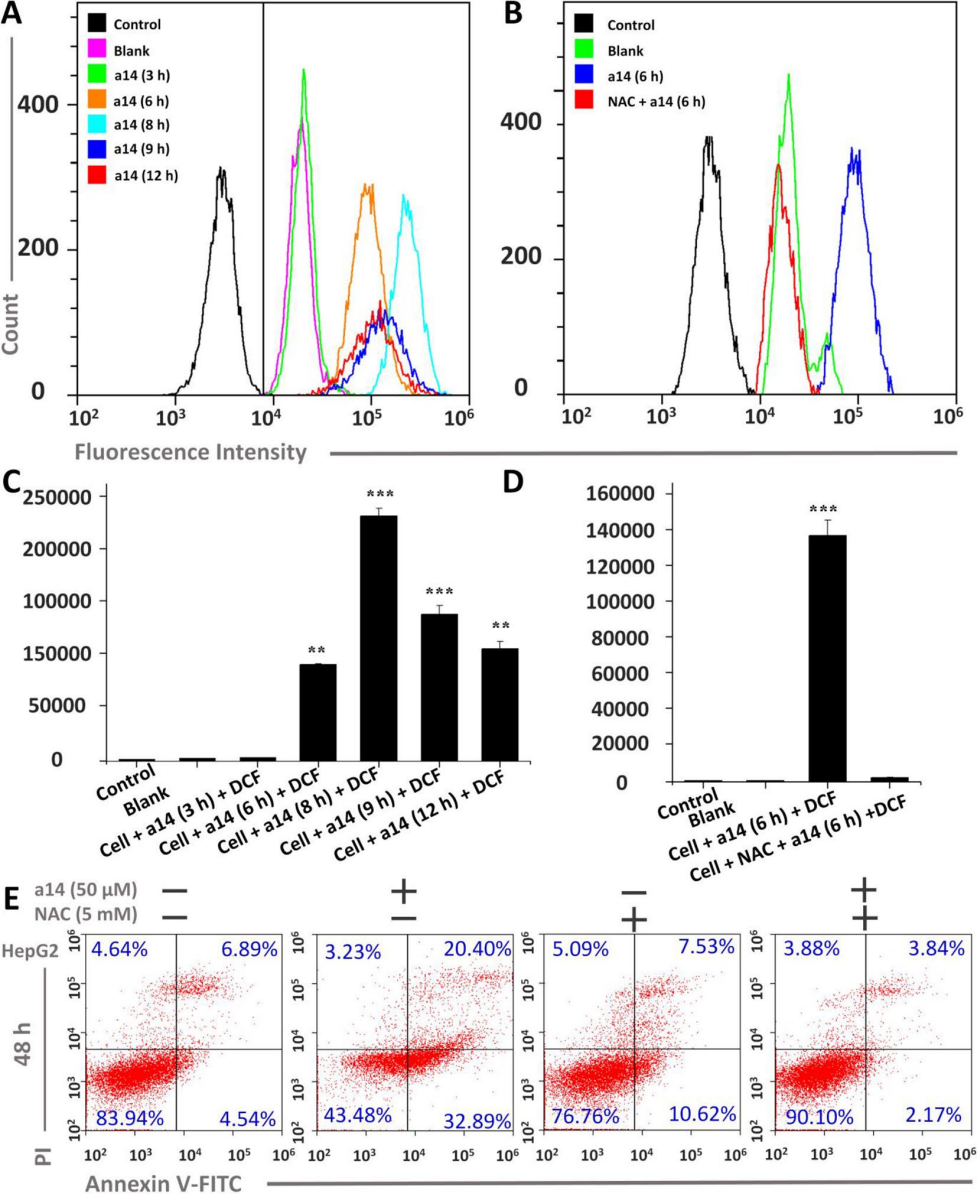
Changes in ROS levels induced by **a14** were probed by DCFH-DA in HepG2 cells. **(A)** Determination of fluorescence intensity in HepG2 cells under different conditions by flow cytometry; **(B)** Fluorescence intensity comparison chart with or without NAC; **(C)** Quantitative analysis of fluorescence intensity in HepG2 cells under different conditions; **(D)** Quantitative analysis of fluorescence intensity with or without NAC blockers; **(E)** Flow Cytometric analysis was applied to evaluate the pro-apoptosis of **a14** (50 µM) on HepG2 cells for 48 h with or without the intervention of NAC (5 mM). Values are expressed as the means ± SE, by *t* test, n = 3, **P < 0.01, ***P< 0.001 *vs*. the black group (cell + DCF).

To further research the mechanism underlying the pro-apoptosis activity of **a14**, the ROS level in HepG2 cells was assessed. In this study, HepG2 cells were treated with **a14** (50 µM) for 3, 6, 8, 9 and 12 h, and flow cytometry was applied to measure the intracellular fluorescence intensity using DCFH-DA probe, which could evaluate the levels of ROS production (**Figure 3**) (Cathcart et al., 1983). The experimental results showed that the intracellular ROS levels of HepG2 were significantly changed when stimulated by **a14** (50 µM). Compared to the control group, the intracellular fluorescence intensity of HepG2 increased continuously from 0 to 8 h(from 4351.6 to 236197.3) and began to decay after 8 h(**Figures 3A, C**), indicating that **a14** (50 µM) could induce a large amount of ROS production in HepG2 cells in a short period of time. However, if the HepG2 cells were pretreated with the antioxidant N-acetyl-L-cysteine (NAC, inhibiting the production of intracellular ROS) and then cultured with **a14**, there was no significant change in intracellular fluorescence intensity. Thus, it could be inferred that due to the action of **a14**, there was a significant transition in the level of ROS in HepG2 cells (**Figures 3B, D**).

Furthermore, the relationship between the HepG2 cells apoptosis and the burst of ROS was further explored. HepG2 cells were pretreated with NAC for 1 h, and co-cultured with **a14** (50 µM) for 48 h, and the number of apoptotic cells was determined by Annexin V-Alexa Fluor 647/PI double staining. It was found that the number of early apoptotic cells in the group treated by NAC and **a14** was significantly reduced compared to the group cultured only with **a14** (**Figure 3**).

### Influences on Mitochondrial Membrane Potential in Hepg2 Cells Induced by A14

**Figure 4:**
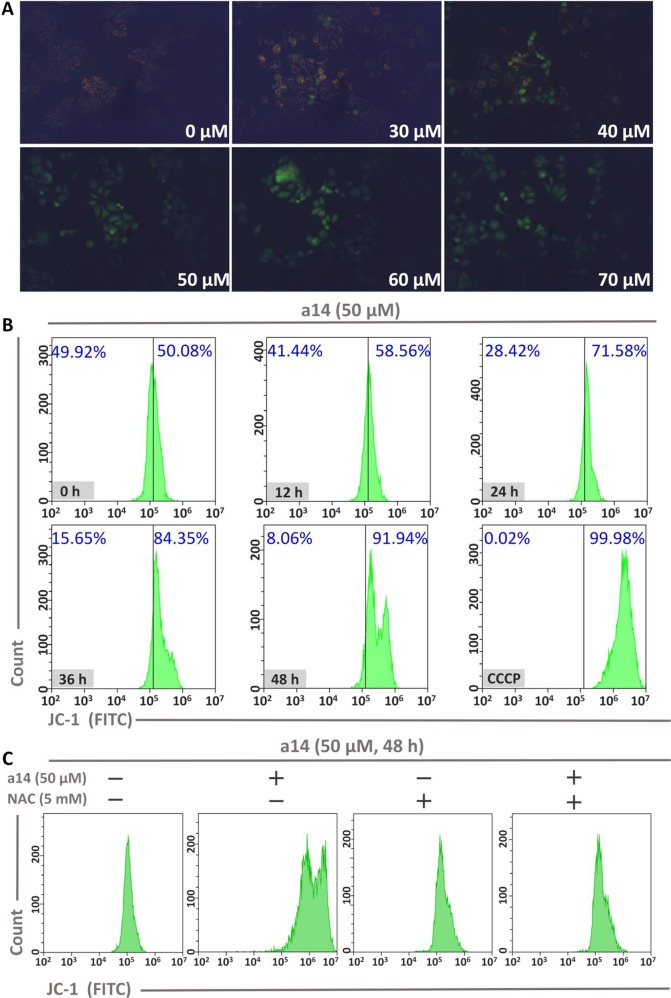
**(A)** The relationship between MMP and **a14** concentration in HepG2 cells; **(B)** The relationship between MMP and action time of **a14** in HepG2 cells (CCCP, as the positive control, was used to completely deprive the MMP); **(C)** Flow cytometric analysis was applied to evaluate the effects on the MMP of **a14** (50 µM) for 48 h with or without the intervention of NAC (5 mM).

To investigate the role of mitochondria in the process of HepG2 cells apoptosis induced by **a14**, the changes in mitochondrial membrane potential (MMP) were measured using JC-1 as the probe. In general, JC-1 shows red fluorescence in normal cells. However, JC-1 will present a monomeric state and show green fluorescence when early apoptosis occurs in cells and the MMP is reduced. In this study, HepG2 cells were incubated with different concentrations of **a14** for 48 h and the pictures were taken by fluorescence microscopy (excitation 480/30× nm, MOTIC CHINA GROUP CO., LTD.). It could be found that almost all the cells showed red fluorescence at the concentration of 0 µM. As the concentration of the **a14** increased, more and more cells emitted green fluorescence (**Figure 4**). When the HepG2 cells were cultured at the concentration of 50 µM, almost all cells had shown green fluorescence, indicating that early-apoptosis occurred among the majority of HepG2 cells.

In addition, time-dependent relationship between fluorescence intensity and the action time of **a14** (50 µM) in HepG2 cells was analyzed by flow cytometry using JC-1 as the probe. Based on the experimental results (**Figure 4**), the intracellular green fluorescence intensity gradually increased by a time-dependent manner. Compared to the group untreated with compound **a14** (50.08%), the intracellular green fluorescence intensity of the HepG2 cells cultured with **a14** (50 µM) for 48 h increased to 91.94%, which was also comparable to HepG2 cells treated by CCCP for 20 min that could completely deprive the MMP (99.98%). To further investigate whether the decrease in MMP of HepG2 cells induced by **a14** was related to ROS, NAC was applied to completely block the production of ROS induced by **a14**. HepG2 cells were pretreated with NAC for 1 h and then co-incubated with **a14** (50 µM) for 48 h. According to **Figure 4**, it could be found that the MMP of HepG2 cells previously treated with NAC could not be decreased by the treatment of **a14** (50 µM), which demonstrated that the intracellular ROS levels would affect the MMP.

### Regulation of Bcl-2 Family Proteins and Caspase 3 by Compound A14

**Figure 5:**
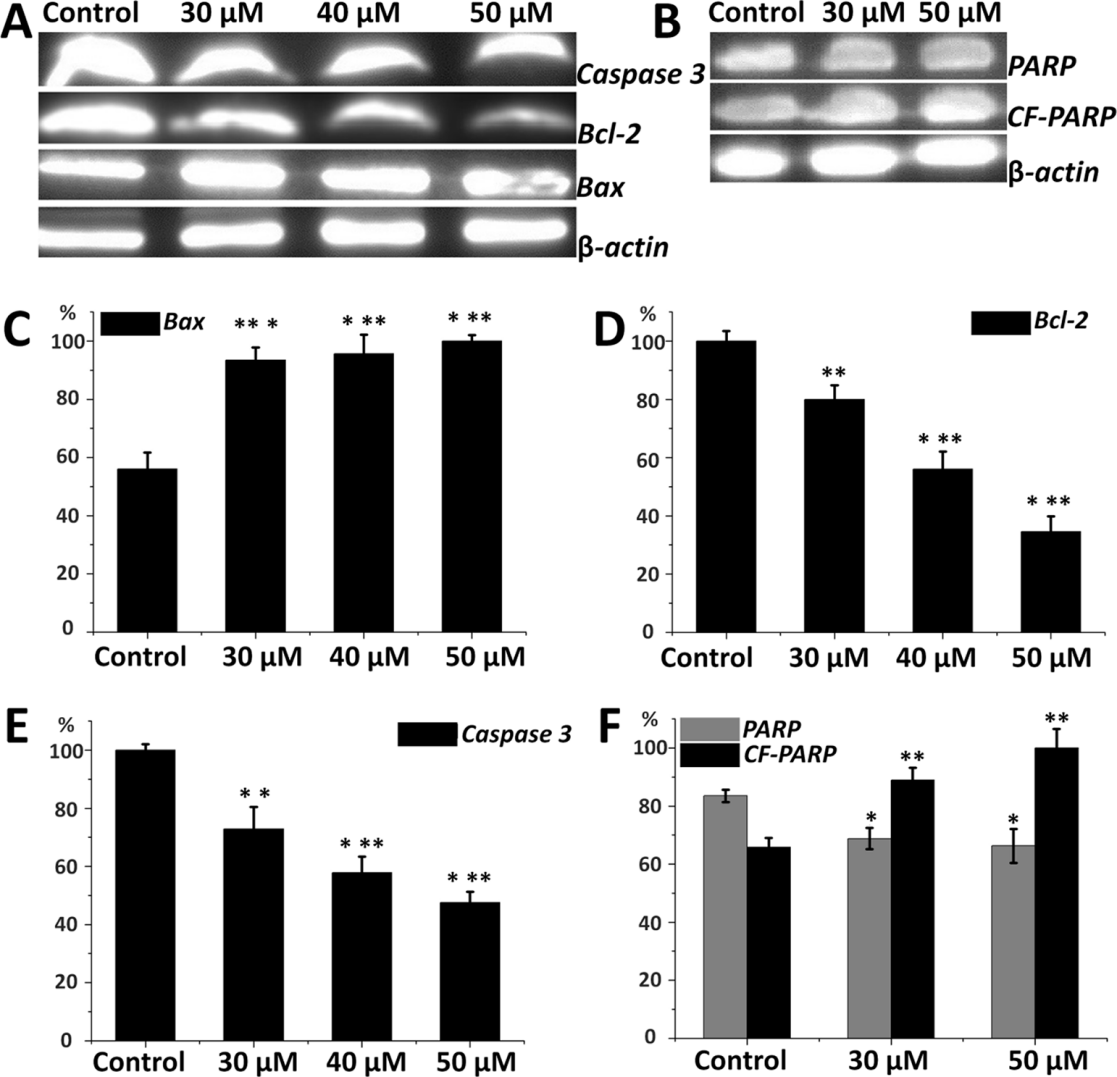
**(A)** Protein levels of Caspase 3, Bax, Bcl-2 in HepG2 cells; **(B)** Protein levels of PARP and CF-PARP proteins in HepG2 cells; **(C)** The relative protein level of Bax; **(D)** The relative protein level of Bcl-2; **(E)** The relative protein level of Caspase 3; **(F)** The relative protein level of PARP and CF-PARP. Values are expressed as the means ± SE, by *t* test, n = 3, *P< 0.05, **P< 0.01, ***P< 0.001 compared with the control.

Expression of apoptosis-related proteins in HepG2 cells is important to elucidate the inhibitory mechanism of compound **a14**. From **Figure 5**, Western blot analyses revealed that HepG2 cells treated with different concentrations of **a14** (48 h) up-regulated the expression of pro-apoptotic proteins (Bax) and correspondingly down-regulated the expression of anti-apoptotic proteins (Bcl-2) by a dose-dependent manner. The results showed **a14** could induce the decrease in the expression level of Caspases 3 and PARP as well with the increase level of cleaved PARP proteins compared to the control group. Therefore, **a14** could not only regulate mitochondria-associated apoptotic proteins (Bax and Bcl-2), but also participate in the regulation of the Caspases 3 related apoptotic protein.

## Discussion

Nowadays, systematic therapies with anti-tumor agents are usually the fundamental treatment strategies for cancer patients, especially the patients with advanced or metastatic tumors (Miyata and Sakai, 2018). However, frequent occurrence of adverse effects during the treatment is a major obstacle to the clinical application of chemotherapy, especially in the elderly patients (Bosch et al., 2004; Itatani et al., 2018; Le Saux and Falandry, 2018; Zhang et al., 2018). Therefore, the development of more effective and safer antitumor agents has become the focus of drug discovery. In general, natural products are advantageous due to the easy availability and relative safety, and chalcone derivatives have good cytotoxic activity towards several tumor cells(Tsai et al., 2014; Yan et al., 2016; Makhdoumi et al., 2017; Wang et al., 2018; Jung et al., 2019), which has led to the increasing consideration of chalcone derivatives as the anti-tumor treatment (Bommareddy et al., 2018; Kim and Kim, 2018). Based on the skeleton of chalcone, the hydrogens on the benzene ring were replaced by various substituents (electron-acceptor groups: –Br, –Cl, and –F; electron-donor groups: –OCH_3_, –CH_3_) to explore the effects of electronegativity on the inhibitory activities of the compounds against the tumor cells. All the synthesized chalcone derivatives were fully characterized by HRMS, ^1^H-NMR, and ^13^C-NMR.

Then, the MTT assay was adopted to evaluate the cytotoxic effects of the target compounds on the tumor cells and human normal cells, and the experimental results showed that the 14 synthesized compounds exerted cytotoxic effect on the adherent tumor cell lines (HepG2, A549, and Hela), indicating that the title compounds had selective inhibition against the tumor cell types and provide guiding value for the selection of cell model in future studies. In addition, from the perspective of structure-activity relationship, it could be found that the IC_50_ of compounds (**a1**, **a2**, **a3**, and **a4**) substituted by electron withdrawing groups was generally larger than that of compounds (**a11**, **a12**, **a13**, and **a14**) substituted by electron donating groups with IC_50_ ranging from 23.30 to 54.37 µM. Therefore, it could be speculated that the electron-donating group (methoxy group) might contribute to the increase in the biological activity of the chalcone derivatives. In the subsequent experiments, further modification of chalcone skeleton was needed to discover more active chalcone derivatives against tumor cells.

Due to the highest cytotoxic activity against HepG2 cells and lowest toxicity to normal cell line WI-38 of **a14**, **a14** was selected as the probe for subsequent studies. According to the experimental results (**Figure 1**), it could be learned that when the action time of **a14** on HepG2 cells was less than 24 h, the cytotoxic effect was relatively weak. However, when the action time was extended to 48 or 72 h, the growth inhibition effect of **a14** on HepG2 cells was significantly improved, indicating that the effect of the drug has certain requirements on the action time.

Cell apoptosis is one of the most prominent ways to regulate cancer cell death, and in the original stages of the apoptotic process, the apoptotic signaling is activated, which will induce the death of tumor cells rather than kill the cells directly. In this study, HepG2 cells were dealt with various concentrations of **a14** for 48 h and the morphological changes of the nucleus could be detected by DAPI staining. According to **Figure 2**, cell morphology underwent a series of significant changes from the qualitative analysis of DAPI staining, such as membrane blebbing, cell shrinkage and detachment, and nuclear condensation and fragmentation. Additionally, the quantitative analysis of Alexa Fluor 647/PI double-staining experiments also proved that **a14** could induce the apoptosis of HepG2 cells. Based on the cycle arrest experiment, **a14** could inhibit the proliferation of HepG2 cells significantly at the phase of G2/M. Compared with the untreated group, the percentages of early apoptosis, late apoptosis, and G2/M rapidly increased by 28.35, 13.51, and 73.82%, when the HepG2 cells were treated with 50 µM **a14** for 48 h. Therefore, the experimental results could serve as a reference for the setting of subsequent experimental parameters *in vivo*.

ROS, a cellular metabolite that regulates multiple cancer-related signaling pathways and can serve as an important regulatory signal for tumor cell apoptosis, has attracted the special attention of researchers (Ralph et al., 2010). Indeed, high levels of ROS can lead to DNA damage and tumor cell apoptosis *via* oxidizing and nitrating macromolecules including RNA, DNA, lipids, and proteins (O'Donovan et al., 2005; Waszczak et al., 2014). In this research, **a14** could induce a large amount of ROS production in HepG2 cells in a short period of time, which could be blocked by antioxidant NAC (**Figure 3**). Moreover, the blocking effects of NAC could weaken the pro-apoptotic effects of compound **a14** on HepG2 cells (**Figure 3**), indicating that the pro-apoptotic mechanism of compound **a14** was closely related to the ROS pathway.

Moreover, mitochondria play an important role in the apoptotic pathway of tumor cells, and the mitochondria-dependent apoptotic pathway could be regulated by pro-apoptotic and anti-apoptotic proteins of the Bcl-2 and caspase families (Guerra et al., 2011; Wang et al., 2013). According to **Figure 4**, the MMP of HepG2 cells could decrease under the induction of **a14**, and the decrease in MMP could be blocked by NAC intervention, which indicated that the decrease in MMP was related to the expression level of ROS. In addition, the expression levels of mitochondria-related apoptosis proteins (Bax, Bcl-2, and Caspase 3) also changed significantly by the intervention of **a14**. In summary, compound **a14** could block HepG2 cells from mitosis in the G2/M phase and induce early apoptosis and late apoptosis in HepG2 cells, and the apoptosis mechanism induced by **a14** was related to the ROS-mitochondrial pathway (expression level of ROS, MMP, and the apoptosis-related proteins) through a series of biological experiments. In the follow-up study, anti-tumor effects *in vivo* will also be evaluated, which aims to provide valuable candidate compounds for the research of anti-tumor drugs.

## Data Availability Statement

All datasets generated for this study are included in the article/**Supplementary Material**.

## Author Contributions

DH and LZ conceived the work and directed the experiments; HZ and LT performed the experiments and collected the data. CZ, PY, and BW analyzed the data. HZ and YZ drafted the first and second versions of the manuscript. All authors read, edited, and approved the final version of the manuscript.

## Funding

This work was supported by Lanzhou Science and Technology Bureau Program Funds (2016-3-108, 2017-RC-16), Gansu Science and Technology Fund Grant (ID: 17ZD2FA009, 18JR3RA417), and National Science and Technology Ministry (ID: 2017ZX09101001).

## Conflict of Interest

HZ, LT, and CZ worked at Lanzhou Weihuan Biological Science and Technology Development Co, Ltd and studied at the School of Pharmacy of Lanzhou University. In the process of completing the project, the company provided the experimental apparatus.

The remaining authors declare that the research was conducted in the absence of any commercial or financial relationships that could be construed as a potential conflict of interest.

## Abbreviations

MMP, mitochondrial membrane potential; DMEM, Dulbecco’s modified Eagle medium; FBS, fetal bovine serum; MTT, Thiazolyl Blue Tetrazolium Bromid; DAPI, 4′, 6-diamidino-2-phenylindole; ROS, reactive oxygen species; NAC, N-acetyl-L-cysteine; DCFH-DA, 2, 7-Dichlorodi-hydrofluorescein diacetate; CCCP, carbonyl cyanide m-chlorophenyl hydrazine; Bcl-2, B-cell lymphoma-2.
