# Supplementary material for: Synthesis of Chalcone Derivatives: Inducing Apoptosis of HepG2 Cells via Regulating Reactive Oxygen Species and Mitochondrial Pathway
Source: Front Pharmacol. 2019 Nov 15;10:1341. doi: 10.3389/fphar.2019.01341 (PMC6874057; doi:10.3389/fphar.2019.01341)
Supplement: Supplementary file 1 [file DataSheet_1.pdf]

Synthesis of Chalcone Derivatives: Inducing Apoptosis of HepG2 Cells via Regulating ROS and Mitochondrial Pathway

Hongtian Zhu [1, 3], Lei Tang [1, 3], Chenghong Zhang [1, 3], Baochu Wei [1, 2], Pingrong Yang [1, 4], Dian He [1, 4*], Lifang Zheng [1*], Yang Zhang [3, 5*]

[1] Materia Medica Development Group, Institute of Medicinal Chemistry, Lanzhou University School of Pharmacy, Lanzhou 730000, China

[2] Lanzhou Pulmonary Hospital Pharmacy, Lanzhou 730046, China

[3] Lanzhou Weihuan Biological Science and Technology Development Co, Ltd. Lanzhou 730000, China

[4] Gansu Institute for Drug Control, Lanzhou 730070, China

[5] School of Pharmaceutical Sciences, Chongqing University, Chongqing 401331, China

* Corresponding authors:
Hed@lzu.edu.cn (D. He);
zhangy2016@cqu.edu.cn (Y. Zhang);
zhenglf@lzu.edu.cn (L. Zheng)

Contents

1. Compounds spectrum analysis....2

2. ^{1}H-NMR information of the synthesized compounds....6

3. ^{13}C-NMR information of the synthesized compounds....20

4. HRMS information of the synthesized compounds....34

5. The purity information of the synthesized compounds.... 41

1. Compounds spectrum analysis

*1.1. (E)-5-(3-(4-fluorophenyl)acryloyl)-2-hydroxybenzamide (**a1**)*

White solid, m.p. 222.2-222.7 °C. Yield 83%; ^{1}H NMR (400 MHz, DMSO-*d6*): δ 13.98 (s, 1H), 8.80 (s, 1H), 8.71 (s, 1H), 8.23 (d, *J* = 8.8 Hz, 2H), 7.99 (t, 2H), 7.94 (d, *J* = 15.5 Hz, 1H), 7.77 (d, *J* = 15.5 Hz, 1H), 7.36 (t, 2H), 7.07 (d, *J* = 8.6 Hz, 1H); ^{13}C NMR (100 MHz, DMSO-*d6*): δ 186.97, 172.28, 165.84, 162.43, 142.60 , 134.82, 131.97, 131.52, 130.10, 128.98, 122.08, 118.64, 116.51, 116.29, 114.30; HRMS (ESI): found 286.0890 for $[M+H]^+$ (calcd. for $C_{16}H_{12}FNO_3$, 285.0801). Purity: 99.0% (by HPLC).

*1.2. (E)-5-(3-(3-chlorophenyl)acryloyl)-2-hydroxybenzamide (**a2**)*

White solid, m.p. 212.3-212.8 °C. Yield 76%; ^{1}H NMR (400 MHz, DMSO-*d6*): δ 14.01 (s, 1H), 8.80 (s, 1H), 8.72 (s, 1H), 8.24 (d, *J* = 8.0 Hz, 2H), 8.07 (d, 2H), 7.83 (s, 1H), 7.74 (d, *J* = 15.6 Hz, 1H), 7.51 (t, 2H), 7.06 (d, *J* = 8.6 Hz, 1H); ^{13}C NMR (100 MHz, DMSO-*d6*): δ 186.67, 171.78, 165.52, 151.26, 142.13, 137.55, 134.91, 134.28, 131.08, 130.51, 130.17, 128.79, 128.23, 123.71, 118.67, 114.32; HRMS (ESI): found 324.0410 for $[M+Na]^+$ (calcd. for $C_{16}H_{12}ClNO_3$, 301.0506). Purity: 91.4% (by HPLC).

*1.3. (E)-5-(3-(3-bromophenyl)acryloyl)-2-hydroxybenzamide (**a3**)*

Pink solid, m.p. 211.4-211.5 °C. Yield 68%; ^{1}H NMR (400 MHz, DMSO-*d6*): δ 13.99 (s, 1H), 8.79 (s, 1H), 8.71 (s, 1H), 8.25 (d, *J* = 8.8 Hz, 2H), 8.16 (s, 1H), 8.05 (d, *J* = 15.6 Hz, 1H), 7.87 (d, *J* = 8.0 Hz, 1H), 7.74 (d, *J* = 15.2 Hz, 1H), 7.66 (d, *J* = 8.0 Hz, 1H), 7.46 (t, 1H), 7.06 (d, *J* = 8.7 Hz, 1H); ^{13}C NMR (100 MHz, DMSO-*d6*): δ 190.71, 172.46, 170.94, 166.24, 144.33, 136.24, 134.54, 131.90, 130.87, 130.13, 127.02, 126.52, 123.24, 122.63, 117.84, 116.89; HRMS (ESI): found 369.9879 for $[M+Na]^+$ (calcd. for $C_{16}H_{12}BrNO_3$, 345.0001). Purity: 95.1% (by HPLC).

*1.4. (E)-5-(3-(4-bromophenyl)acryloyl)-2-hydroxybenzamide (**a4**)*

Yellow solid, m.p. 226.9-227.5 °C. Yield 66%; ^{1}H NMR (400 MHz, DMSO-*d6*): δ 13.99 (s, 1H), 8.79 (s, 1H), 8.70 (s, 1H), 8.22 (d, *J* = 8.8 Hz, 2H), 8.01 (d, *J* = 15.6 Hz, 1H), 7.86 (d, *J* = 8.4 Hz, 2H), 7.73 (d, *J* = 15.8 Hz, 1H), 7.69 (d, *J* = 8.5 Hz, 2H), 7.06 (d, *J* = 8.7 Hz, 1H); ^{13}C NMR (100 MHz, DMSO-*d6*): δ 187.09, 172.33, 165.90, 142.48, 134.87, 134.59, 132.37, 131.11, 130.16, 128.90, 124.32, 122.97, 118.69, 114.32; HRMS (ESI): found 346.0056 for $[M+H]^+$ (calcd. for $C_{16}H_{12}BrNO_3$, 345.0001). Purity: 94.2% (by HPLC).

*1.5. (E)-5-cinnamoyl-2-hydroxybenzamide (**a5**)*

Yellow solid, m.p. 182.7-184.5 °C. Yield 74%; ^{1}H NMR (400 MHz, DMSO-*d6*): δ 14.01 (s, 1H), 8.82 (s, 1H), 8.72 (s, 1H), 8.23 (d, *J* = 8.8 Hz, 2H), 8.00 (d, *J* = 15.5 Hz, 1H), 7.89 (m, 2H), 7.78 (d, *J* = 15.5 Hz, 1H), 7.50 (m, 3H), 7.08 (d, *J* = 8.7 Hz, 1H); ^{13}C NMR (100 MHz, DMSO-*d6*): δ 187.11 , 172.06, 165.95, 135.29, 134.84, 130.99, 130.14, 129.31, 129.00, 122.19, 118.70, 118.19, 114.27; HRMS (ESI): found 268.0983 for $[M+H]^+$ (calcd. for $C_{16}H_{13}NO_3$, 267.0895). Purity: 95.9% (by HPLC).

*1.6. (E)-2-hydroxy-5-(3-(o-tolyl)acryloyl)benzamide (**a6**)*

Yellow solid, m.p. 163.1-164.9 °C. Yield 68%; ^{1}H NMR (400 MHz, DMSO-*d6*): δ 14.02 (s, 1H), 8.83 (s, 1H), 8.72 (s, 1H), 8.21 (d, *J* = 9.0 Hz, 2H), 8.04 (d, *J* = 15.6 Hz, 1H)), 7.99 (d, *J* = 8.2 Hz, 1H), 7.89 (d, *J* = 15.4 Hz, 1H), 7.41 – 7.23 (m, 3H), 7.07 (d, *J* = 8.7 Hz, 1H), 2.44 (d, *J* = 12.4 Hz, 3H); ^{13}C NMR (100 MHz, DMSO-*d6*): δ 187.14 , 172.18, 165.86, 140.99, 138.39, 134.82, 133.97, 131.27, 130.64, 130.09, 129.00, 127.20, 126.69, 123.12, 118.76, 114.20, 38.70 – 38.06; HRMS (ESI): found 282.1193 for $[M+H]^+$ (calcd. for $C_{17}H_{15}NO_3$, 281.1052). Purity: 99.1% (by HPLC).

*1.7. (E)-2-hydroxy-5-(3-(m-tolyl)acryloyl)benzamide (**a7**)*

White solid, m.p. 171.4-171.5 °C. Yield 62%; ^{1}H NMR (400 MHz, DMSO-*d6*): δ 14.00 (s, 1H), 8.83 (s, 1H), 8.71 (s, 1H), 8.20 (d, *J* = 8.9 Hz, 2H), 7.95 (d, *J* = 15.5 Hz, 1H), 7.72 (d, *J* = 15.5 Hz, 1H), 7.68 – 7.59 (m, 2H), 7.34 (t, *J* = 7.5 Hz, 1H), 7.25 (d, *J* = 7.4 Hz, 1H), 7.04 (d, *J* = 8.7 Hz, 1H), 2.36 (s, 3H); ^{13}C NMR (100 MHz, DMSO-*d6*): δ 187.16, 165.93, 143.90, 138.46, 135.22, 134.81, 131.69, 130.06, 129.55, 129.16, 128.90, 126.60, 121.89, 118.66, 114.24, 21.31; HRMS (ESI): found 282.1191 for $[M+H]^+$ (calcd. for $C_{17}H_{15}NO_3$, 281.1052). Purity: 99.8% (by HPLC).

*1.8. (E)-2-hydroxy-5-(3-(p-tolyl)acryloyl)benzamide (**a8**)*

Yellow solid, m.p. 190.5-191.7 °C. Yield 71%; ^{1}H NMR (400 MHz, DMSO-*d6*): δ 13.63 (s, 1H), 8.77 (s, 1H), 8.69 (s, 1H), 8.32 – 8.02 (m, 2H), 8.01 (d, *J* = 15.5 Hz, 1H), 7.85 – 7.65 (m, 2H), 7.49 – 7.12 (m, 3H), 7.03 (d, *J* = 8.7 Hz, 1H), 2.33 (s, 3H); ^{13}C NMR (100 MHz, DMSO-*d6*): δ 187.05, 172.33, 165.94, 143.86, 140.93, 134.73, 132.51, 130.02, 129.10, 127.01, 121.01, 118.66, 114.15, 20.99; HRMS (ESI): found 282.1228 for $[M+H]^+$ (calcd. for $C_{17}H_{15}NO_3$, 281.1052). Purity: 91.5% (by HPLC).

*1.9. (E)-2-hydroxy-5-(3-(2-methoxyphenyl)acryloyl)benzamide (**a9**)*

Yellow solid, m.p. 237.2-238.9 ℃. Yield 71%; ^{1}H NMR (400 MHz, DMSO-*d6)*: δ 13.97 (s, 1H), 8.81 (s, 1H), 8.70 (s, 1H), 8.20 (d, *J* = 8.8 Hz, 2H), 8.11 (d, *J* = 15.6 Hz, 1H), 7.99 (d, *J* = 7.6 Hz, 1H), 7.93 (d, *J* = 15.7 Hz, 1H), 7.47 (t, *J* = 7.3 Hz, 1H), 7.13 (d, *J* = 8.2 Hz, 1H), 7.09 – 7.05 (t, *J* = 7.7 Hz, 2H), 3.91 (s, 3H); ^{13}C NMR (100 MHz, DMSO-*d6*): δ 190.71, 170.94, 166.24, 157.37, 140.86, 134.54, 130.87, 130.57, 129.59, 127.84, 126.52, 121.88, 120.84, 117.84, 116.89, 113.50, 56.79; HRMS (ESI): found 298.1220 for $[M+H]^+$ (calcd. for $C_{17}H_{15}NO_4$, 297.1001). Purity: 97.7% (by HPLC).

*1.10. (E)-2-hydroxy-5-(3-(3-methoxyphenyl)acryloyl)benzamide (**a10**)*

Yellow solid, m.p. 194.5-195.2 °C. Yield 77%; ^{1}H NMR (400 MHz, DMSO-*d6*): δ 13.98 (s, 1H), 8.80 (s, 1H), 8.71 (s, 1H), 8.23 (d, *J* = 8.7 Hz, 2H), 7.98 (d, *J* = 15.5 Hz, 1H), 7.74 (d, *J* = 15.5 Hz, 1H), 7.46 (s, 2H), 7.40 (t, *J* = 8.0 Hz, 1H), 7.06 (d, *J* = 8.7 Hz, 2H), 3.84 (s, 3H); ^{13}C NMR (100 MHz, DMSO-*d6*): δ 187.15, 172.20, 165.97, 160.04, 143.87, 136.49, 134.76, 130.27, 129.94, 128.83, 122.38, 121.68, 118.52, 116.50, 114.22, 55.60; HRMS (ESI): found 298.1179 for $[M+H]^+$ (calcd. for $C_{17}H_{15}NO_4$, 297.1001). Purity: 98.9% (by HPLC).

*1.11. (E)-2-hydroxy-5-(3-(4-methoxyphenyl)acryloyl)benzamide (**a11**)*

Yellow solid, m.p. 212.8-212.8 °C. Yield 59%; ^{1}H NMR (400 MHz, DMSO-d6): δ 13.95 (s, 1H), 8.80 (s, 1H), 8.70 (s, 1H), 8.21 (d, *J* = 8.7 Hz, 2H), 7.93 – 7.81 (m, 3H), 7.74 (d, *J* = 15.4 Hz, 1H), 7.05 (d, *J* = 8.5 Hz, 3H), 3.84 (s, 3H); ^{13}C NMR (100 MHz, DMSO-*d6*): δ 187.04, 172.21, 165.90, 161.50,

143.73, 134.56, 131.02, 129.83, 129.16 – 128.96, 127.70, 119.32, 118.45, 114.58, 114.14, 55.85; HRMS (ESI): found 320.0902 for $[M+Na]^+$ (calcd. for $C_{17}H_{15}NO_4$, 297.1001). Purity: 95.5% (by HPLC).

1.12. (E)-5-(3-(2,4-dimethoxyphenyl)acryloyl)-2-hydroxybenzamide ***(a12)***

Yellow solid, m.p. 193.7-193.7 °C. Yield 76%; ^{1}H NMR (400 MHz, DMSO-*d6*): δ 13.84 (s, 1H), 8.73 (s, 1H), 8.53 (s, 1H), 8.21 – 8.07 (m, 2H), 8.05 (d, *J* = 15.5 Hz, 1H), 7.98 (m, 1H), 7.79 (d, *J* = 15.5 Hz, 1H), 6.98 (d, *J* = 8.5 Hz, 2H), 6.65 (s, 1H), 3.88 (d, *J* = 23.9 Hz, 3H), 2.54 (s, 3H); ^{13}C NMR (100 MHz, DMSO-*d6*): δ 196.13, 187.13, 171.72, 165.66, 163.27, 160.01, 138.00, 134.09, 130.19, 129.61, 128.25 – 128.05, 119.03, 118.50, 117.96, 114.30, 106.85, 98.70, 56.28, 55.88, 26.62; HRMS (ESI): found 328.1198 for $[M+H]^+$ (calcd. for $C_{18}H_{17}NO_5$, 327.1107). Purity: 91.3% (by HPLC).

1.13. *(E)-5-(3-(3,4-dimethoxyphenyl)acryloyl)-2-hydroxybenzamide* ***(a13)***

Yellow solid, m.p. 213.9-214.6 °C. Yield 85%; ^{1}H NMR (400 MHz, DMSO-*d6*): δ 13.91 (s, 1H), 8.78 (s, 1H), 8.70 (s, 1H), 8.31 – 8.13 (m, 2H), 7.85 (d, *J* = 15.4 Hz, 1H), 7.73 (d, *J* = 15.4 Hz, 1H), 7.51 (s, 1H), 7.44 (d, *J* = 8.3 Hz, 1H), 7.06 (d, *J* = 8.7 Hz, 2H), 3.86 (d, *J* = 16.6 Hz, 6H); ^{13}C NMR (101 MHz, DMSO-*d6*): δ 187.22, 186.68, 172.24, 165.65, 151.78 – 151.54, 149.40, 144.56 – 144.18, 134.83 (s), 129.99, 129.32, 128.09, 123.83, 119.87, 118.42, 114.45, 112.11, 111.78, 56.14; HRMS (ESI): found 350.1010 for $[M+Na]^+$ (calcd. for $C_{18}H_{17}NO_5$, 327.1107). Purity: 94.8% (by HPLC).

1.14. *(E)-2-hydroxy-5-(3-(3,4,5-trimethoxyphenyl)acryloyl)benzamide* ***(a14)***

Yellow solid, m.p. 259.9-260.8 °C. Yield 82%; ^{1}H NMR (400 MHz, DMSO-*d6*): δ 13.90 (s, 1H), 8.79 (s, 1H), 8.70 (s, 1H), 8.24 (d, *J* = 8.6 Hz, 1H), 8.17 (s, 1H), 7.89 (d, *J* = 15.5 Hz, 1H), 7.70 (d, *J* = 15.5 Hz, 1H), 7.22 (s, 2H), 7.05 (d, *J* = 8.6 Hz, 1H), 3.87 (s, 6H), 3.72 (s, 3H); ^{13}C NMR (100 MHz, DMSO-*d6*): δ 187.36, 172.20 , 165.75, 153.59, 144.35, 140.23, 134.93, 130.82, 130.17, 129.19, 121.59, 118.36, 114.62, 107.02, 60.63, 56.62; HRMS (ESI): found 358.1356 for $[M+H]^+$ (calcd. for $C_{19}H_{19}NO_6$, 357.1212). Purity: 95.0% (by HPLC).

2. H-NMR information of the synthesized compounds

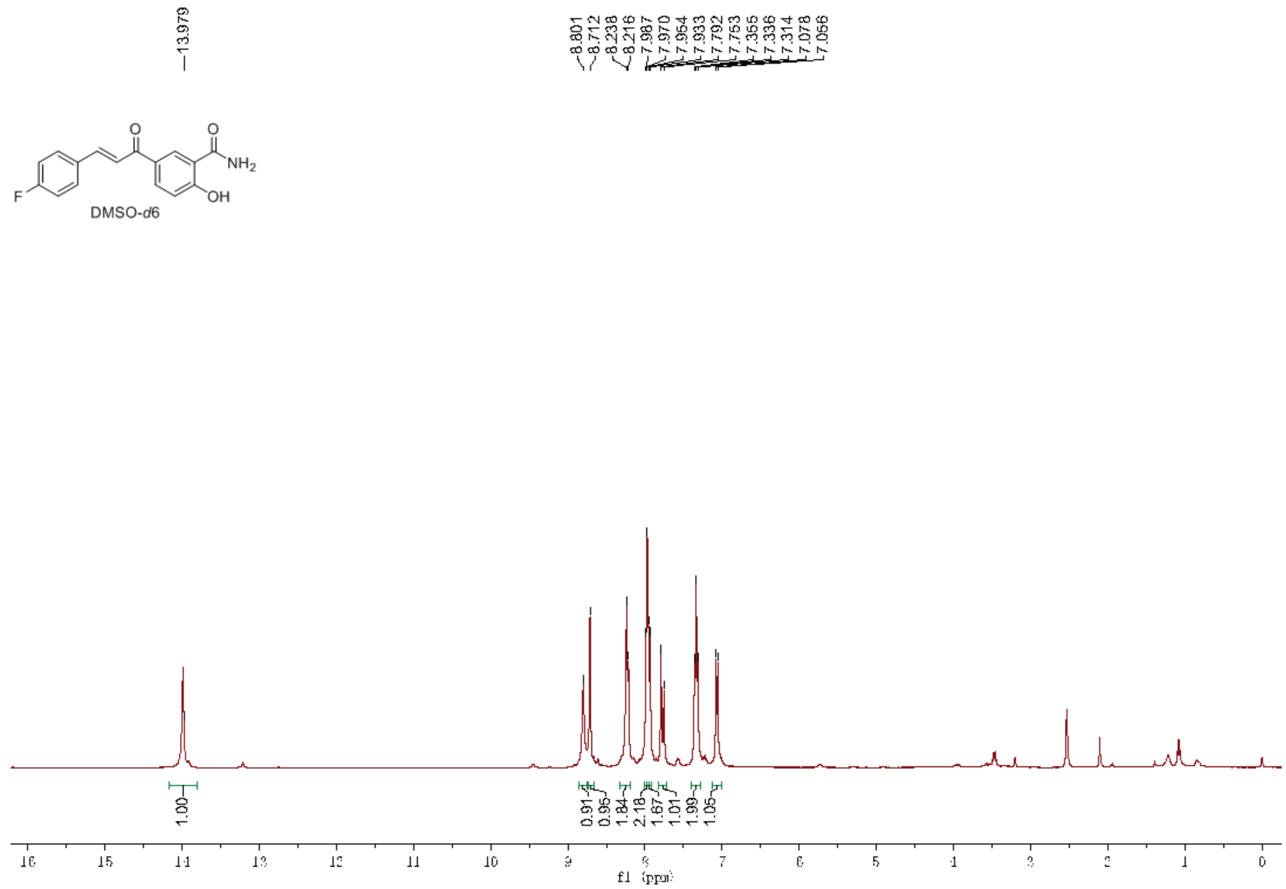

*(E)-5-(3-(4-fluorophenyl)acryloyl)-2-hydroxybenzamide (**a1**)*

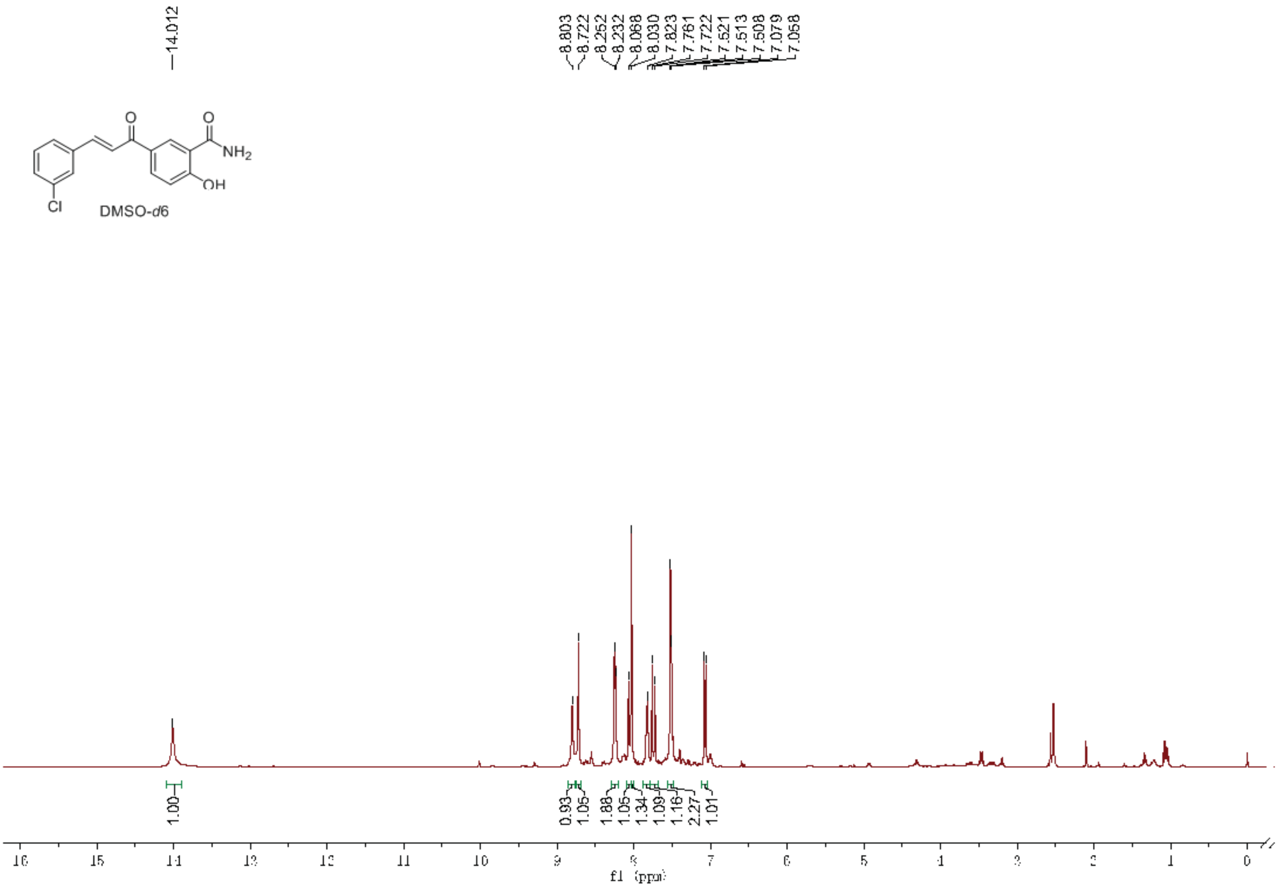

*(E)-5-(3-(3-chlorophenyl)acryloyl)-2-hydroxybenzamide (**a2**)*

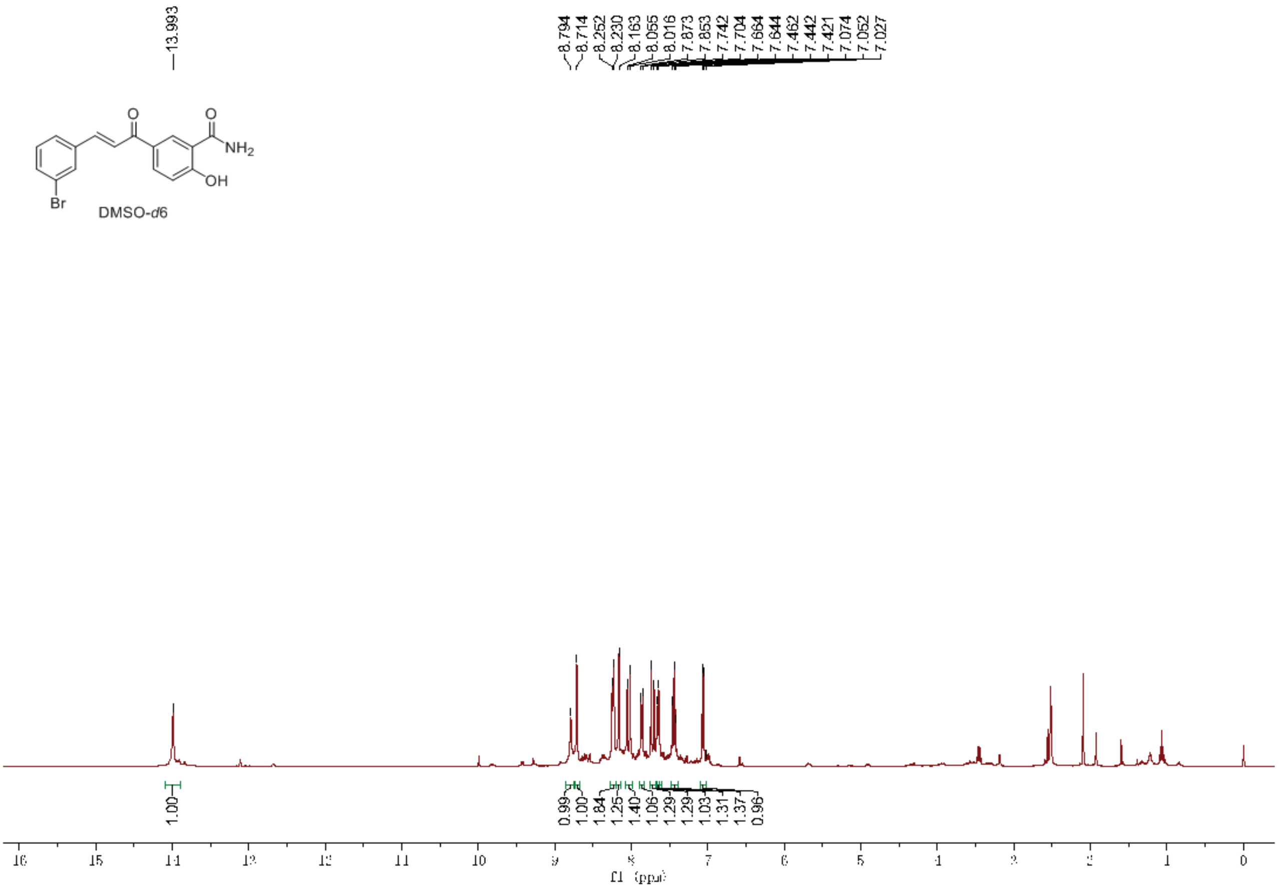

*(E)-5-(3-(3-bromophenyl)acryloyl)-2-hydroxybenzamide (**a3**)*

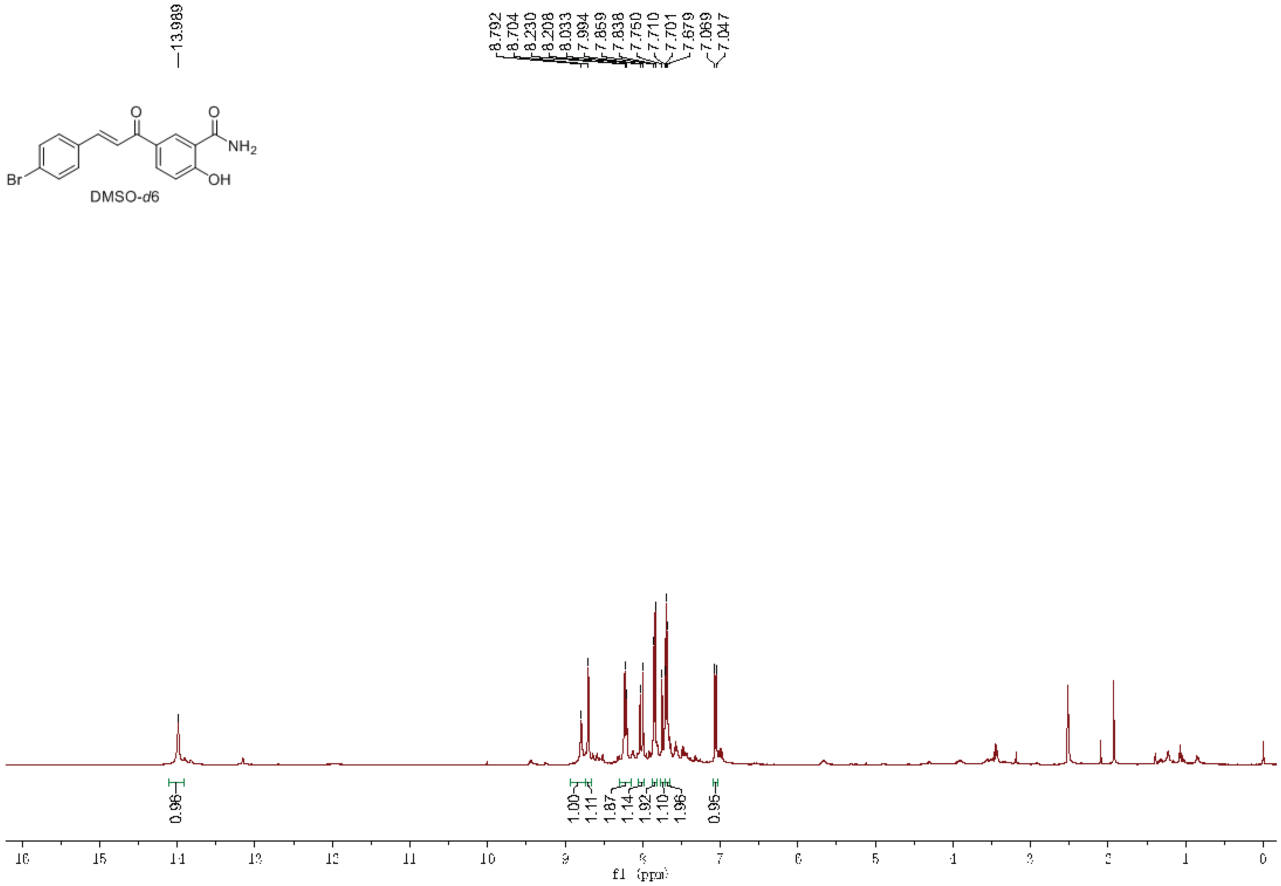

(E)-5-(3-(4-bromophenyl)acryloyl)-2-hydroxybenzamide ***(a4)***

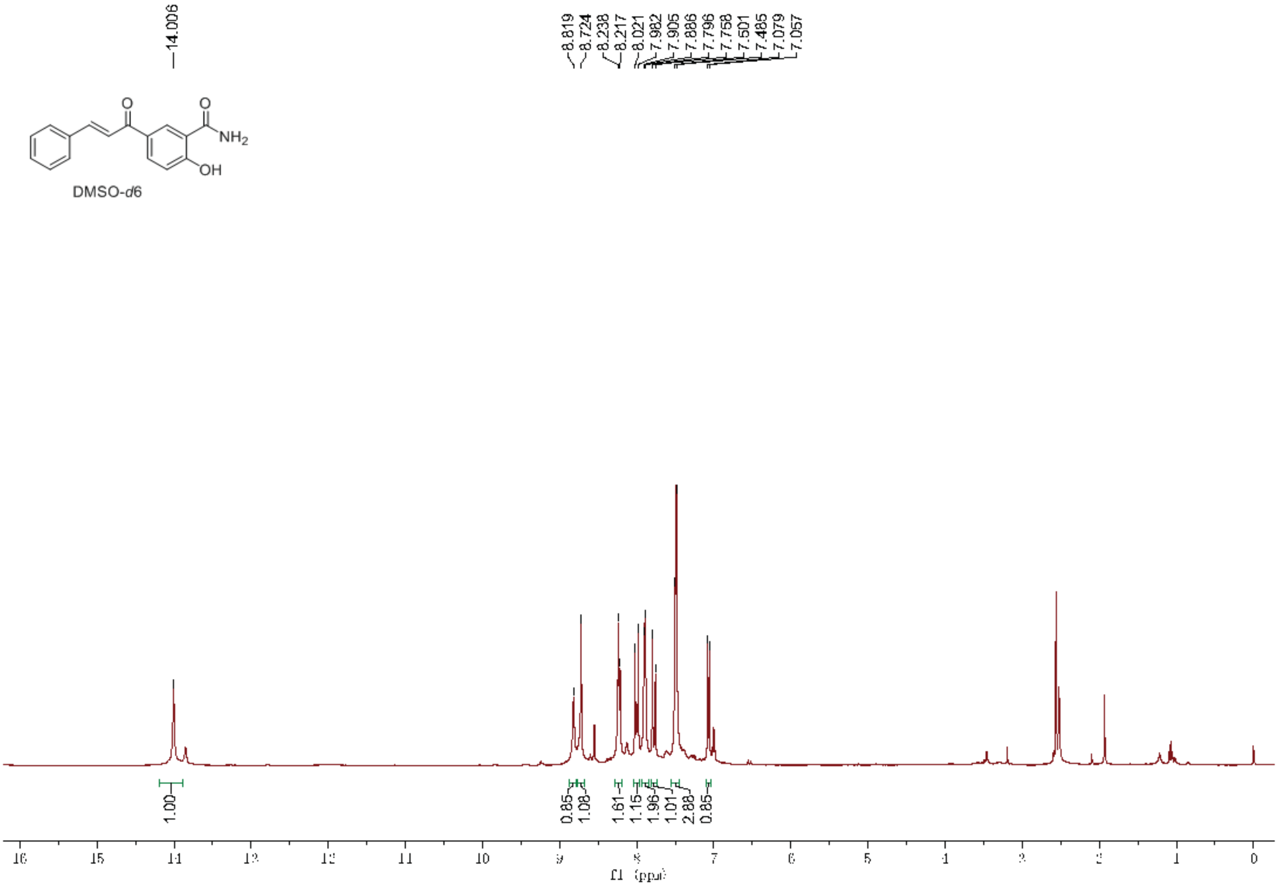

*(E)-5-cinnamoyl-2-hydroxybenzamide (**a5**)*

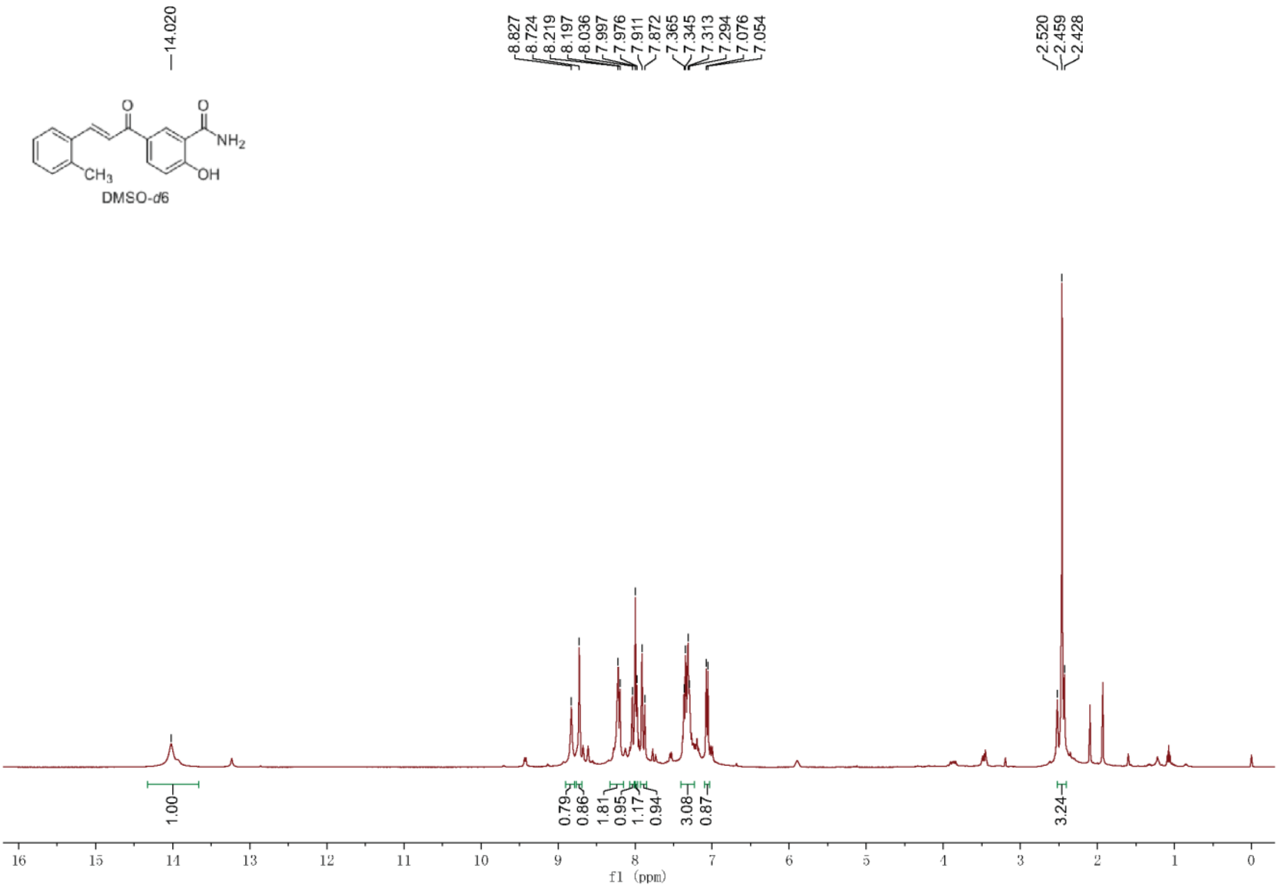

*(E)-2-hydroxy-5-(3-(o-tolyl)acryloyl)benzamide (**a6**)*

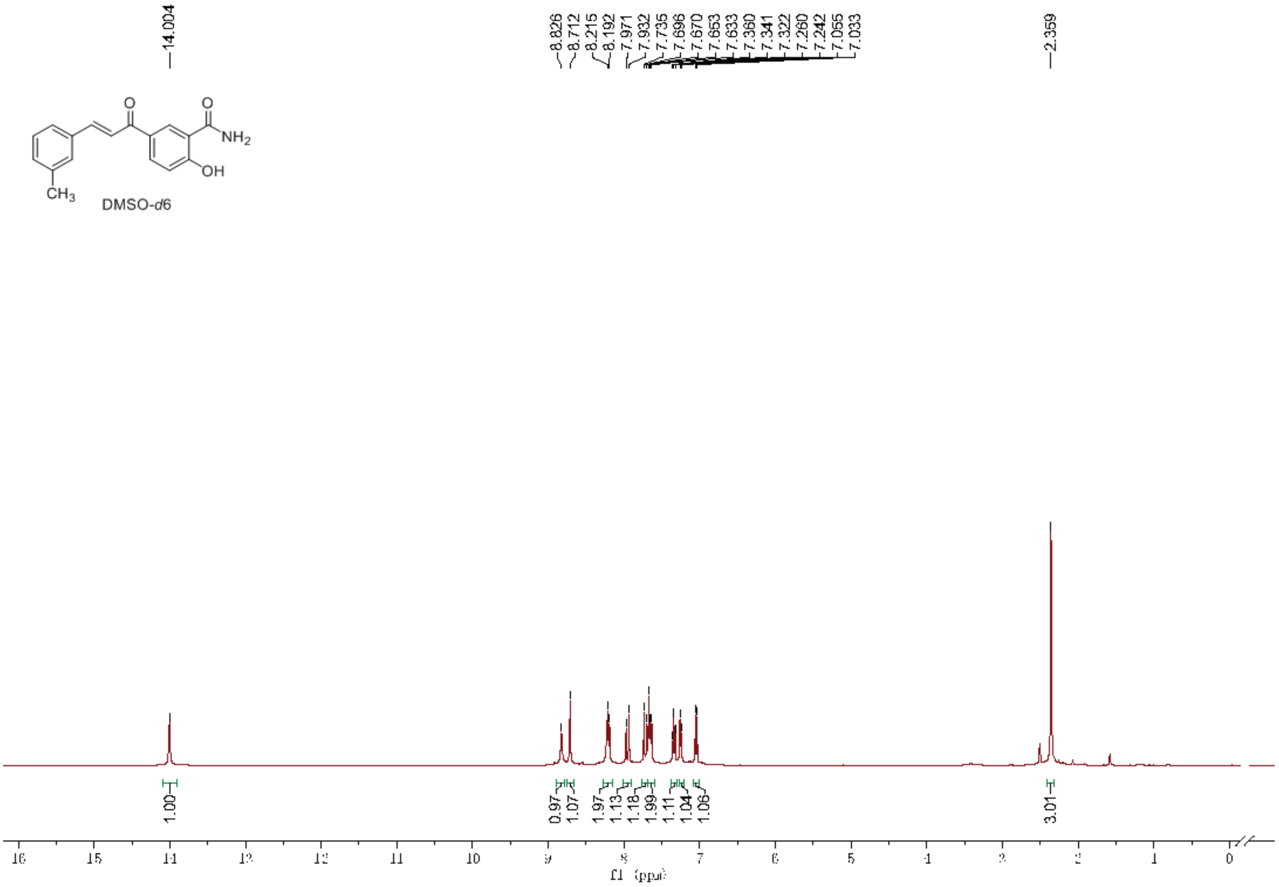

*(E)-2-hydroxy-5-(3-(m-tolyl)acryloyl)benzamide (**a**7)*

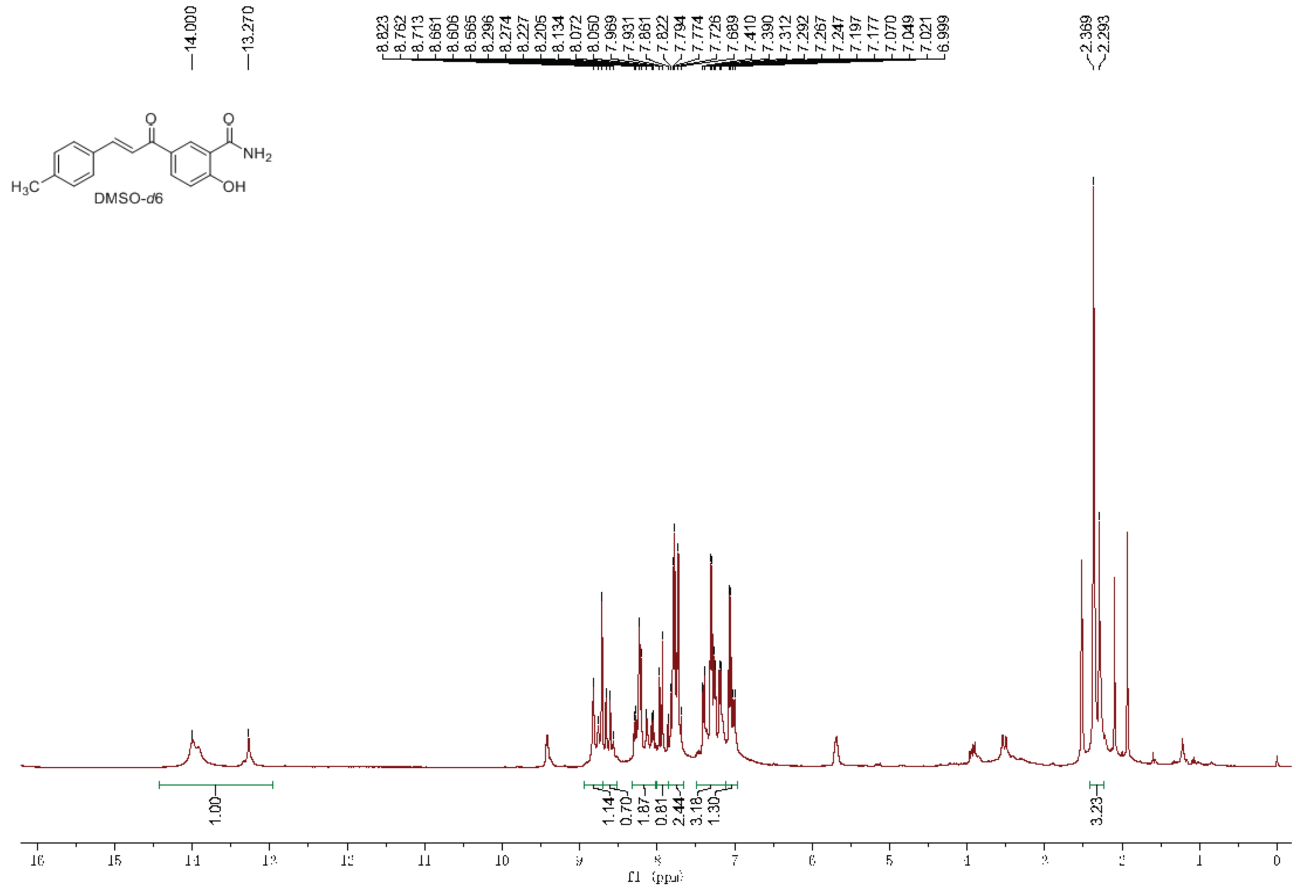

*(E)-2-hydroxy-5-(3-(p-tolyl)acryloyl)benzamide (**a8**)*

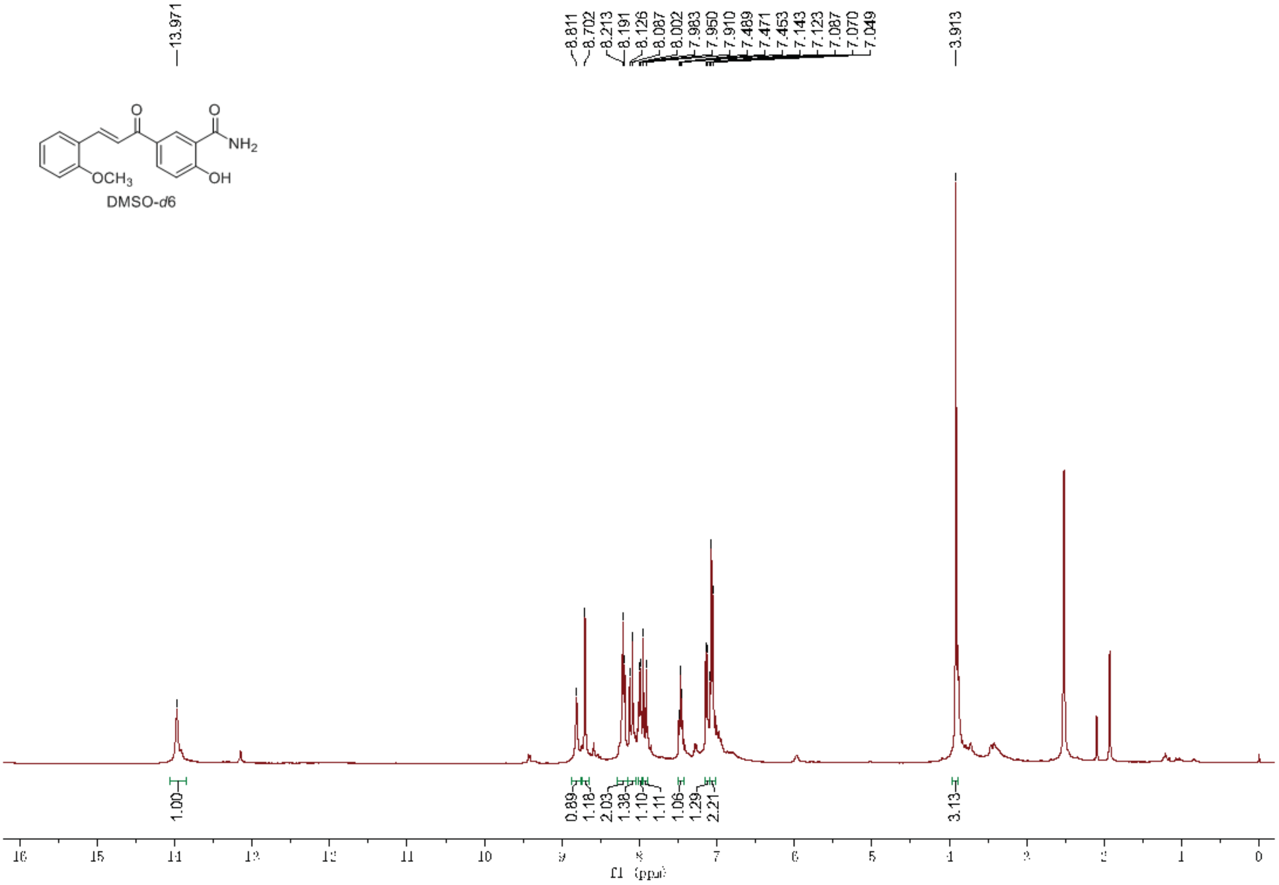

*(E)-2-hydroxy-5-(3-(2-methoxyphenyl)acryloyl)benzamide (**a9**)*

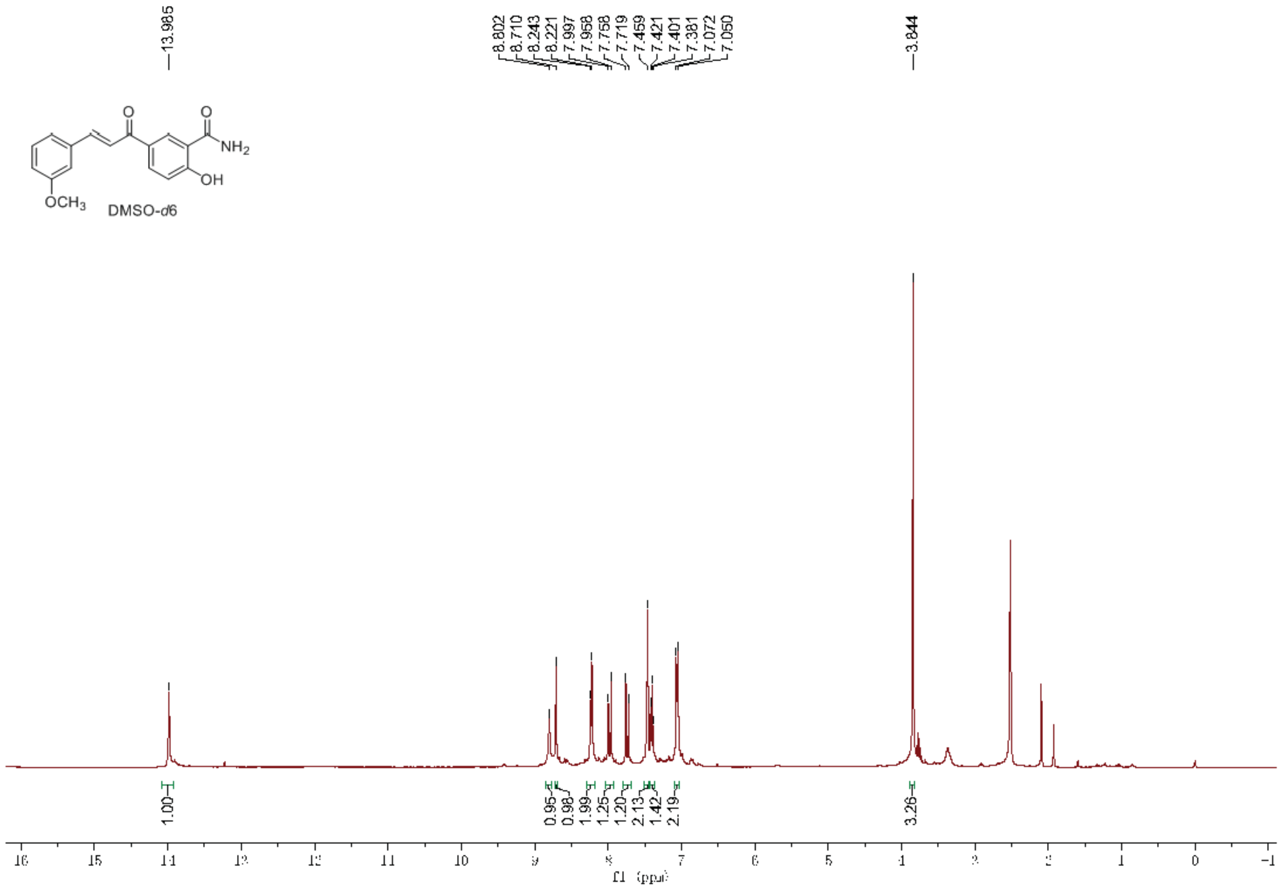

*(E)-2-hydroxy-5-(3-(3-methoxyphenyl)acryloyl)benzamide(**a10**)*

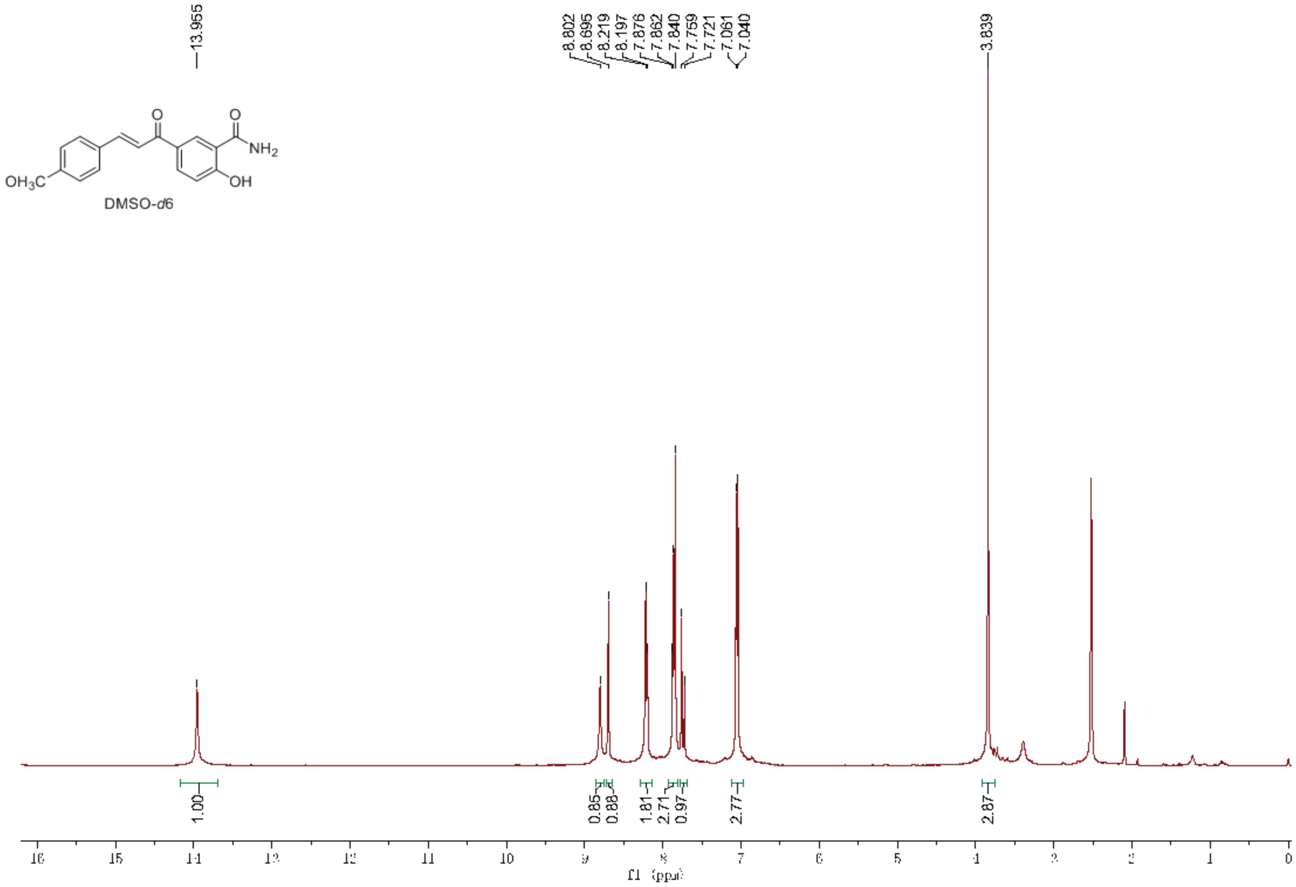

*(E)-2-hydroxy-5-(3-(4-methoxyphenyl)acryloyl)benzamide(**a11**)*

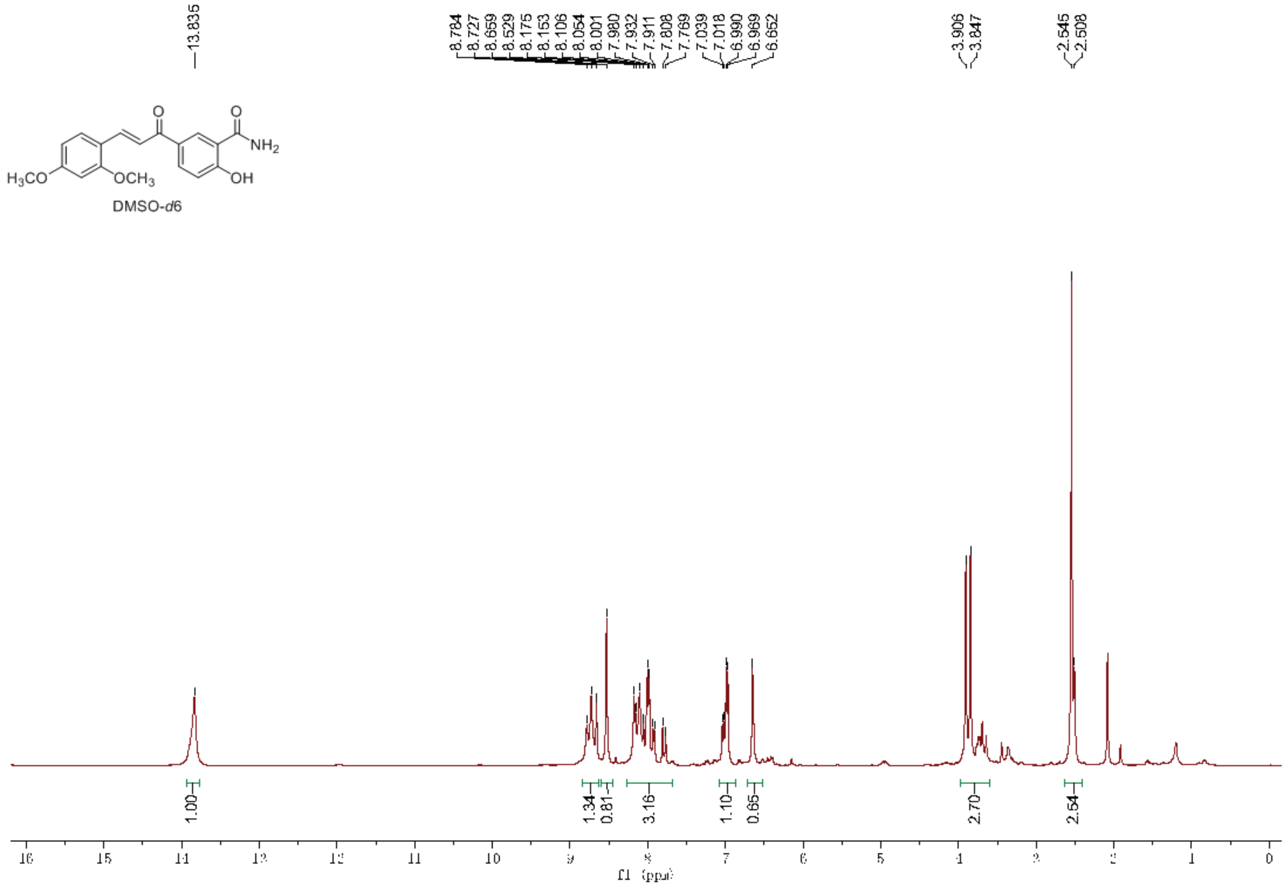

(E)-5-(3-(2,4-dimethoxyphenyl)acryloyl)-2-hydroxybenzamide ***(a12)***

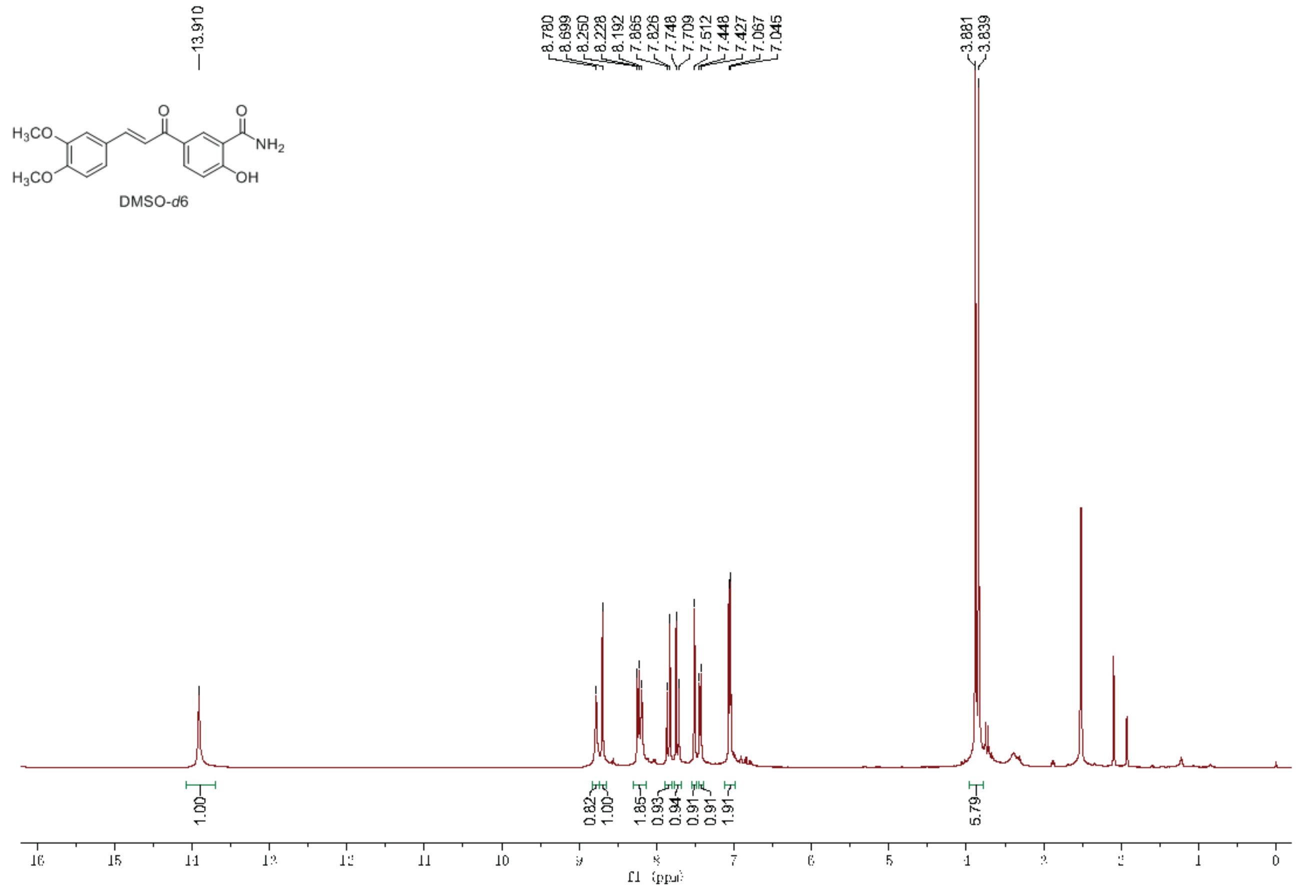

(E)-5-(3-(3,4-dimethoxyphenyl)acryloyl)-2-hydroxybenzamide ***(a13)***

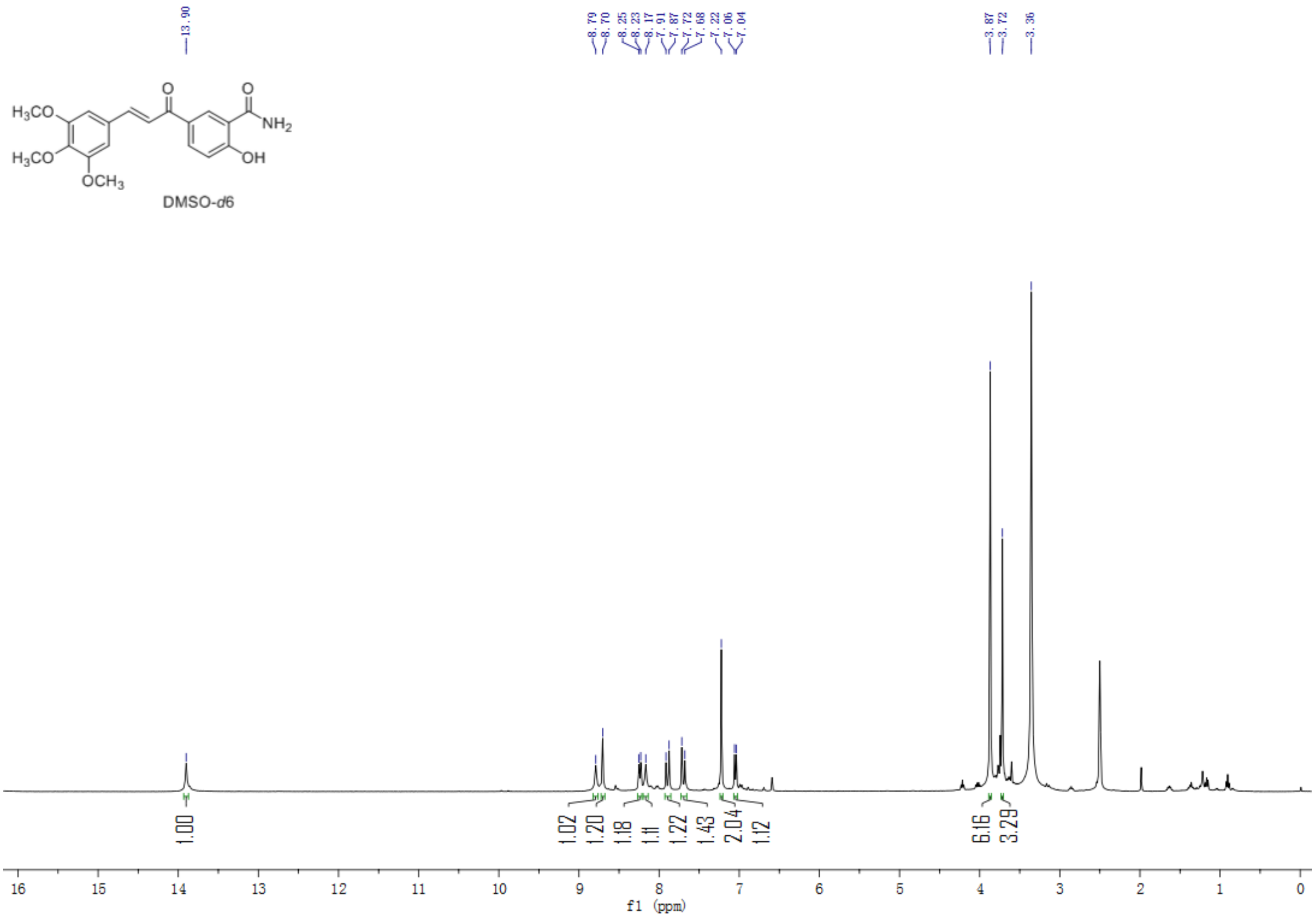

(E)-2-hydroxy-5-(3-(3,4,5-trimethoxyphenyl)acryloyl)benzamide ***(a14)***

3. C-NMR information of the synthesized compounds

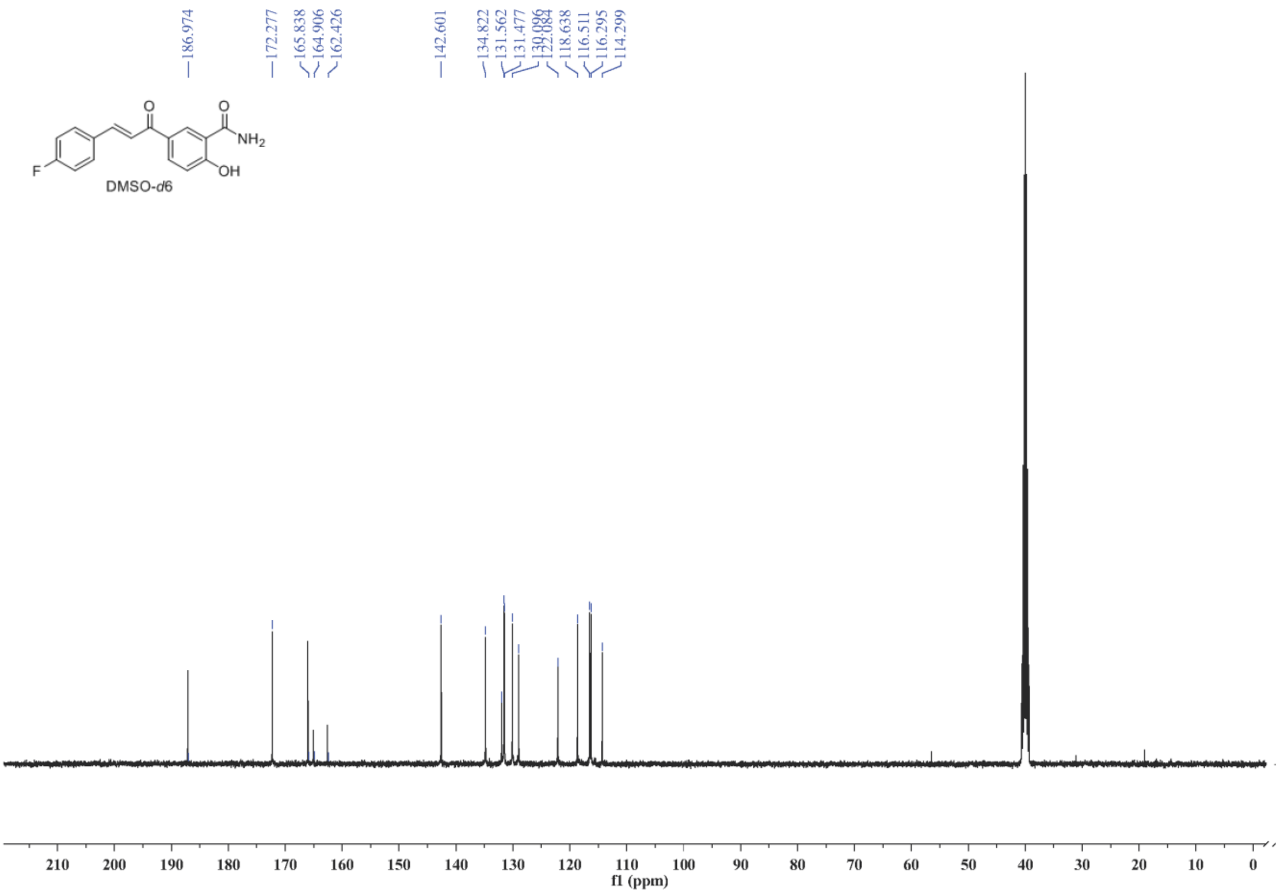

*(E)-5-(3-(4-fluorophenyl)acryloyl)-2-hydroxybenzamide (**a1**)*

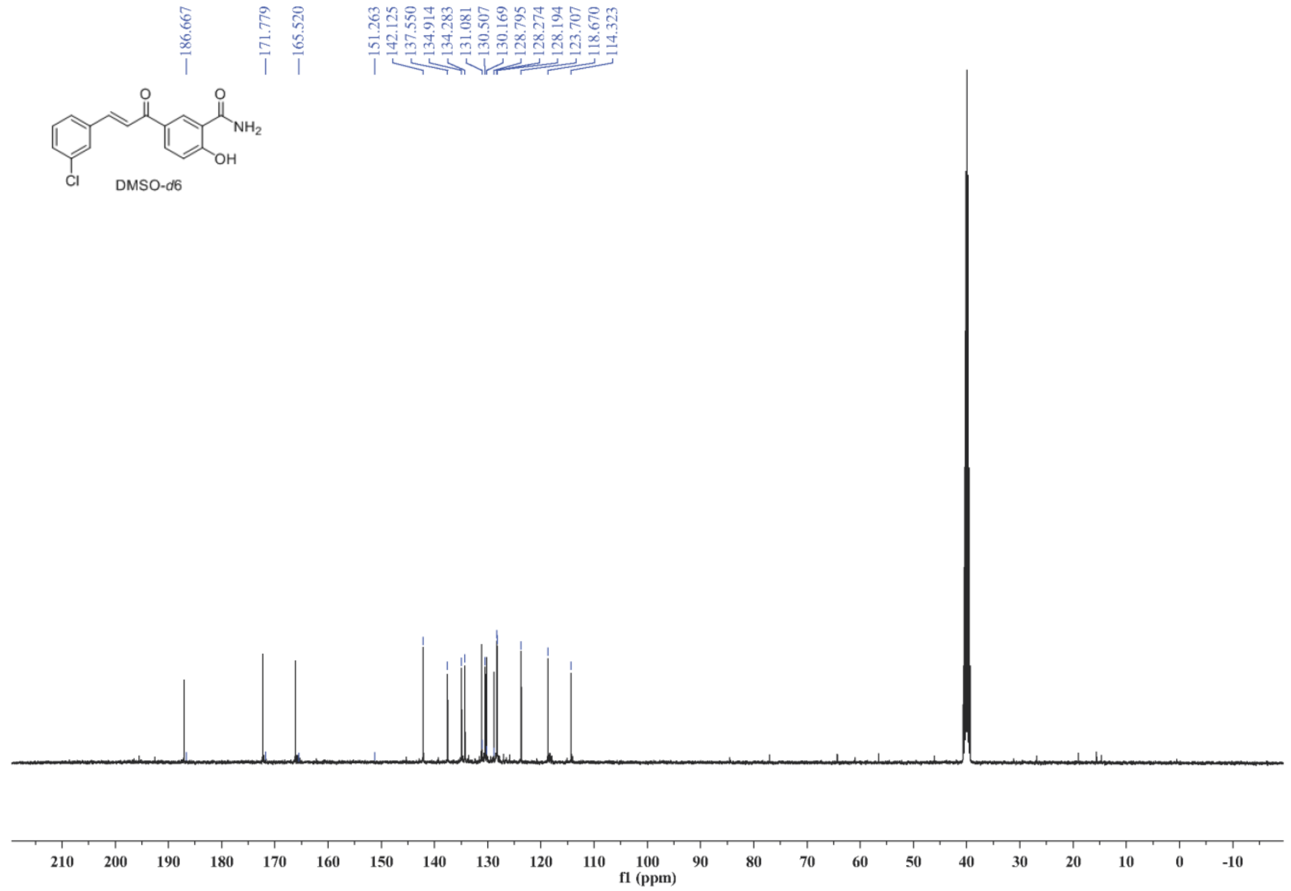

*(E)-5-(3-(3-chlorophenyl)acryloyl)-2-hydroxybenzamide (**a2**)*

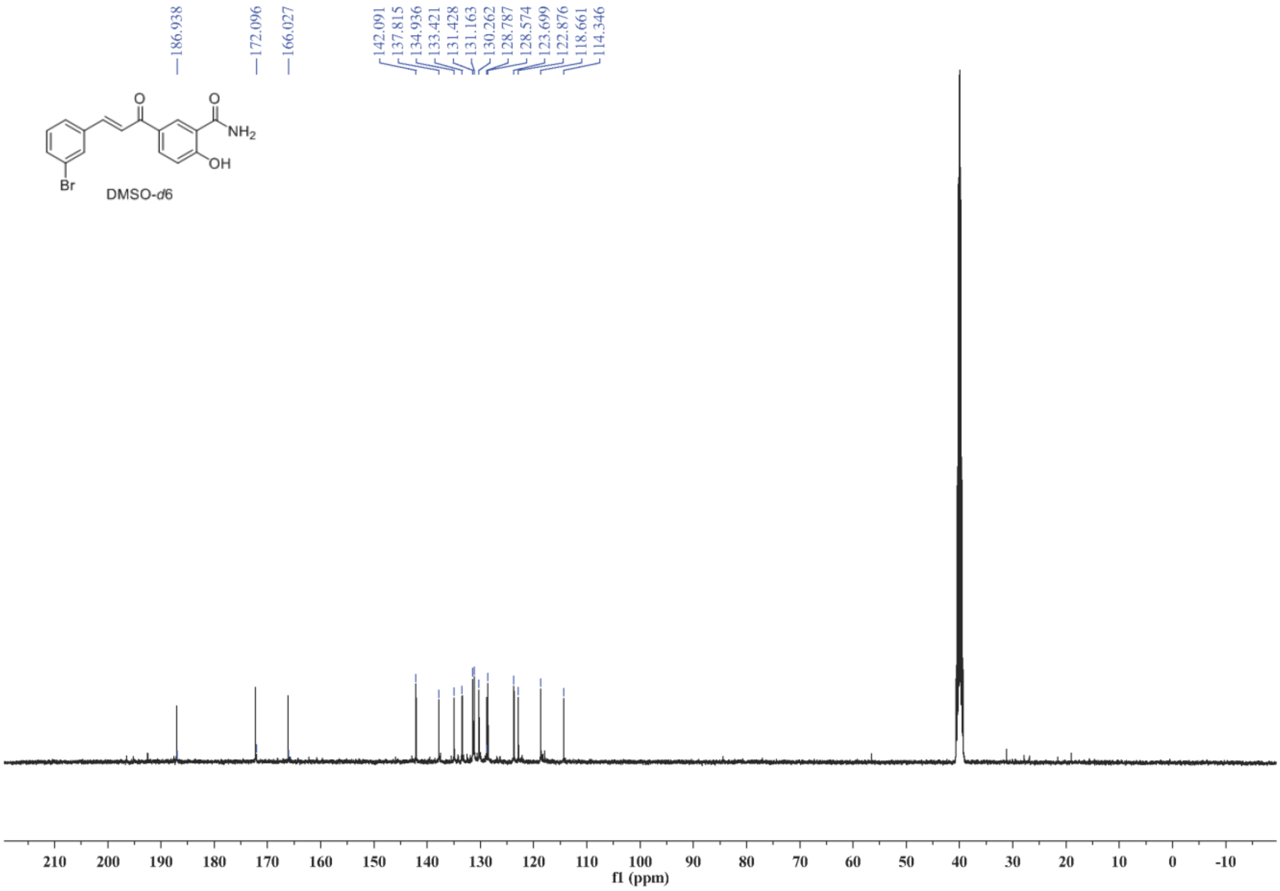

*(E)-5-(3-(3-bromophenyl)acryloyl)-2-hydroxybenzamide (**a3**)*

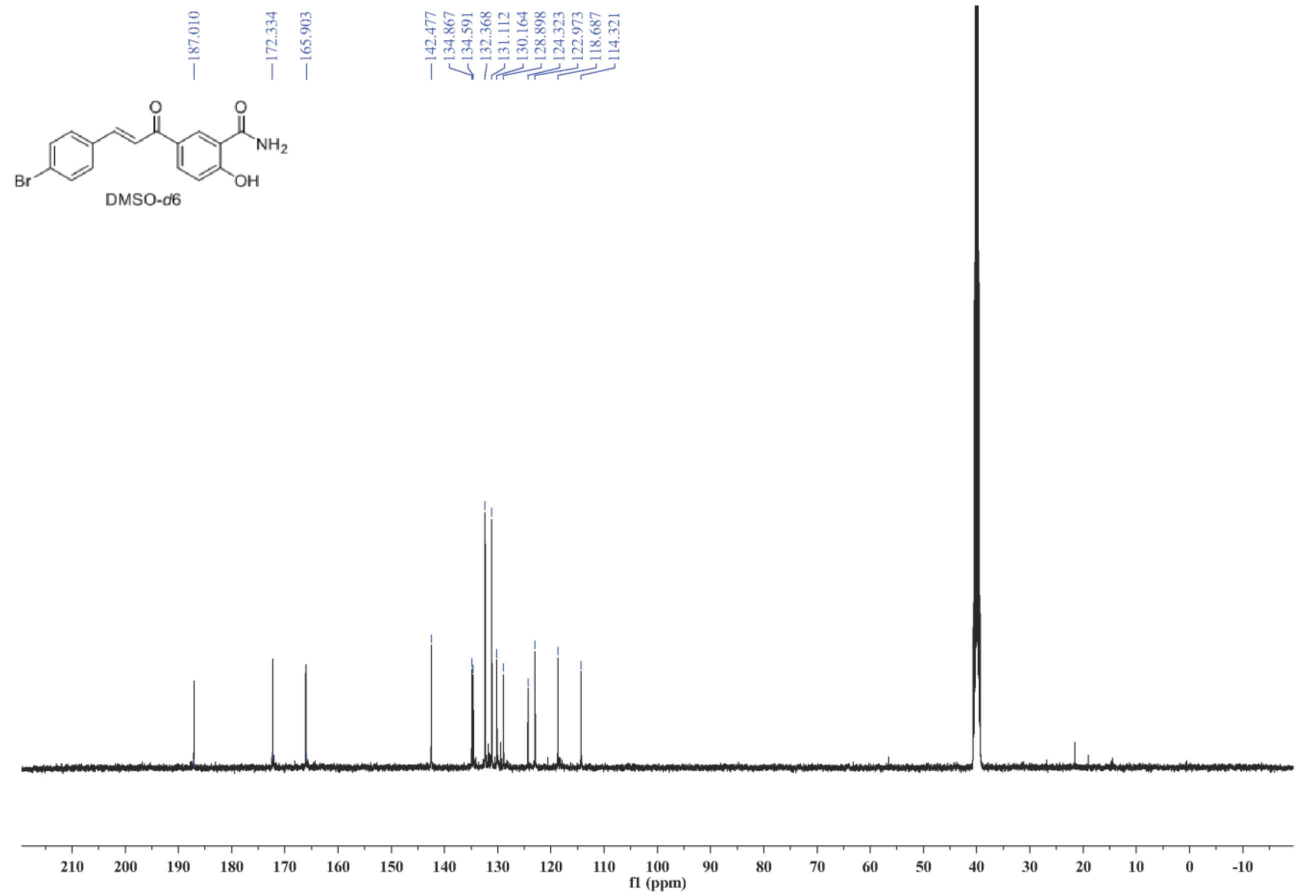

(E)-5-(3-(4-bromophenyl)acryloyl)-2-hydroxybenzamide ***(a4)***

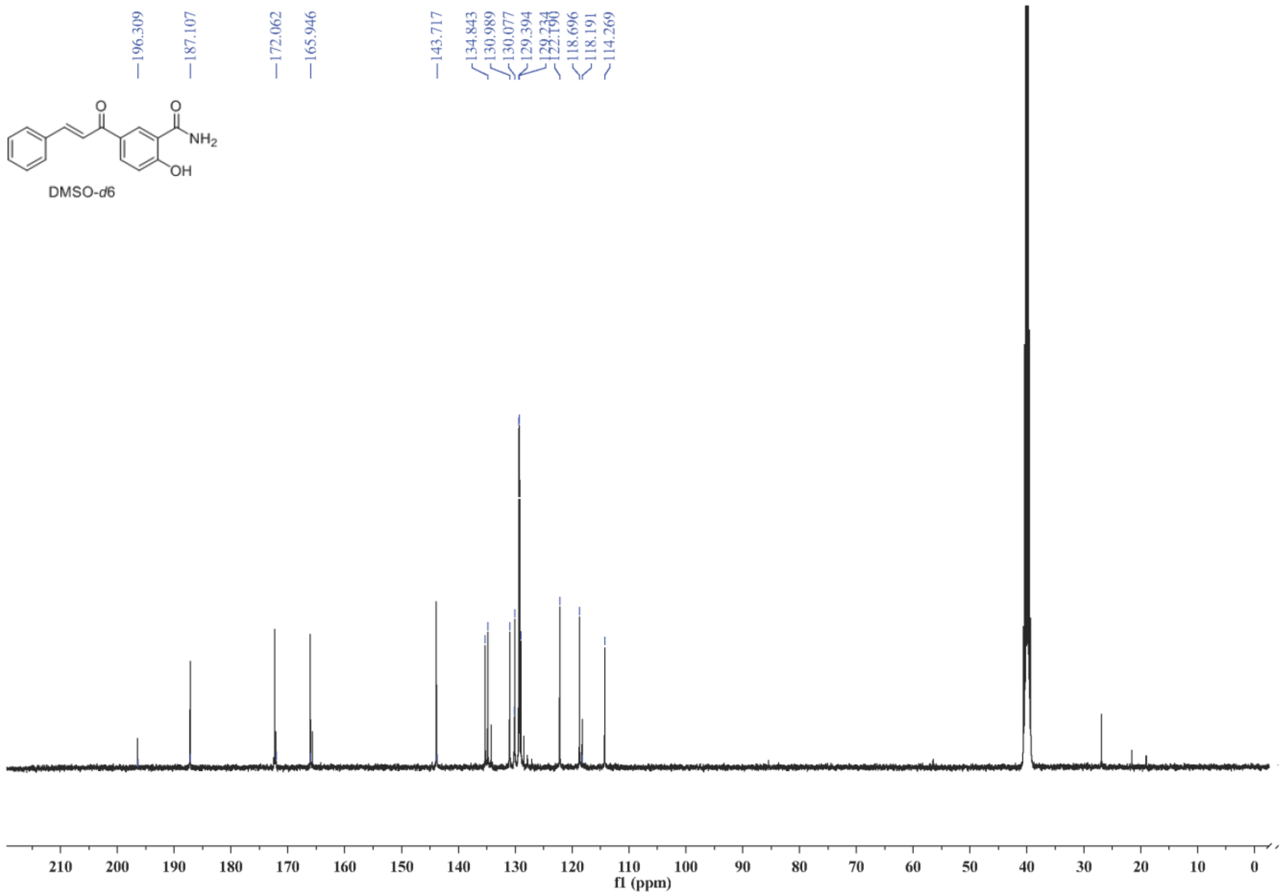

*(E)-5-cinnamoyl-2-hydroxybenzamide (**a5**)*

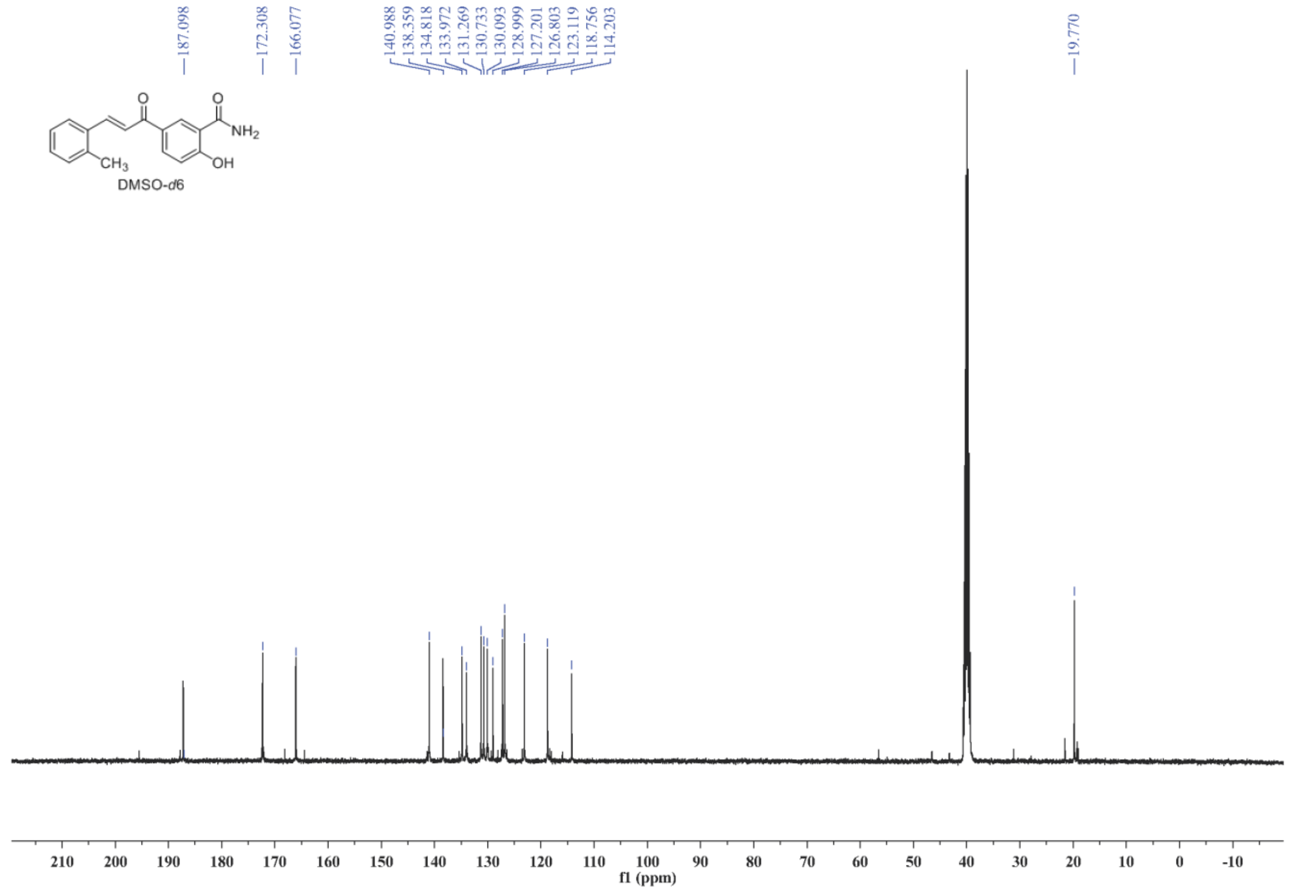

*(E)-2-hydroxy-5-(3-(o-tolyl)acryloyl)benzamide(**a6**)*

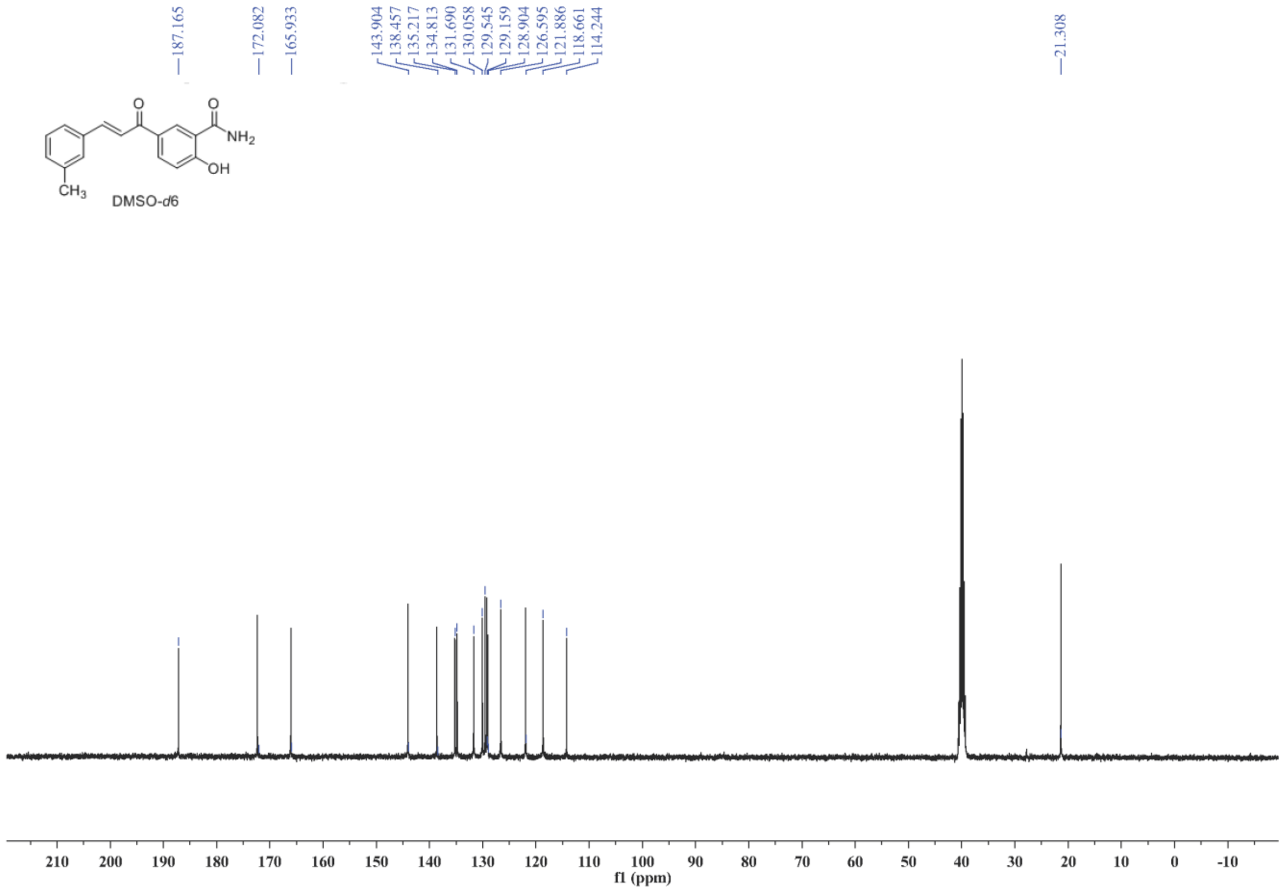

*(E)-2-hydroxy-5-(3-(m-tolyl)acryloyl)benzamide (**a**7)*

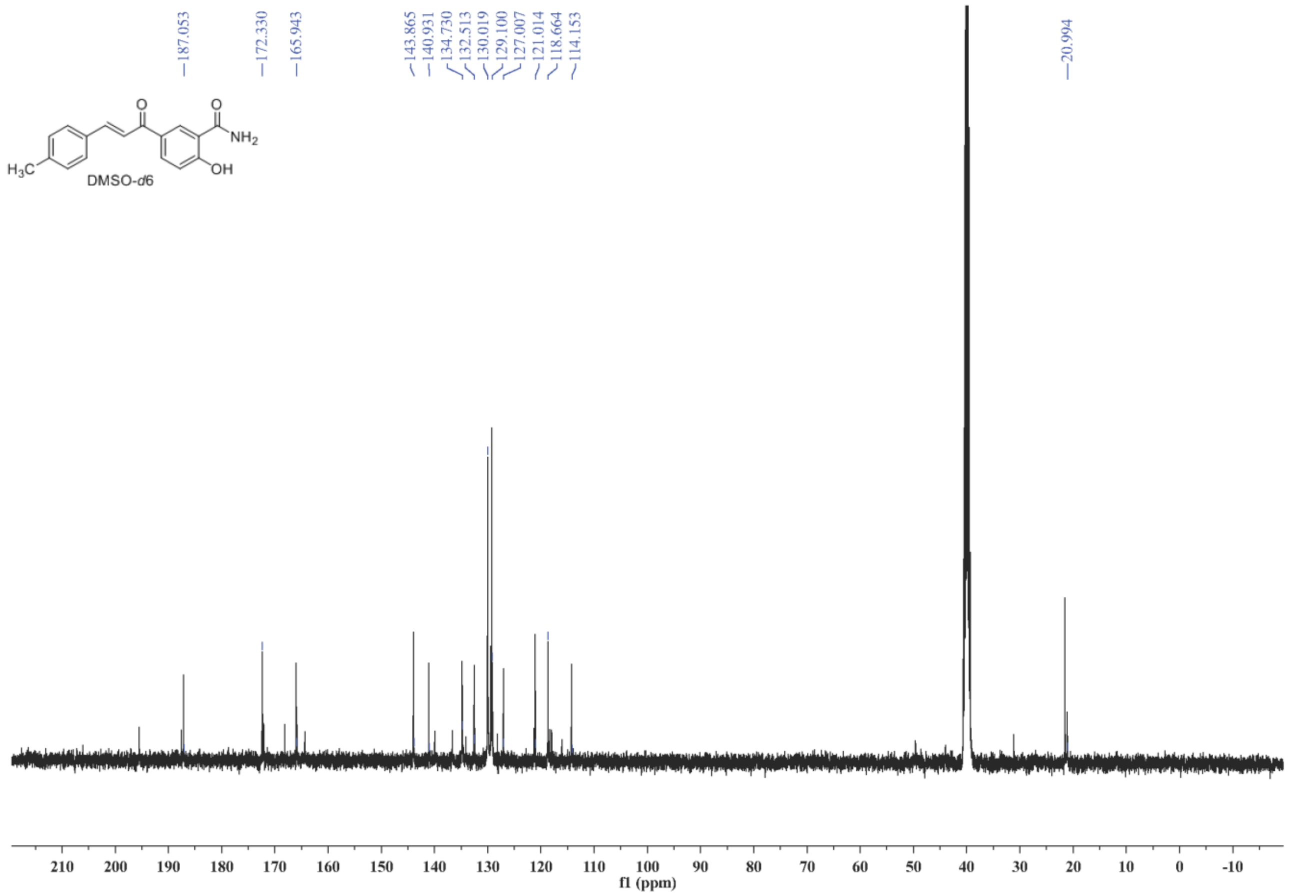

*(E)-2-hydroxy-5-(3-(p-tolyl)acryloyl)benzamide (**a8**)*

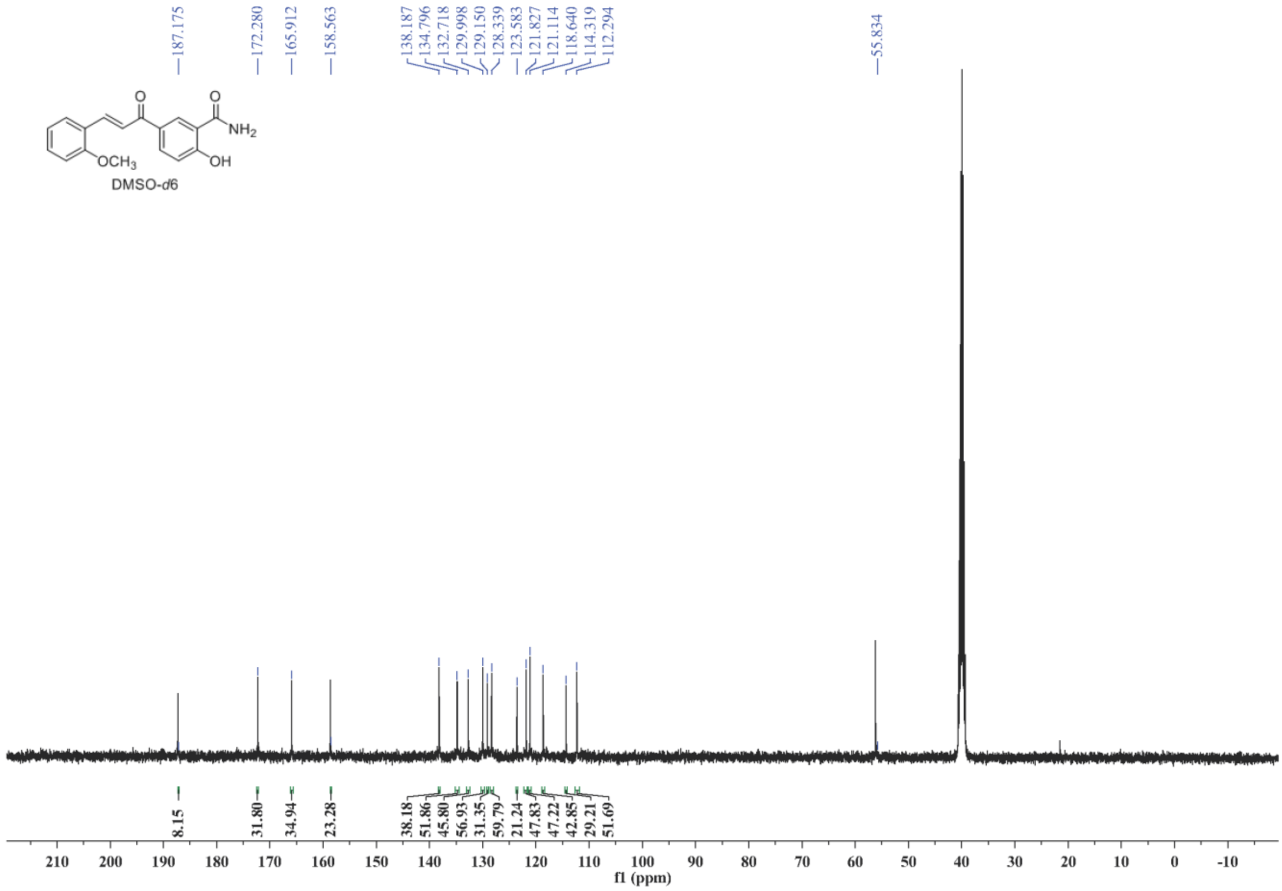

*(E)-2-hydroxy-5-(3-(2-methoxyphenyl)acryloyl)benzamide(**a9**)*

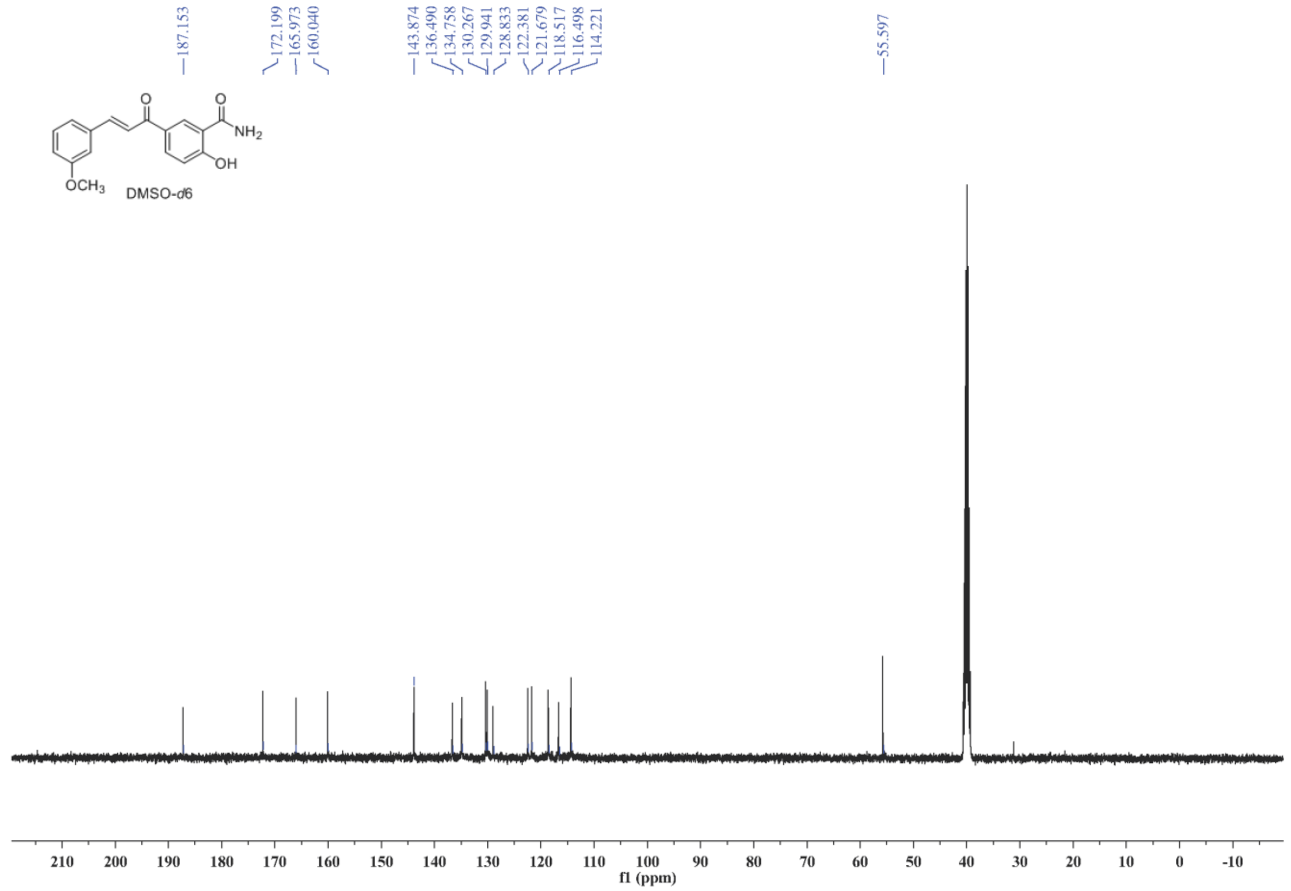

*(E)-2-hydroxy-5-(3-(3-methoxyphenyl)acryloyl)benzamide(**a10**)*

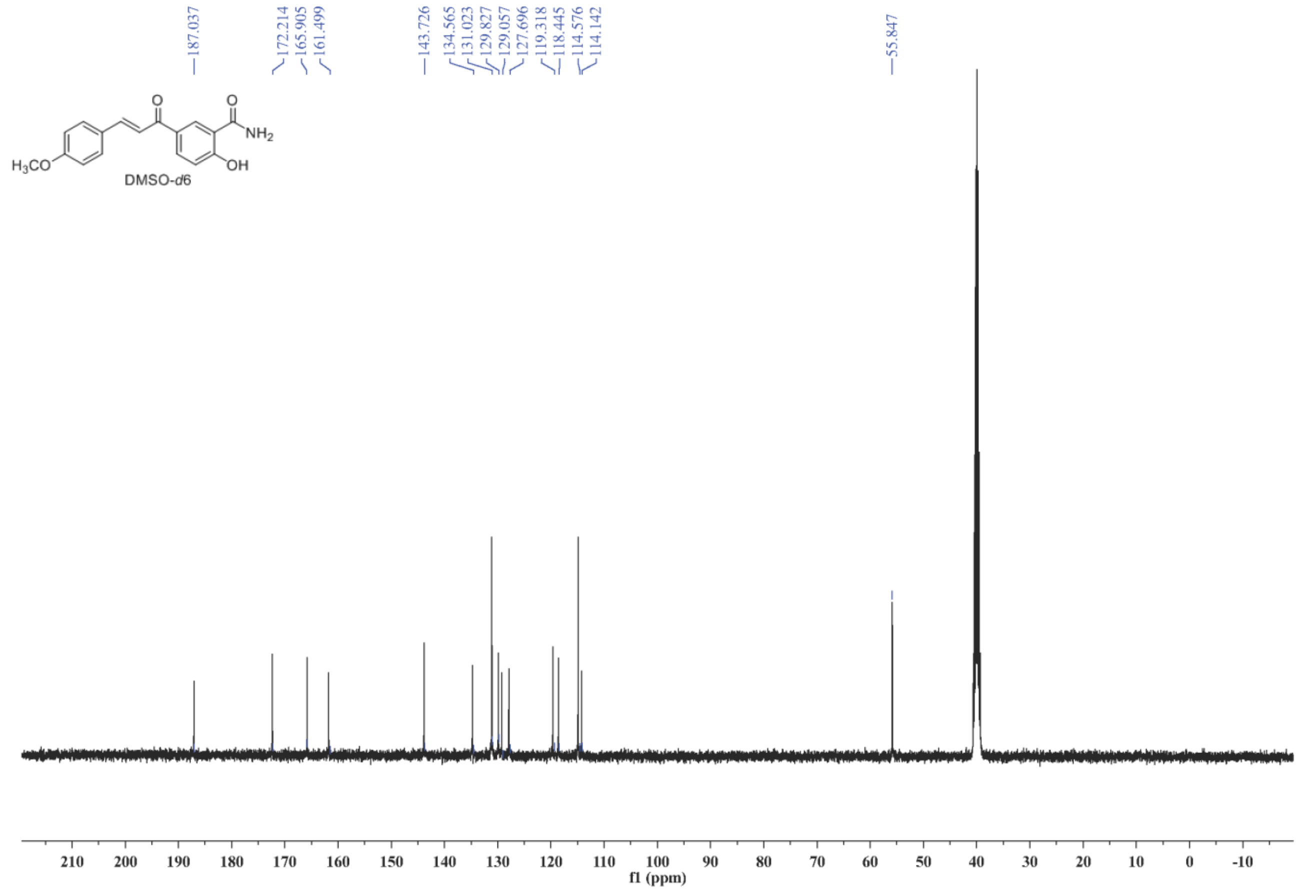

*(E)-2-hydroxy-5-(3-(4-methoxyphenyl)acryloyl)benzamide(**a11**)*

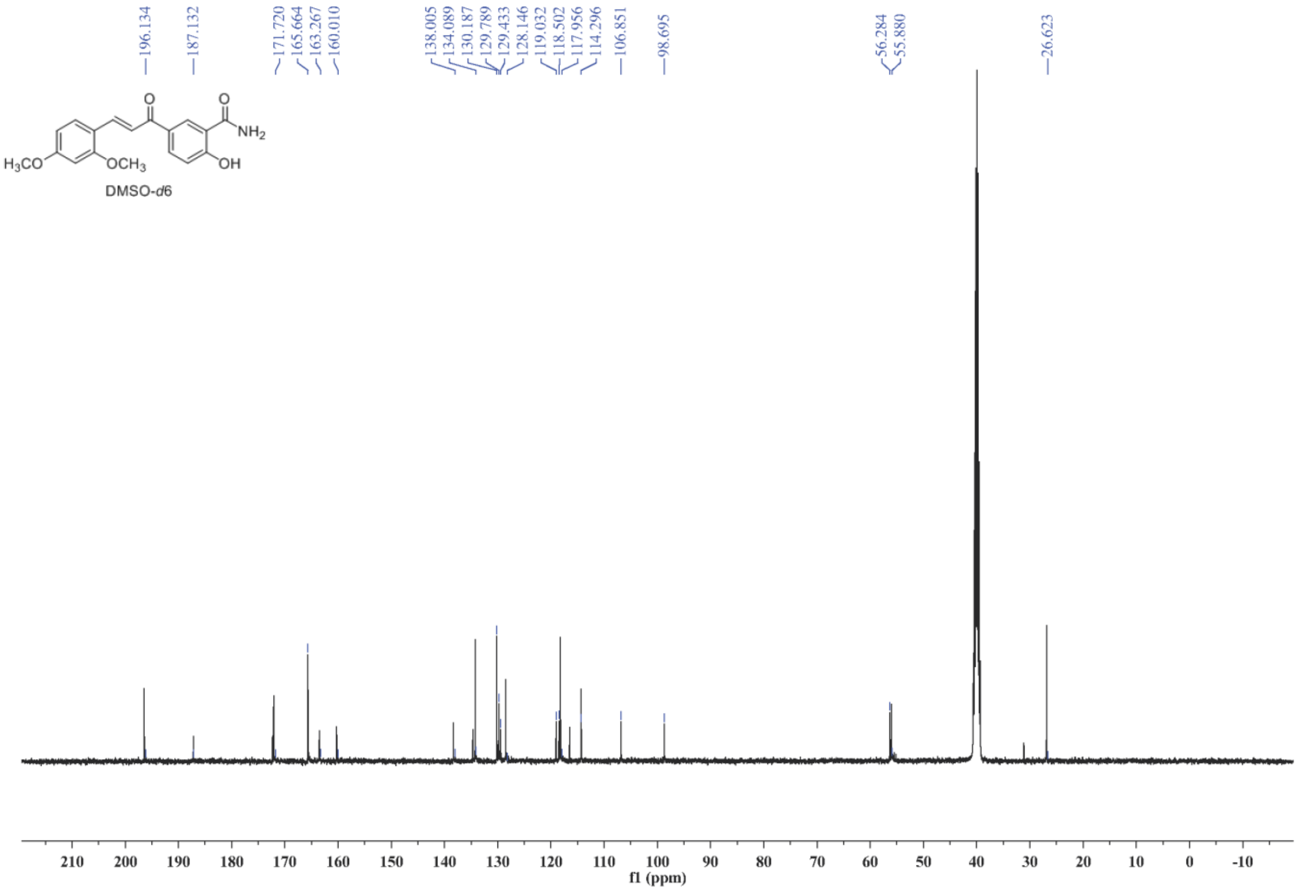

(E)-5-(3-(2,4-dimethoxyphenyl)acryloyl)-2-hydroxybenzamide ***(a12)***

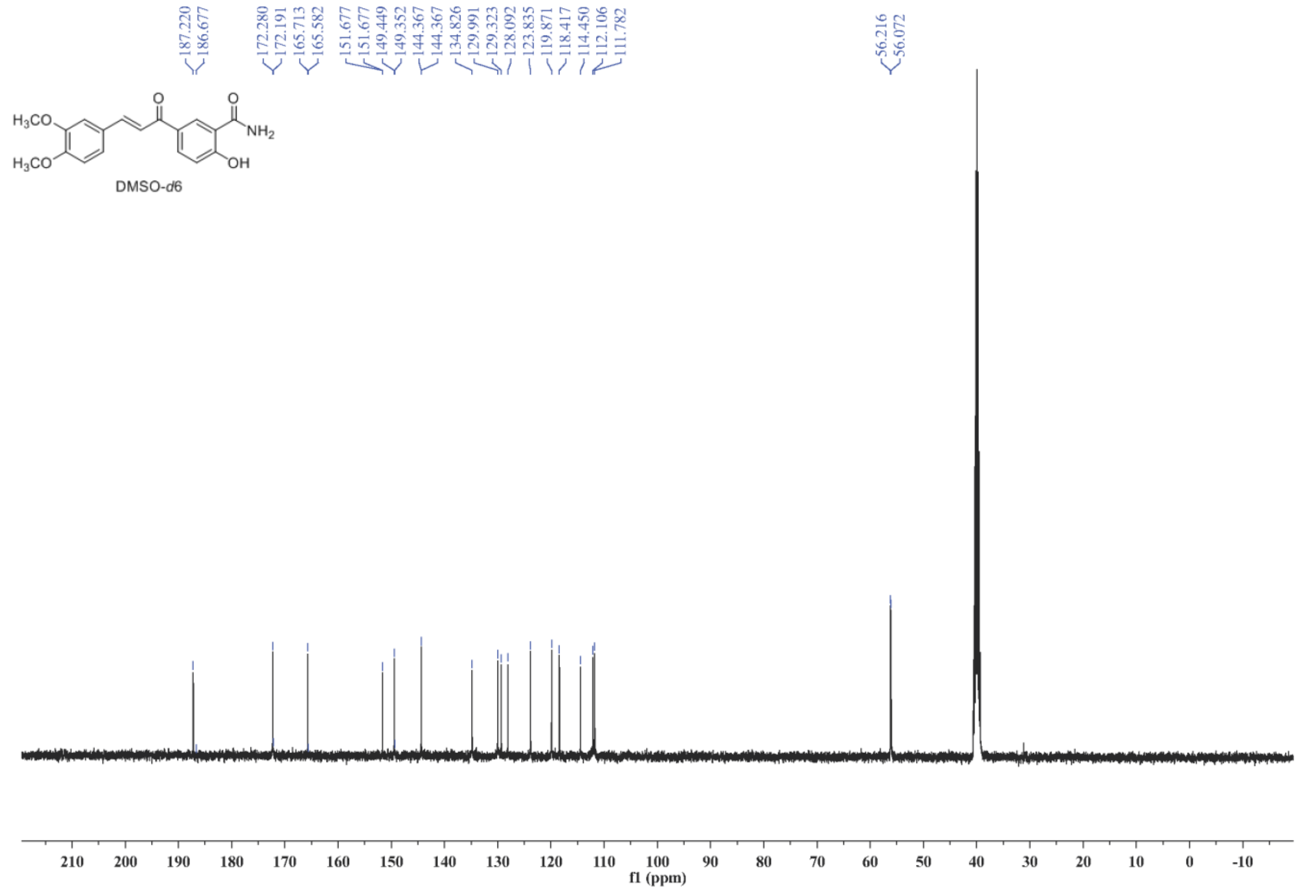

*(E)-5-(3-(3,4-dimethoxyphenyl)acryloyl)-2-hydroxybenzamide **(a13)***

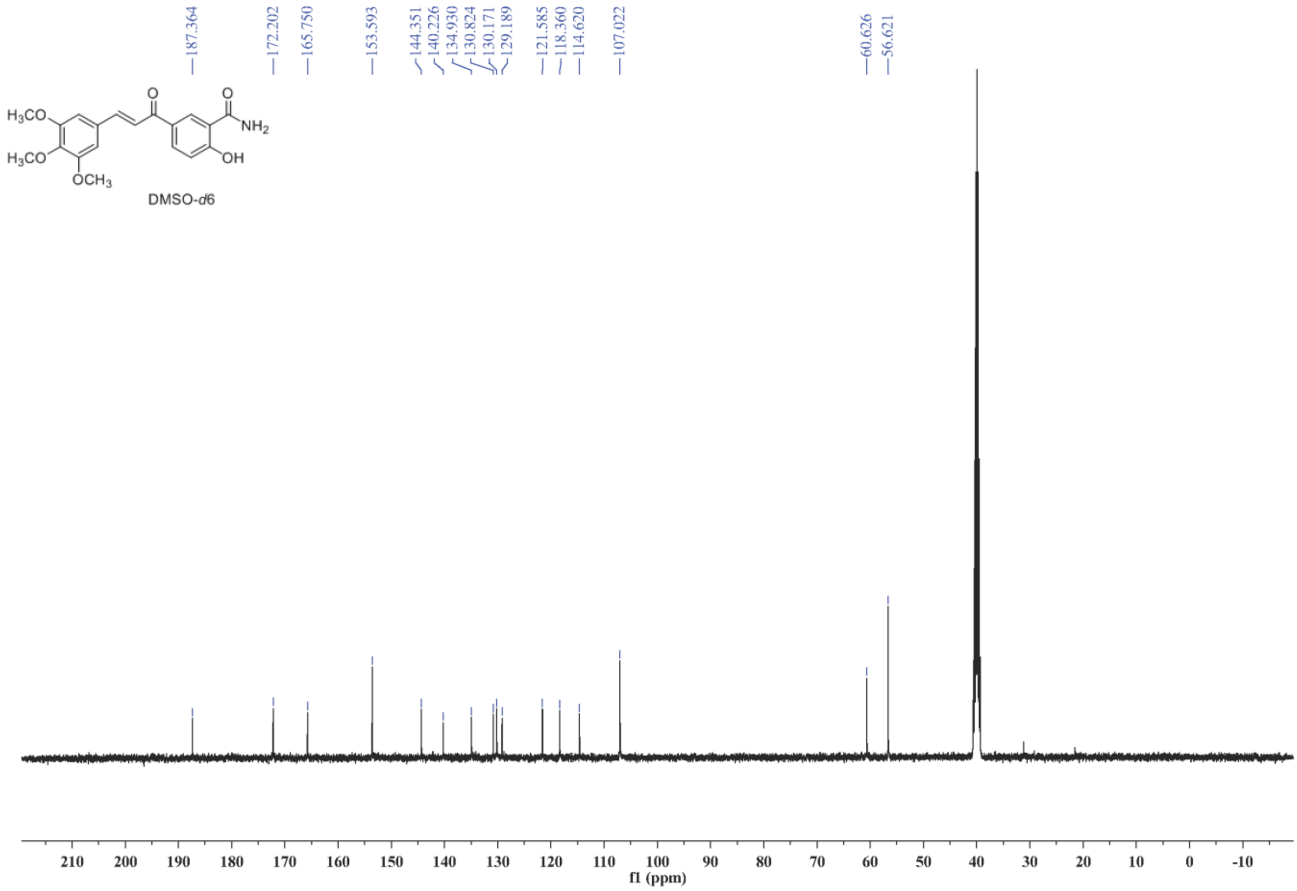

(E)-2-hydroxy-5-(3-(3,4,5-trimethoxyphenyl)acryloyl)benzamide ***(a14)***

4. HRMS information of the synthesized compounds.

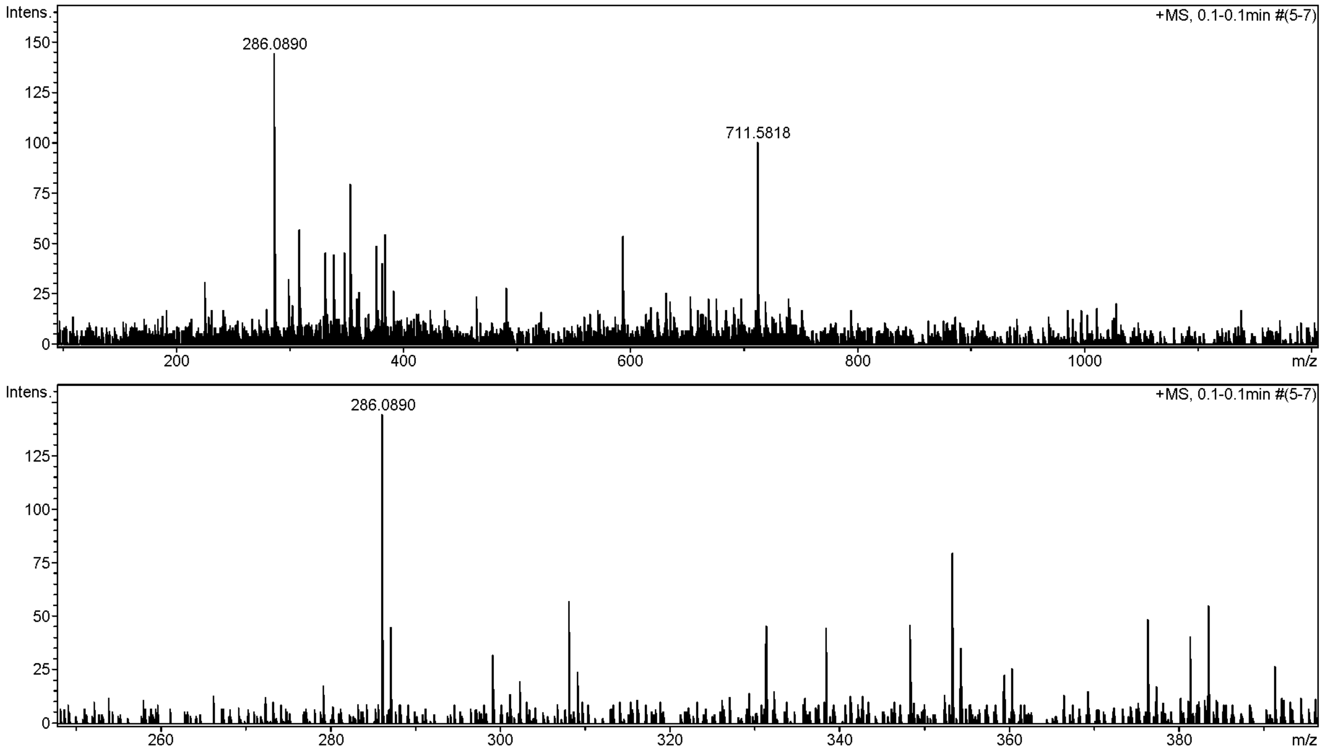

*(E)-5-(3-(4-fluorophenyl)acryloyl)-2-hydroxybenzamide (**a1**)*

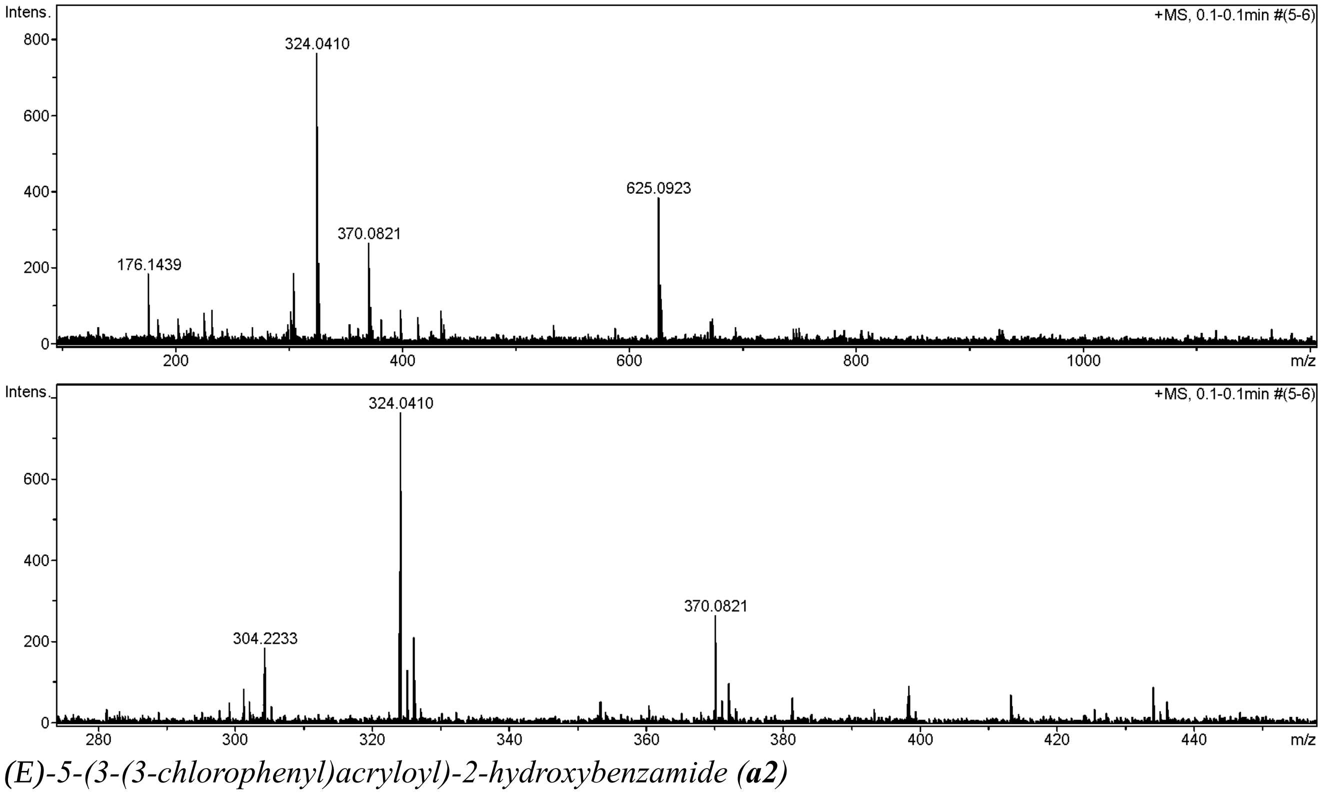

*(E)-5-(3-(3-chlorophenyl)acryloyl)-2-hydroxybenzamide (**a2**)*

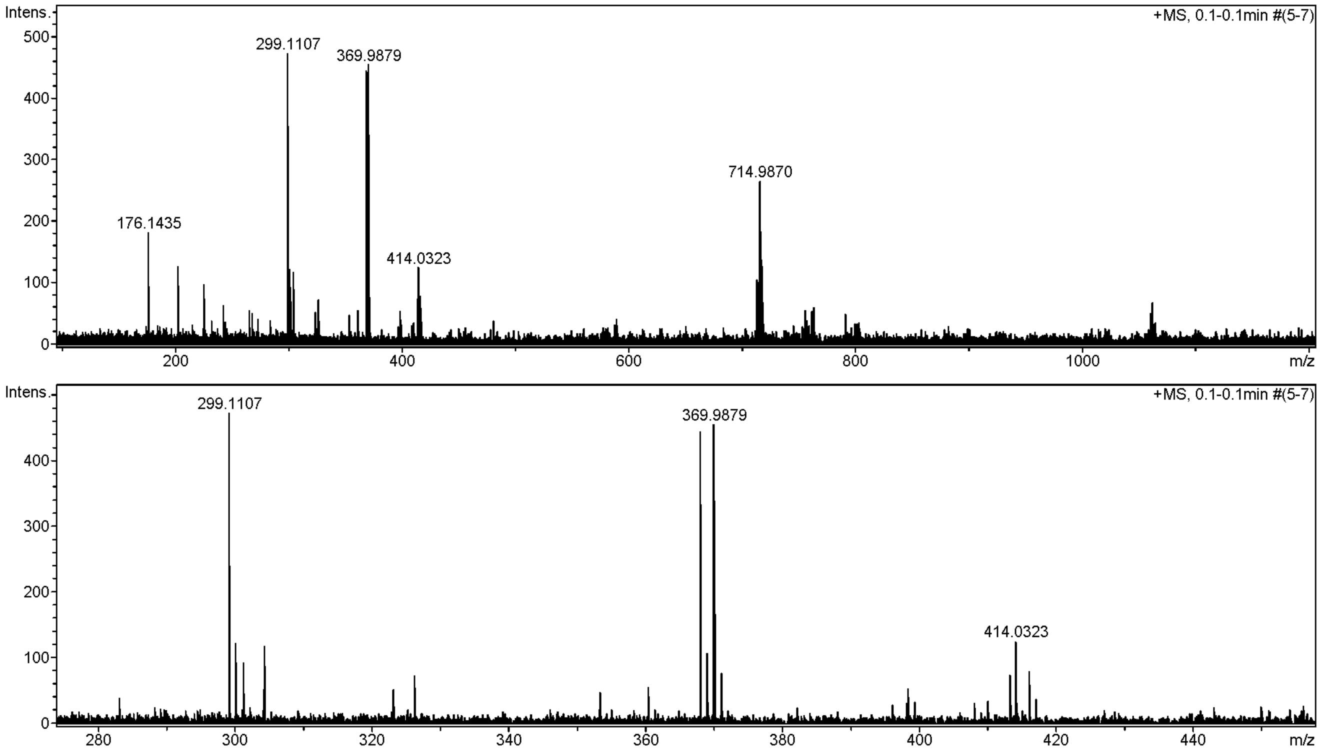

*(E)-5-(3-(3-bromophenyl)acryloyl)-2-hydroxybenzamide (**a3**)*

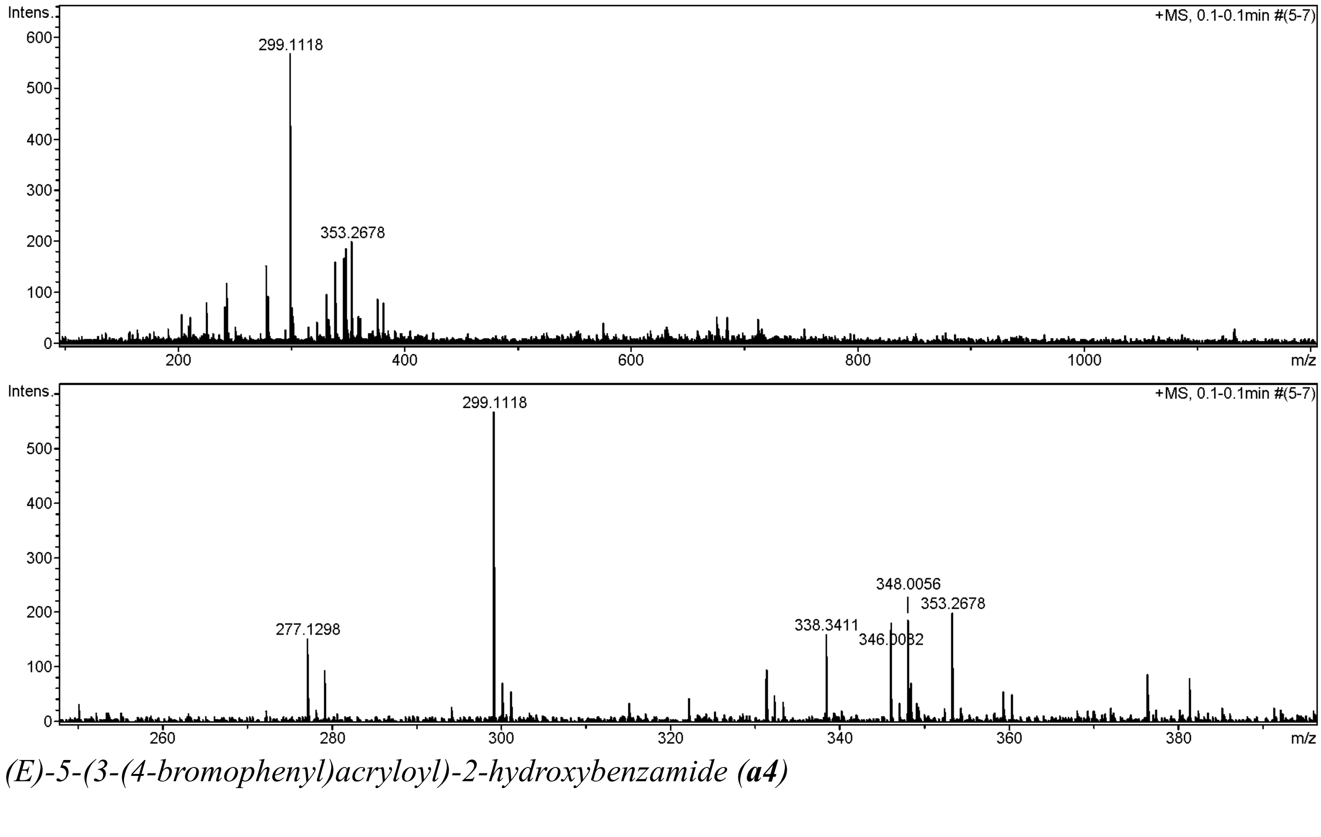

*(E)-5-(3-(4-bromophenyl)acryloyl)-2-hydroxybenzamide (**a4**)*

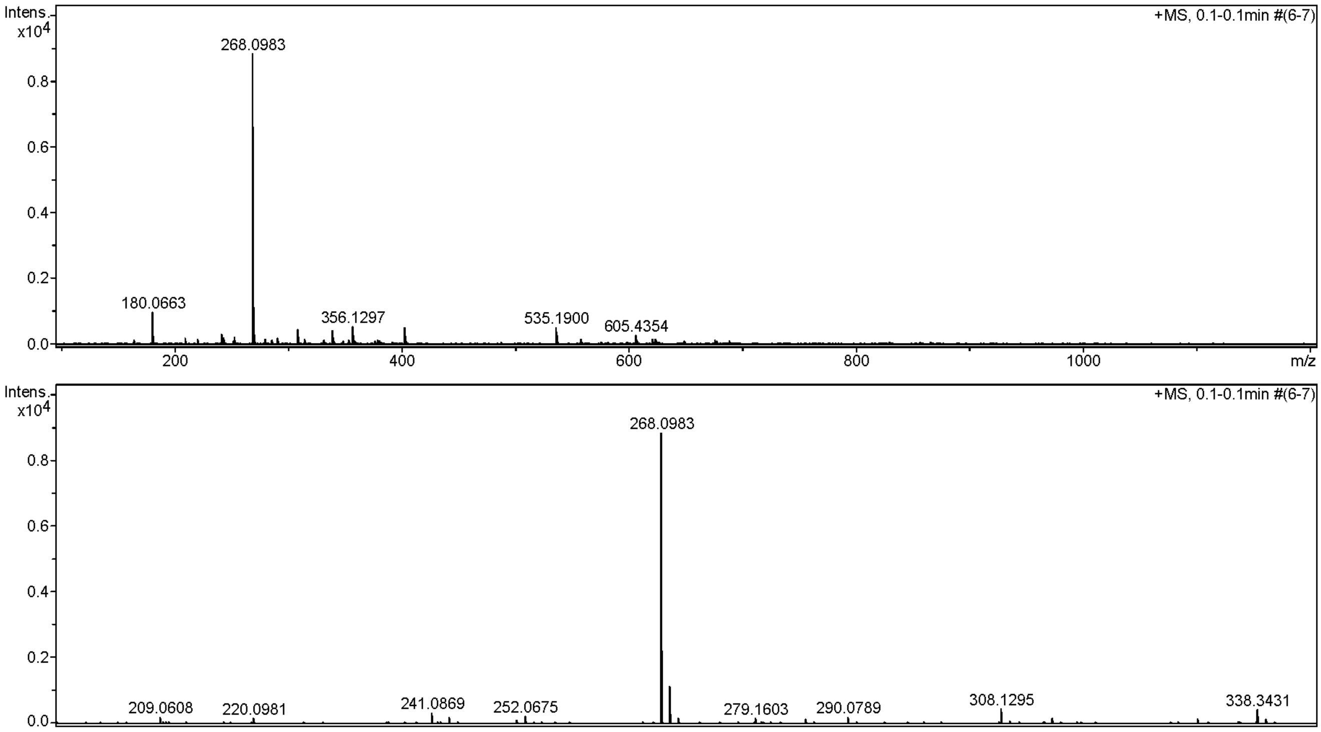

*(E)-5-cinnamoyl-2-hydroxybenzamide (**a5**)*

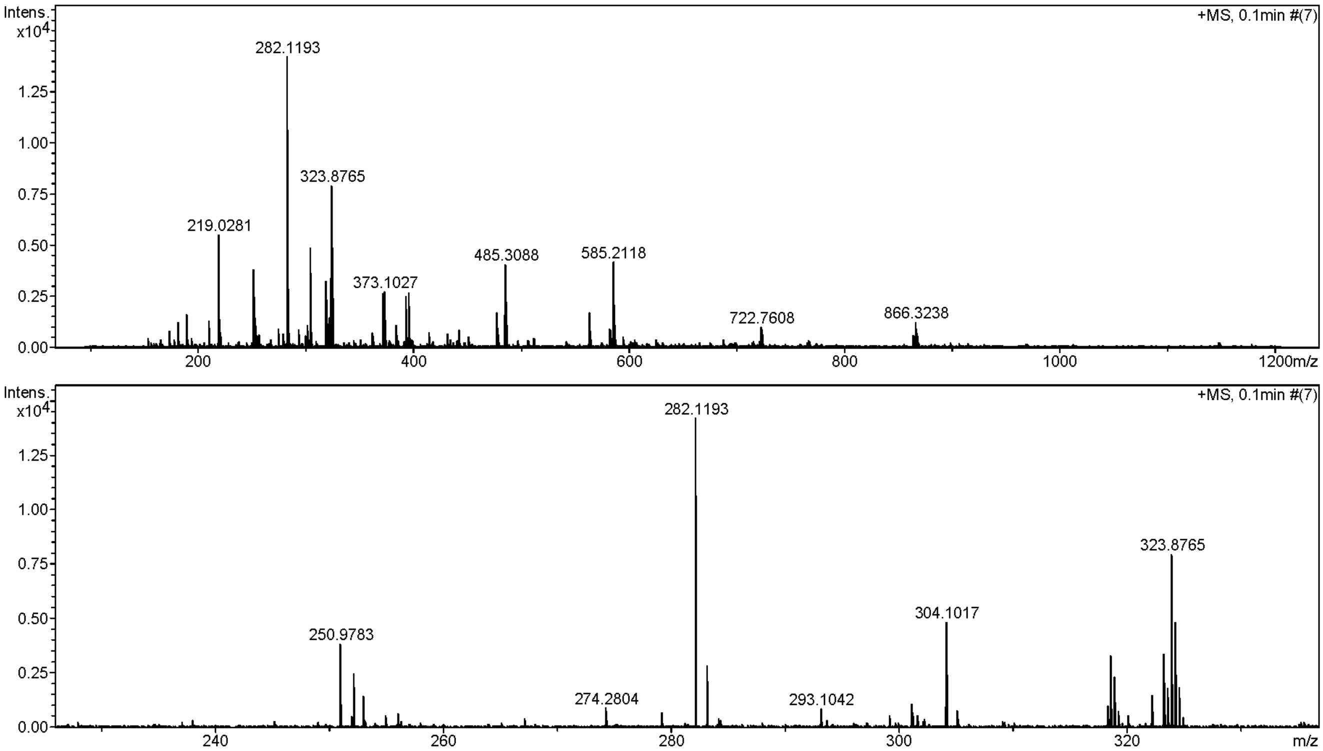

*(E)-2-hydroxy-5-(3-(o-tolyl)acryloyl)benzamide (**a6**)*

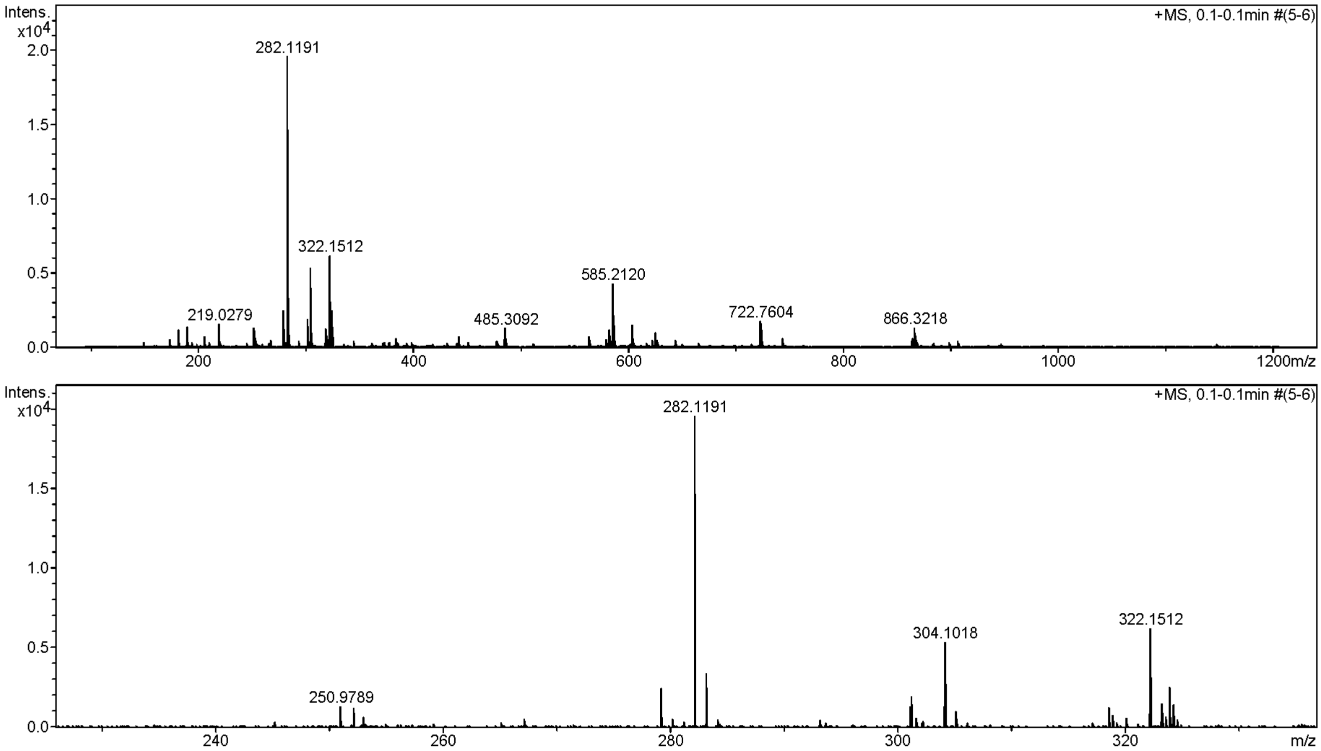

*(E)-2-hydroxy-5-(3-(m-tolyl)acryloyl)benzamide (**a7**)*

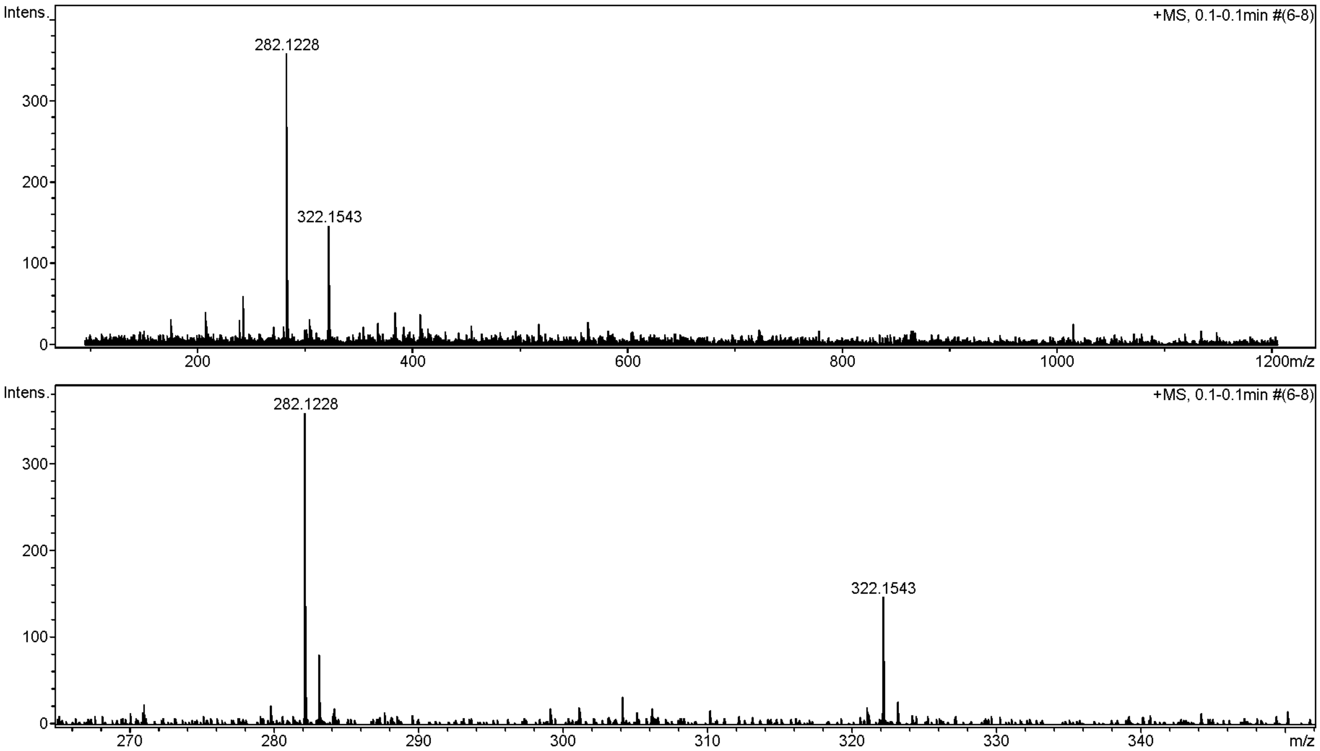

*(E)-2-hydroxy-5-(3-(p-tolyl)acryloyl)benzamide (**a8**)*

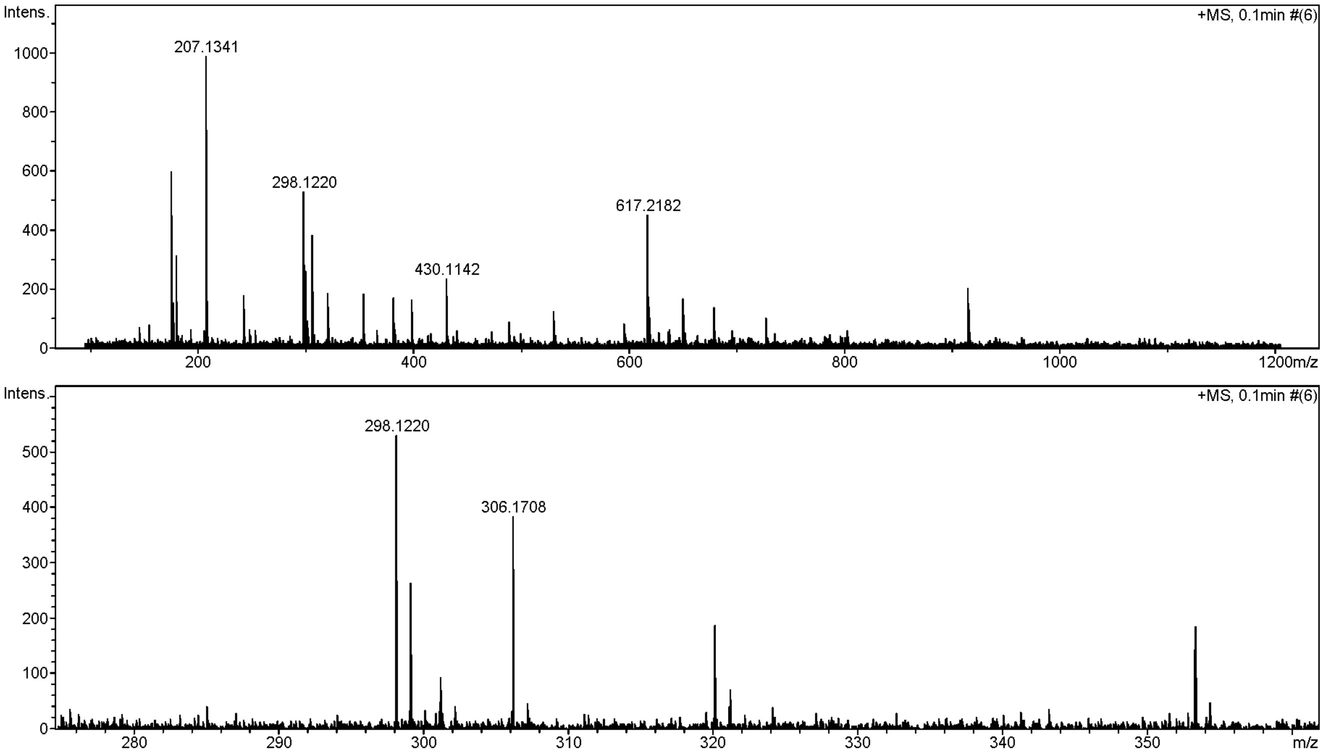

*(E)-2-hydroxy-5-(3-(2-methoxyphenyl)acryloyl)benzamide (**a9**)*

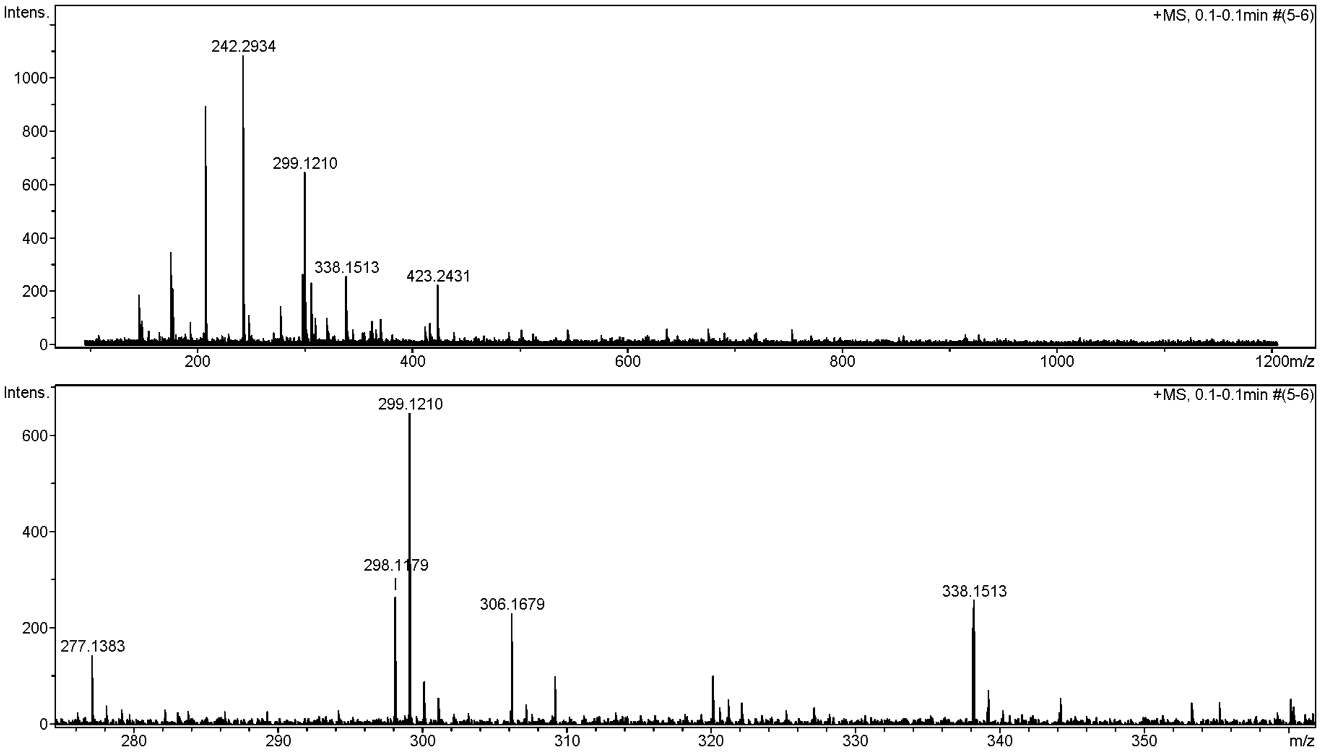

*(E)-2-hydroxy-5-(3-(3-methoxyphenyl)acryloyl)benzamide(**a10**)*

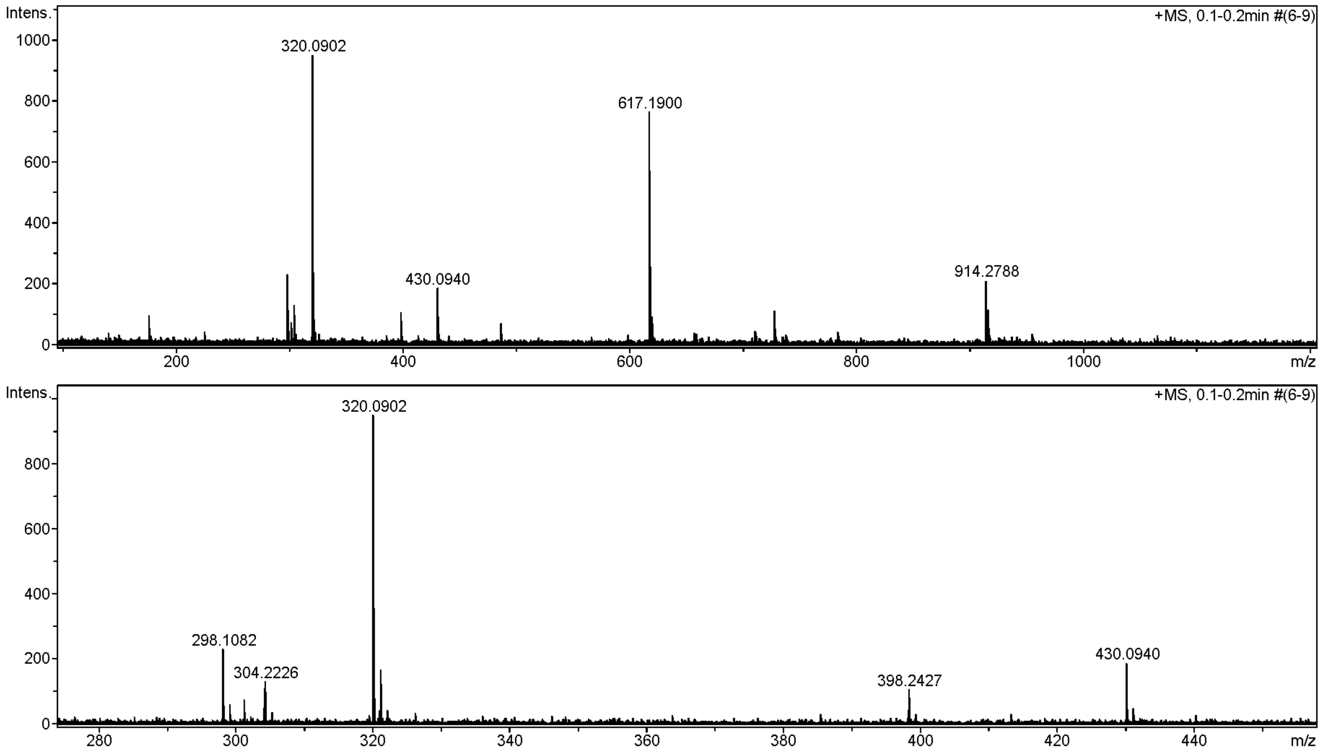

(*E*)*-2-hydroxy-5-(3-(4-methoxyphenyl)acryloyl)benzamide(**a11**)*

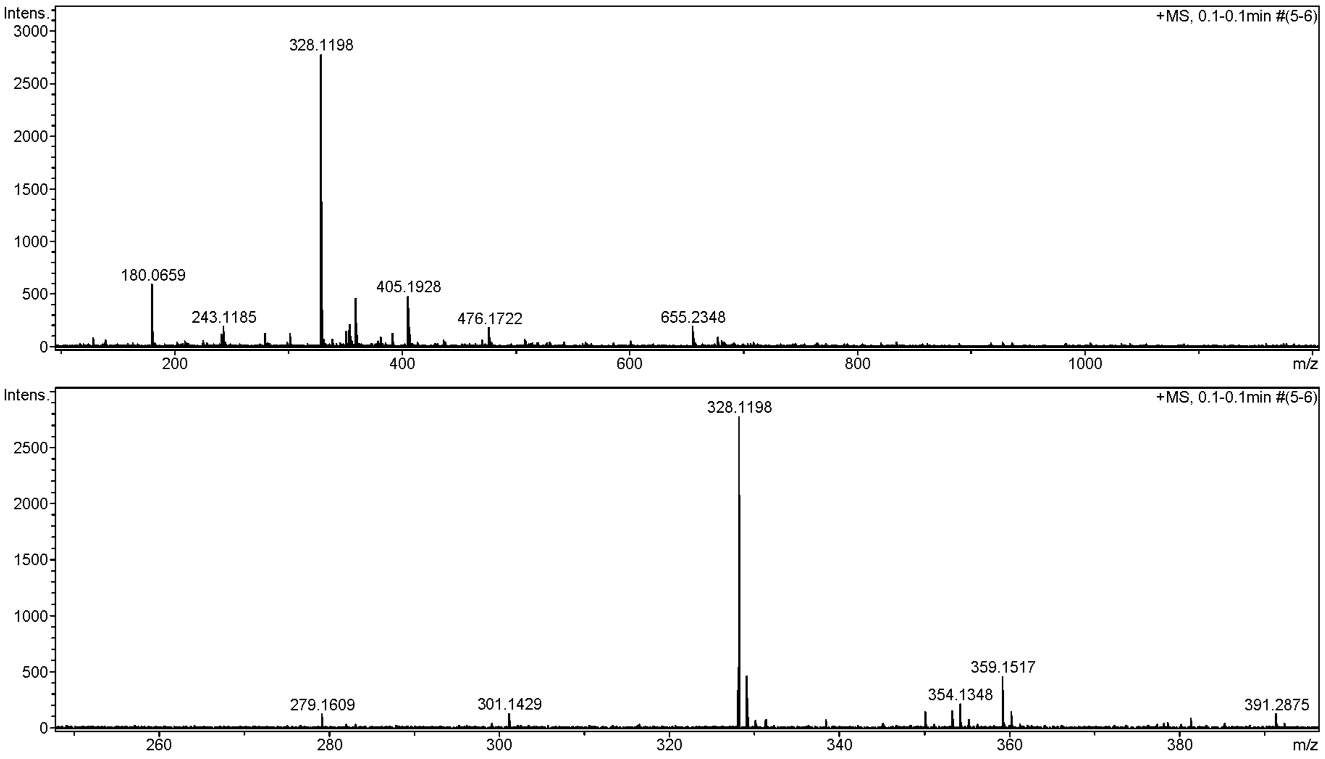

*(E)-5-(3-(2,4-dimethoxyphenyl)acryloyl)-2-hydroxybenzamide (**a12**)*

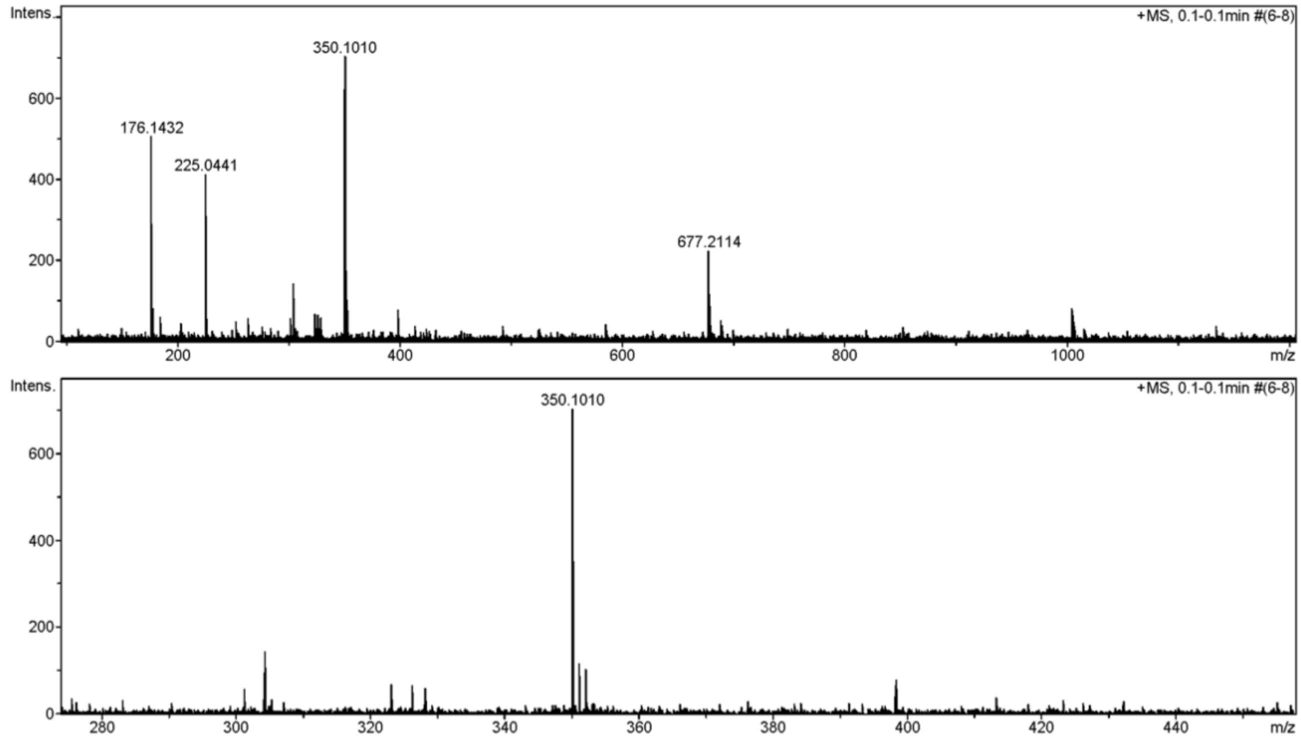

*(E)-5-(3-(3,4-dimethoxyphenyl)acryloyl)-2-hydroxybenzamide (**a13**)*

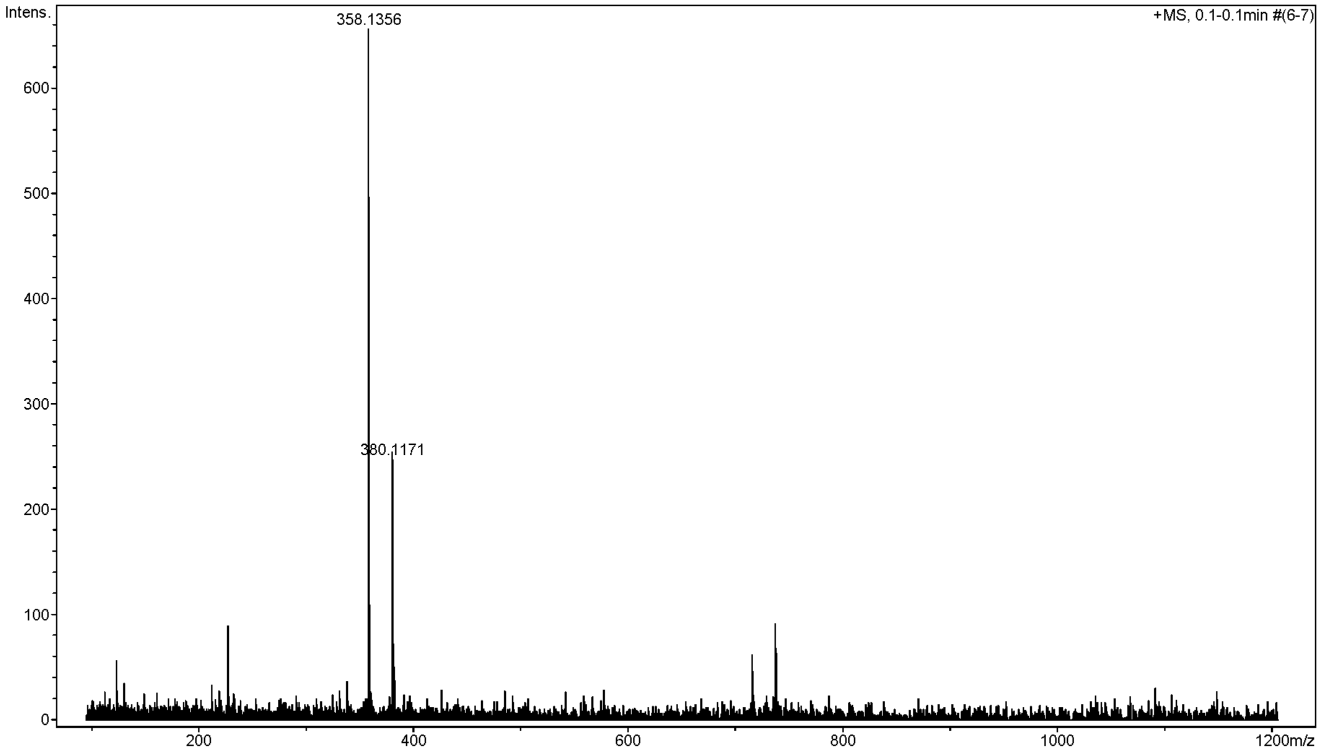

*(F)-2-hydroxy-5-(3-(3,4,5-trimethoxyphenyl)acryloyl)benzamide (**a14**)*

5. The purity information information of the synthesized compounds.

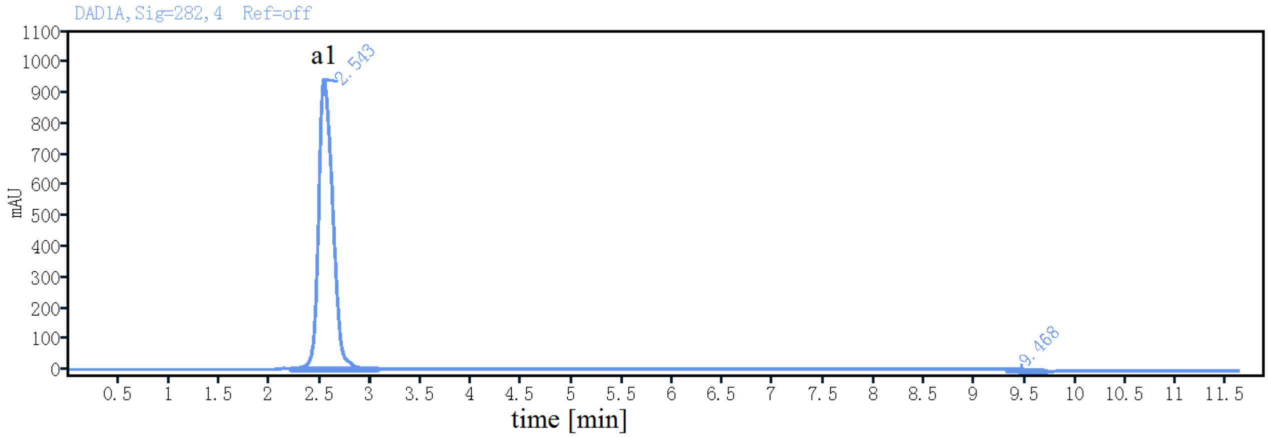

Retention time[min]	Area	Height	Area(%)
2.543	9283.22	943.09	98.97
9.468	96.94	5.76	1.03
	9380.17		

*(F)-5-(3-(4-fluorophenyl)acryloyl)-2-hydroxybenzamide (**a1**)*

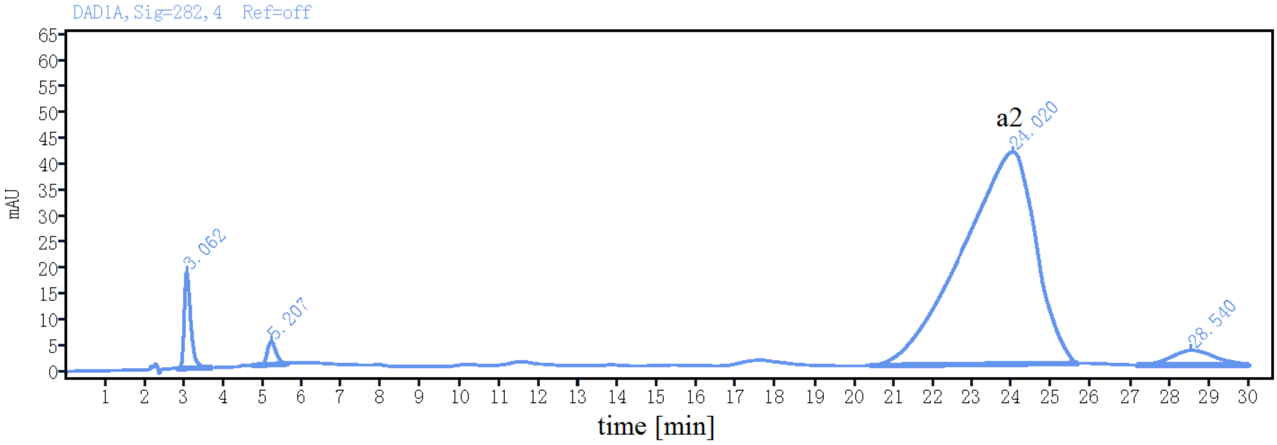

Retention time[min]	Area	Height	Area(%)
3.062	212.99	18.59	3.66
5.207	69.97	4.42	1.20
24.020	5317.79	40.97	91.39
28.540	218.31	2.84	3.75
	5819.05		

*(E)-5-(3-(3-chlorophenyl)acryloyl)-2-hydroxybenzamide (**a2**)*

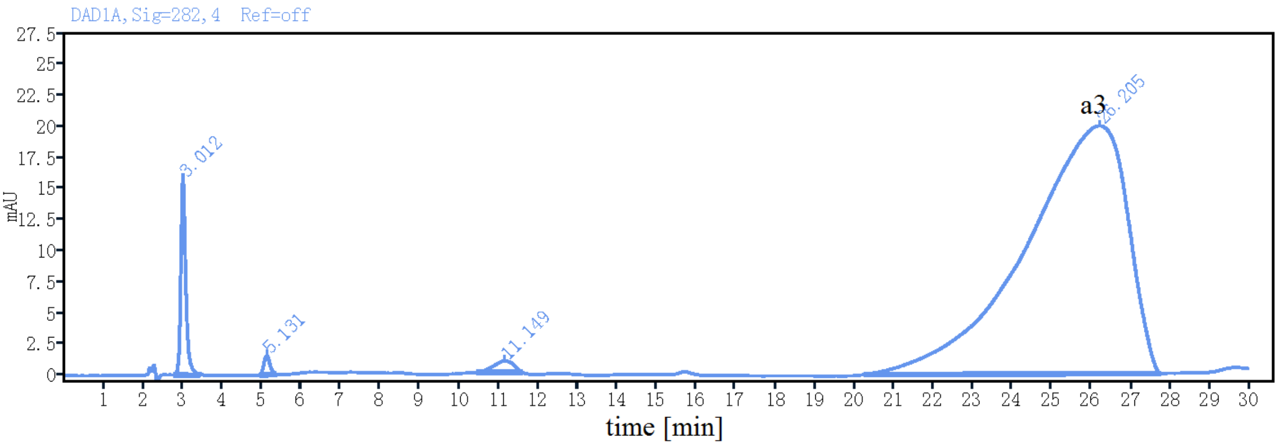

Retention time[min]	Area	Height	Area(%)
3.012	136.93	15.73	3.65
5.131	16.45	1.54	0.44
11.149	32.42	0.93	0.86
26.205	3568.92	19.94	95.05
	3754.72		

*(F)-5-(3-(3-bromophenyl)acryloyl)-2-hydroxybenzamide (**a3**)*

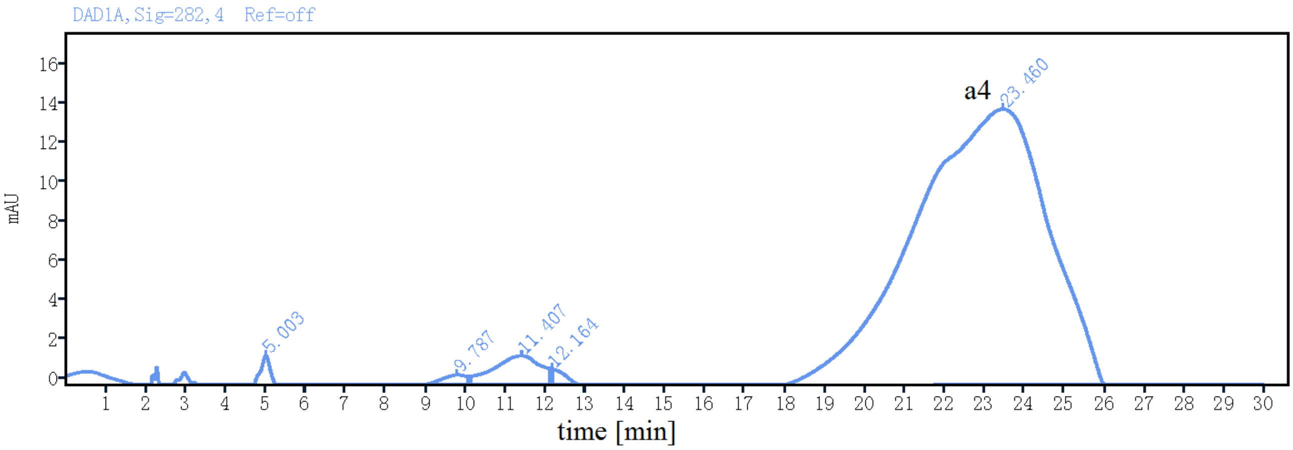

Retention time[min]	Area	Height	Area(%)
5.003	30.77	1.68	0.88
9.787	24.61	0.56	0.70
11.407	126.81	1.51	3.63
12.164	20.02	0.83	0.57
23.460	3295.37	14.09	94.22
	3497.58		

*(E)-5-(3-(4-bromophenyl)acryloyl)-2-hydroxybenzamide (**a4**)*

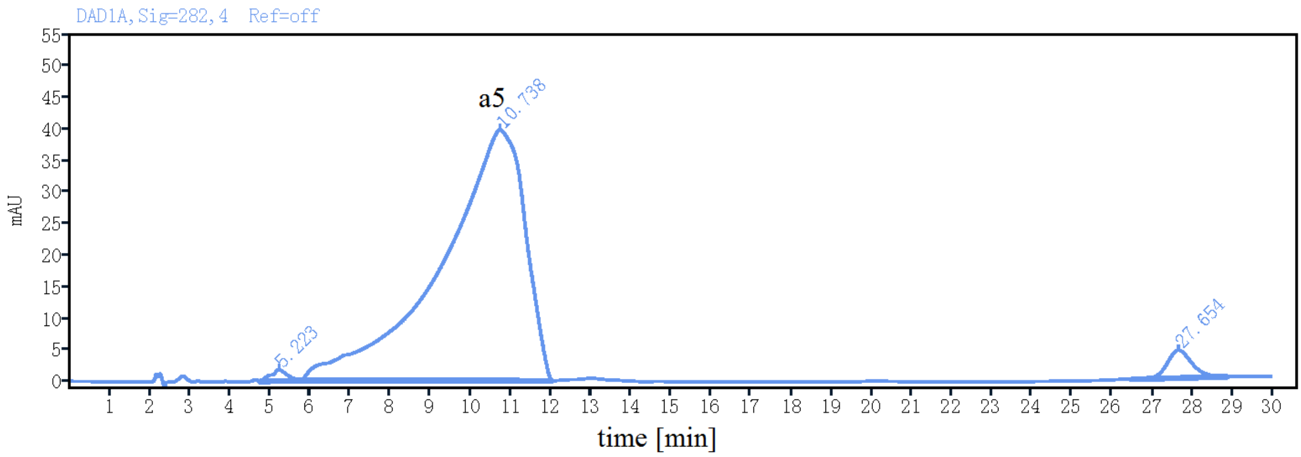

Retention time[min]	Area	Height	Area(%)
5. 223	50. 87	1. 79	0. 85
10. 738	5744. 32	39. 75	95. 93
27. 654	192. 87	4. 42	3. 22
	5988. 05		

*(F)-5-cinnamoyl-2-hydroxybenzamide (**a5**)*

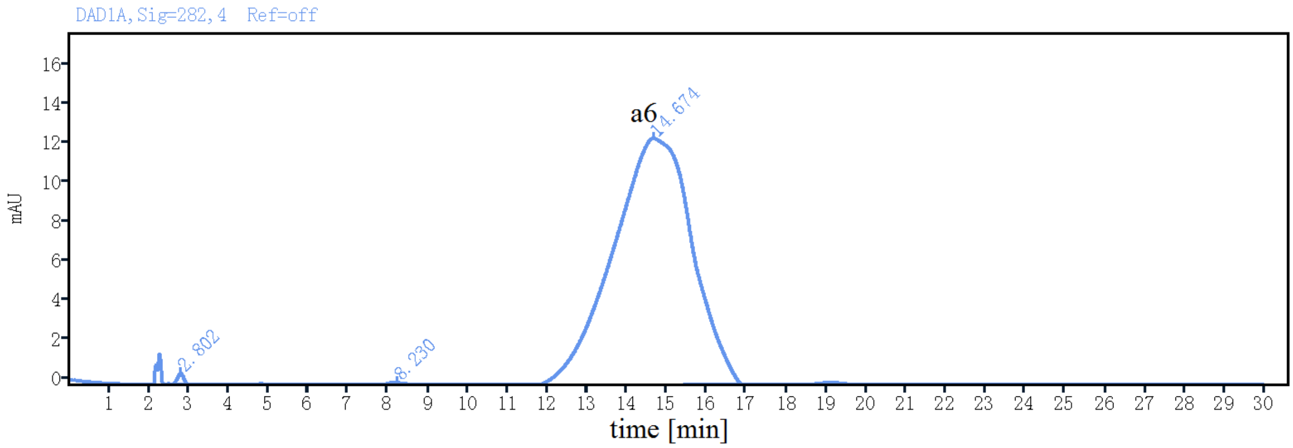

Retention time[min]	Area	Height	Area(%)
2. 802	7. 06	0. 61	0. 40
8. 230	8. 75	0. 21	0. 49
14. 674	1765. 65	12. 64	99. 11
	1781. 45		

*(E)-2-hydroxy-5-(3-(o-tolyl)acryloyl)benzamide (**a6**)*

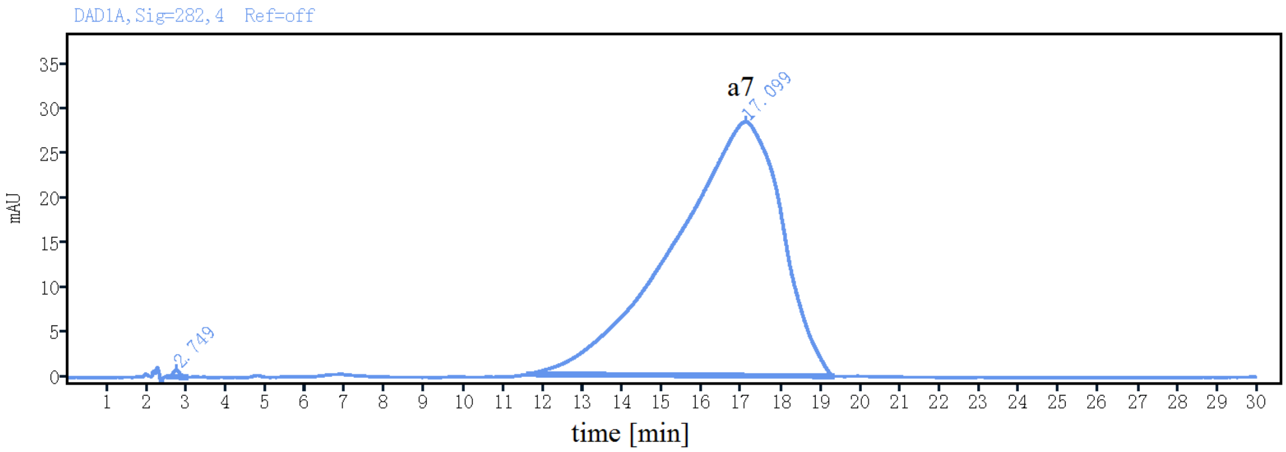

Retention time[min]	Area	Height	Area(%)
2.749	9.12	0.78	0.17
17.099	5384.61	28.45	99.83
	5393.73		

*(E)-2-hydroxy-5-(3-(m-tolyl)acryloyl)benzamide (**a7**)*

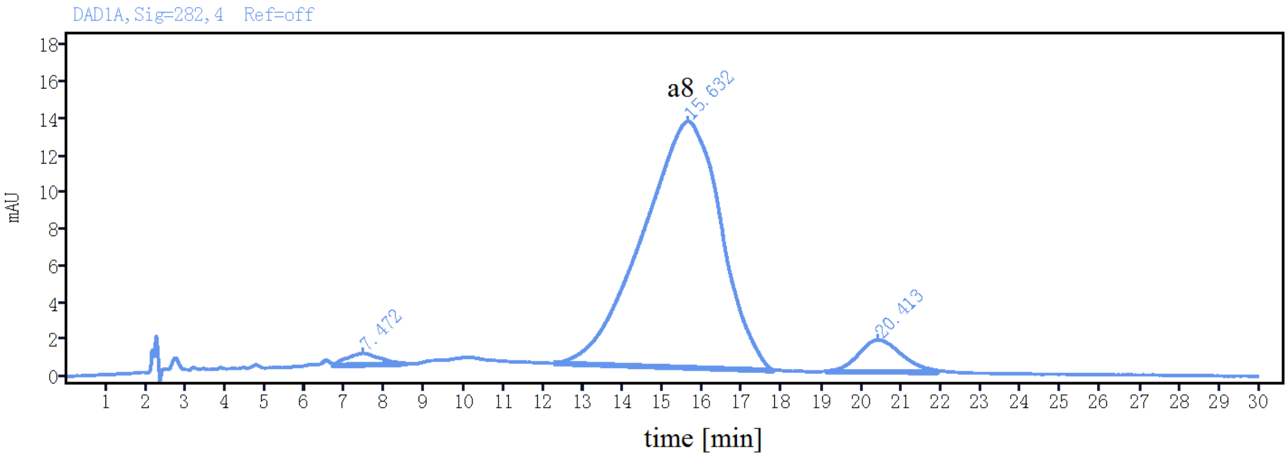

Retention time[min]	Area	Height	Area(%)
7.472	36.65	0.64	1.83
15.632	1834.75	13.39	91.55
20.413	132.60	1.75	6.62
	2004.00		

*(E)-2-hydroxy-5-(3-(p-tolyl)acryloyl)benzamide (**a8**)*

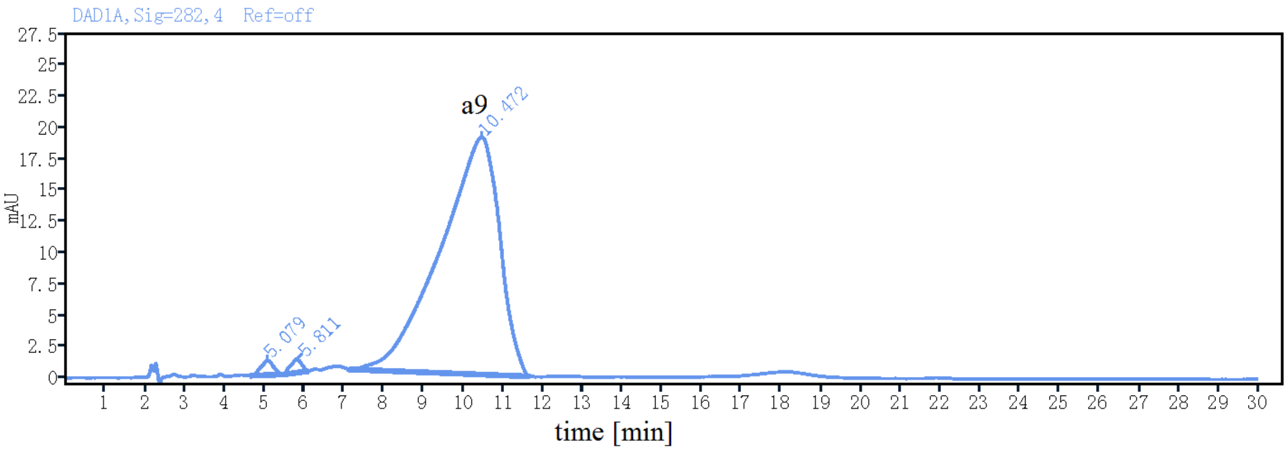

Retention time[min]	Area	Height	Area(%)
5.079	25.23	1.16	1.26
5.811	20.62	1.07	1.03
10.472	1960.23	18.97	97.71
	2006.08		

*(F)-2-hydroxy-5-(3-(2-methoxyphenyl)acryloyl)benzamide (**a9**)*

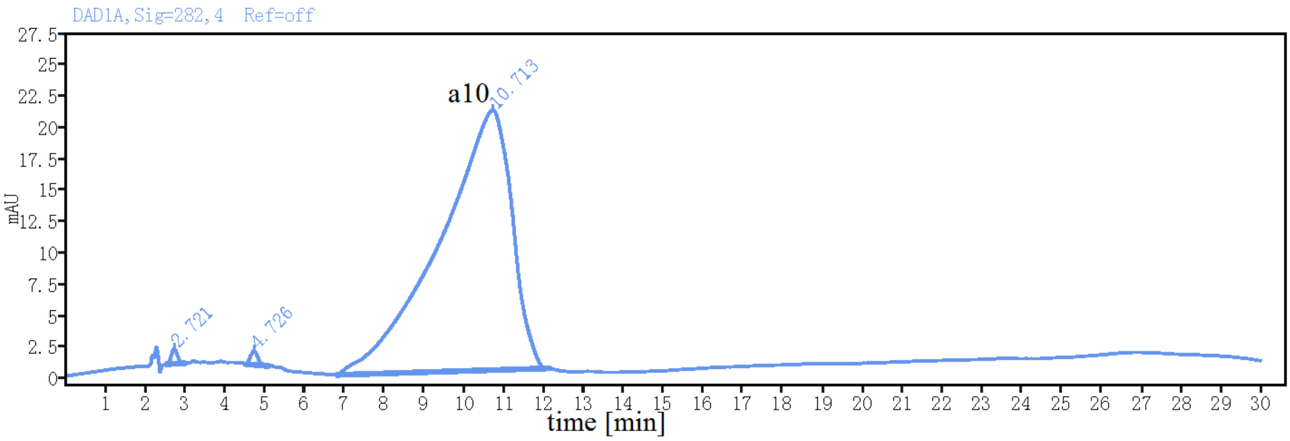

Retention time[min]	Area	Height	Area(%)
2.721	12.74	1.17	0.49
4.726	15.06	1.13	0.58
10.713	2589.88	20.72	98.94
	2617.69		

*(E)-2-hydroxy-5-(3-(3-methoxyphenyl)acryloyl)benzamide(**a10**)*

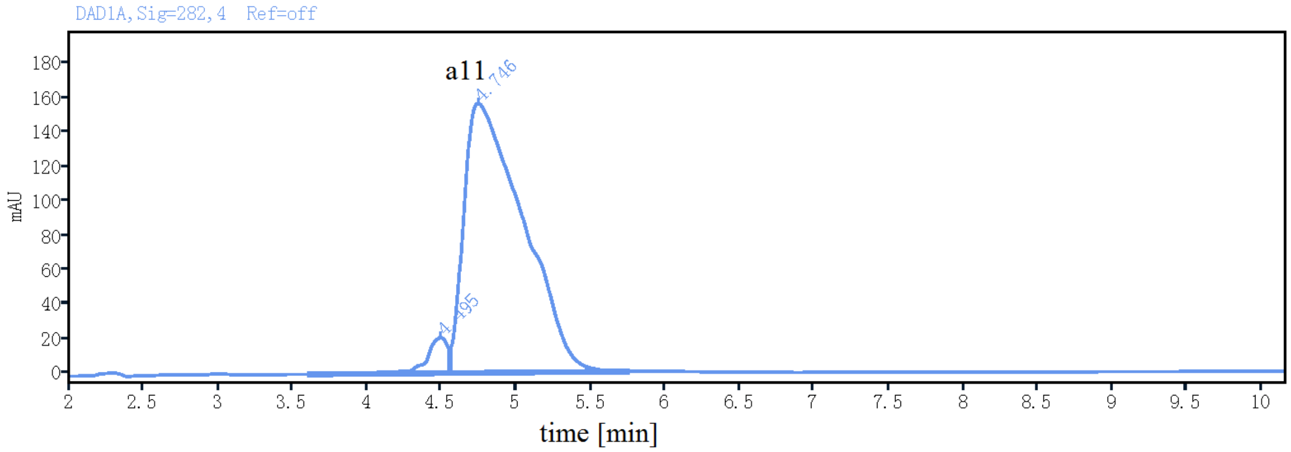

Retention time[min]	Area	Height	Area(%)
4. 495	205.00	20. 65	4. 53
4. 746	4323.94	156. 49	95. 47
	4528.94		

(F)*-2-hydroxy-5-(3-(4-methoxyphenyl)acryloyl)benzamide(**a11**)*

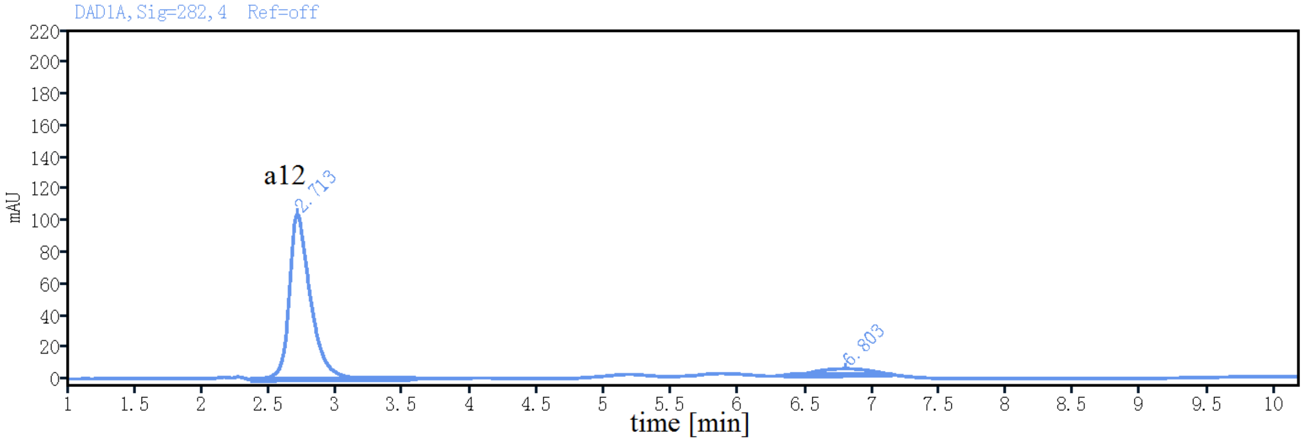

Retention time[min]	Area	Height	Area(%)
2. 713	1161. 95	104. 17	91. 27
6. 803	111. 16	4. 00	8. 73
	1273. 11		

*(E)-5-(3-(2,4-dimethoxyphenyl)acryloyl)-2-hydroxybenzamide (**a12**)*

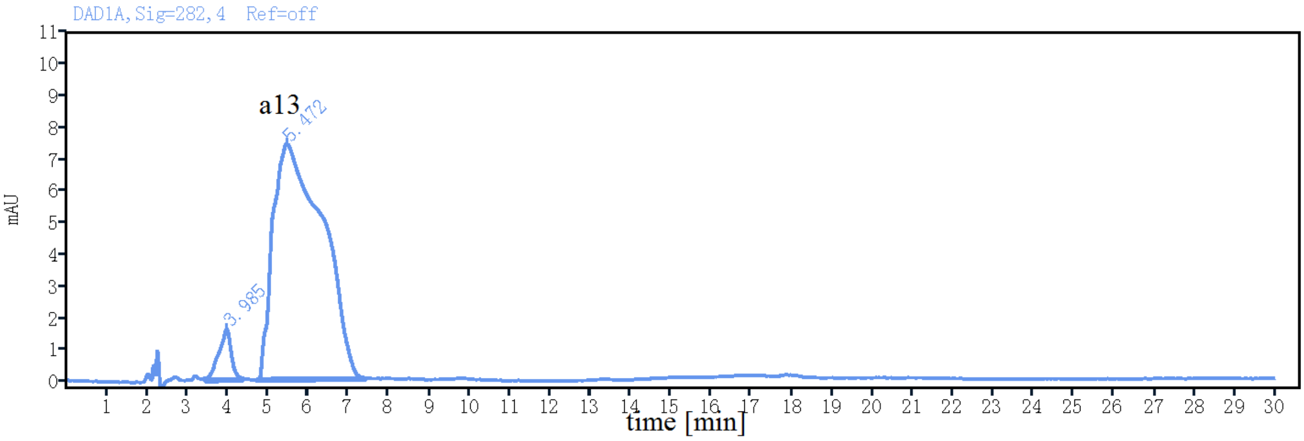

Retention time[min]	Area	Height	Area(%)
3.985	34.79	1.61	5.19
5.472	635.41	7.43	94.81
	670.19		

***(F)** -5-(3-(3,4-dimethoxyphenyl)acryloyl)-2-hydroxybenzamide (**a13**)*

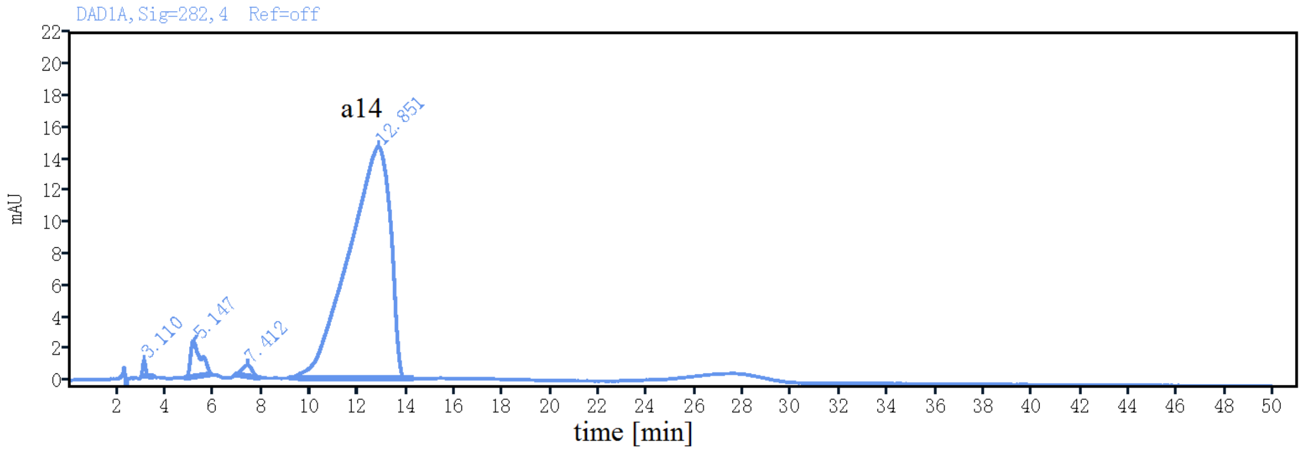

Retention time[min]	Area	Height	Area(%)
3.110	6.56	0.96	0.36
5.147	65.99	2.26	3.58
7.412	20.02	0.70	1.09
12.851	1751.58	14.67	94.98
	1844.15		

*(E)-2-hydroxy-5-(3-(3,4,5-trimethoxyphenyl)acryloyl)benzamide (**a14**)*
